# Antibacterial Conjugates of Kanamycin A with Vancomycin and Eremomycin: Biological Activity and a New MS-Fragmentation Pattern of Cbz-Protected Amines

**DOI:** 10.3390/antibiotics12050894

**Published:** 2023-05-11

**Authors:** Pavel N. Solyev, Elena B. Isakova, Evgenia N. Olsufyeva

**Affiliations:** 1 Engelhardt Institute of Molecular Biology, 32 Vavilov St., 119991 Moscow, Russia; 2 Gause Institute of New Antibiotics, 11 Bolshaya Pirogovskaya St., 119021 Moscow, Russia; ebisakova@yandex.ru

**Keywords:** antibacterial compounds, drug resistance, kanamycin A, vancomycin, eremomycin, hybrid antibiotics, fragmentation, rearrangement

## Abstract

A significant increase of microbial resistance to glycopeptides (especially vancomycin-resistant enterococci and *Staphylococcus aureus*) prompted researchers to design new semisynthetic glycopeptide derivatives, such as dual-action antibiotics that contain a glycopeptide molecule and an antibacterial agent of a different class. We synthesized novel dimeric conjugates of kanamycin A with glycopeptide antibiotics, vancomycin and eremomycin. Using tandem mass spectrometry fragmentation, UV, IR, and NMR spectral data, it was unequivocally proven that the glycopeptide is attached to the kanamycin A molecule at the position 1 of 2-deoxy-D-streptamine. New MS fragmentation patterns for *N*-Cbz-protected aminoglycosides were discovered. It was found that the resulting conjugates are active against Gram-positive bacteria, and some are active against vancomycin-resistant strains. Conjugates of two different classes can serve as dual-target antimicrobial candidates for further investigation and improvement.

## 1. Introduction

Vancomycin (**1**), first isolated from a strain of *Amycolatopsis orientalis* almost 70 years ago [1], has not so far lost its significance as a drug of the last resort, i.e., it is used for the treatment of serious life-threatening infections caused by Gram-positive bacteria unresponsive to other antibiotics and, especially, by methicillin-resistant *Staphylococcus aureus* (MRSA) and coagulase-negative staphylococci and enterococci [2]. Vancomycin is also used per os in *C*. *difficile* infections treatment [3,4].

Another antibiotic drug of this class, eremomycin (**2**), was discovered at the Gause Institute of New Antibiotics. It was isolated from *Amycolatopsis orientalis* subsp. *Eremomycini* and showed even three to five times higher efficacy against Gram-positive bacteria than vancomycin [5].

They bind to the *N*-acyl-d-Ala-d-Ala termini of peptidoglycan precursors by five bonds. As a result, the transglycosylation and transpeptidation steps in cell wall synthesis are inhibited. It is well known that this lack of structural integrity of the cell wall ultimately leads to the osmotic lysis of bacterial cells [6]. However, glycopeptide use is limited by the increasing resistance and their limited spectrum; native antibiotics **1** and **2** are neither active against Gram-negative bacteria, vancomycin-resistant entrococci (VRE) VanA and VanB, nor are they capable of suppressing *S. aureus* with an intermediate level of vancomycin resistance (VISA), as reported by Japanese researchers in 1997 [7]. Vancomycin-resistant enterococci (VRE), VanA and VanB, have the depsipeptide, d-Ala–d-Lac, with which the antibiotic does not bind, instead of the terminal fragment of the dipeptide d-Ala-d-Ala [8].

In 2002 the first documented report of clinical infection caused by *S. aureus* fully resistant to vancomycin was published in the United States [9]. The mechanism of resistance in VISA involves a complex reorganization of the cell wall metabolism, leading to a grossly thickened cell wall with reduced glycopeptide cross-linking [10,11].

The chemical transformation of glycopeptide antibiotics can change the spectrum of their antimicrobial action and even gain new biological activities [12]. For example, new semisynthetic analogues of vancomycin or eremomycin appeared to be active against vancomycin-resistant strains, VRE and VISA [13,14]. REDOR NMR showed that the introduction of a substituent residue into the glycopeptide on the peripheral region of the molecule does not disrupt the site of antibiotic binding to the *N*-acyl-d-Ala-d-Ala target, but on the contrary, it creates prerequisites for the additional binding of the semisynthetic antibiotic to other fragments of the cell wall peptidoglycan [15]. Other glycopeptide derivatives appeared to be active even against HIV, hepatitis C virus, and some other enveloped viruses, including SARS-CoV [16,17,18]. The covalent fusion of two known drugs/pharmacophores or the drug + delivery vehicle to form a unified heterodimer construct known as an antimicrobial hybrid is a promising strategy that has yielded several drug candidates [19,20,21].

A particularly feasible approach against multi-resistant pathogens is to select two or more highly active antibiotics as a basis for the design of hybrid analogues [22,23]. Some of the hybrid antibiotics based on aminoglycosides are able to restore the potential of the original antibiotics towards resistant pathogens [24]. For example, several new hybrid derivatives of glycopeptide antibiotics **1** or **2** combined with the macrolide antibiotic azithromycin [25] or benzoxaborol pharmacophoric group [26] were found to be active against VRE and VISA strains. Moreover, it is known that the amidation of the terminal COOH group of glycopeptides increases their antibacterial activity [13,14,26]. Currently, the modification with the formation of amides leads to the best results [27,28].

This hybrid molecule can exhibit different properties in vivo that cannot be predicted ahead, whether the result will be a drug or a prodrug and each of the two molecules of antibiotics will show their intrinsic properties. Noteworthy, in a hybrid compound, the properties of one molecule may prevail over the properties of another molecule. For example, in the case of hybrids of eremomycin or vancomycin with azithromycin, the properties of the glycopeptide antibiotic prevailed over the properties of azithromycin [24]. It was also shown that azithromycin as an amide substituent improved the antibacterial activity of the glycopeptide antibiotic against Gram-positive bacteria, but did not lead to the emergence of the activity against Gram-negative bacteria.

Glycopeptide antibiotics are only suitable for the treatment of Gram-positive bacterial infections but not for infections caused by Gram-negative bacteria, which possess intrinsic resistance to them.

At least three antimicrobial hybrids are currently in clinical trials: TNP-2092 (rifamycin + quinolizinone hybrid, against priority pathogens), TNP-2198 (rifamycin-nitroimidazole conjugate, against priority pathogens), and DNV3887 (oxazolidinone-quinolone hybrid against *Clostridium difficile*) [29].

The broad-spectrum aminoglycoside kanamycin A (KAN, **3**) can be regarded as a good candidate for coupling as the amino component. Kanamycin A being isolated from *Streptomyces kanamyceticus* [30] is one of the most important aminoglycoside antibiotics in clinical practice for the treatment of many infectious diseases caused by Gram-negative and Gram-positive bacteria. Kanamycin A has been approved since 1982 for the treatment of infectious diseases caused by *E*. *coli*, *Proteus* sp., *Enterobacter aerogenes*, *K*. *pneumoniae*, *Serratia marcescens*, and *Acinetobacter* spp. pathogens, as well as *M*. *tuberculosis*, including strains resistant to streptomycin, para-aminosalicylic acid, and isoniazid.

Glycopeptides and kanamycin A have fundamentally different mechanisms of action. Aminoglycosides are translation inhibitors that act on the 30S subunit of the bacterial ribosome, and due to protein synthesis inhibition, aminoglycosides are bactericidally active against Gram-positive and Gram-negative bacteria, making them broad-spectrum antibiotics.

It is known from the literature that the introduction of substituents in kanamycin A at position 1 of the 2-deoxystreptamine (2-DOS) residue does not lead to a decrease in activity, but it protects the antibiotic from the action of inactivating enzymes [31]. Therefore, we chose a strategy that allows a selective attachment of kanamycin A to the glycopeptide antibiotic through the position 1 of 2-DOS but not at the residues of 6-deoxy-6-amino-d-glucopyranose and 3-deoxy-3-amino-d-glucopyranose.

The modification of the carboxy group of glycopeptide antibiotics has proven itself well for two reasons. First, this reaction, regardless of the bulkiness of the introduced amine, usually proceeds in good yields and without the formation of by-products, which is important for the subsequent scaling of the production process. Second, in general, the amidation of a glycopeptide molecule does not disrupt the interaction of the antibiotic derivative with the target, leading to highly active compounds. Dalbavancin, a semi-synthetic derivative of amide-type glycopeptides, approved for use in medicine, can be regarded as a successful example of such a modification. In addition, a number of examples have shown that amide derivatives of eremomycin, in contrast to the original antibiotic with a free carboxyl group, exhibit good activity against a strain of the glycopeptide-intermediate-resistant *Staphylococcus aureus* (GISA) by thickening the cell wall. Moreover, in animal models, eremomycin carboxamides have been shown to exhibit fewer allergic reactions than eremomycin and vancomycin [27,32].

The choice of kanamycin A as the second component to obtain a hybrid molecule was justified by the fact that even bulky substituents (for example, adamantyl, azithromycin, etc.) do not significantly reduce the activity of glycopeptide antibiotics but sometimes even improve it against Gram-positive bacteria, including resistant strains [33]. The introduction of hydrophilic groups capable of protonation sometimes leads to the emergence of activity against Gram-negative bacteria [34,35] due to their enhanced ability to pass through the outer membrane of Gram-negative bacteria.

Herein, we describe the synthesis of some novel hybrid conjugates of the glycopeptide antibiotics vancomycin (**1**) or eremomycin (**2**) with kanamycin A (**3**) and report their antibacterial properties. We also discuss new benzyloxycarbonyl (Cbz) mass-spectroscopic fragmentation in aminoglycosides that has been revealed during the characterization of intermediate products in protected amines.

## 2. Results and Discussion

### 2.1. Synthesis of Hybrid Aantibiotic Conjugates

**Scheme 1:**
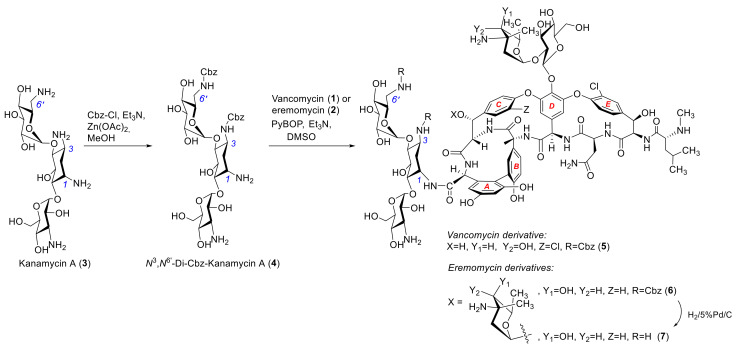
Synthesis of the hybrid conjugates of kanamycin A with vancomycin and eremomycin.

The novel hybrid compounds **5** and **6** were synthesized from vancomycin (**1**) and eremomycin (**2**) using the classic method [25,31] of glycopeptide carboxamide synthesis (Scheme 1). Kanamycin A can be selectively protected at amino groups at positions 3 and 6′, leaving axial 1-NH_2_ intact. Amide **4** was prepared in good yield according to the modified procedure based on the complexation of kanamycin A with Zn(OAc)_2_ and its further treatment with CbzCl in methanol in the presence of 1 eq. of Et_3_N [36]. *N*^3^,*N*^6′^-Di-benzyloxycarbonyl kanamycin A (**4**) was used as an amine component for the amidation reactions of **1** and **2**. For this purpose, the carboxy groups of antibiotics **1** or **2** were activated with (benzotriazol-1-yloxy)tripyrrolidinophosphonium hexafluorophosphate (PyBOP) and were treated with **4** to give *N*^3^,*N*^6′^-di-benzyloxycarbonyl-*N*^1^-kanamycinyl amides of vancomycin (**5**) or eremomycin (**6**), respectively (Scheme 1). The cleavage of Cbz groups in conjugates **5** and **6** was carried out in a flow of H_2_ on 5% Pd/C (1.5 atm, 2 h). In the case of the eremomycin derivative **6**, the cleavage of Cbz from amino groups in the kanamycin A residue proceeded satisfactorily with the formation of *N*^1^-eremomycin-kanamycin A amide (**7**). Noteworthy, the chlorine atom in the aromatic ring-system E did not suffer from the reduction, although it has been previously shown that under slightly more severe conditions (H_2_/5% Pd/C, 5 atm, 48 h), eremomycin (**2**) is quantitatively transformed into dechloroeremomycin [37]. However, the similar vancomycin derivative (**5**) cannot form the deprotected product; under these conditions it completely decomposes.

The synthesized products were purified by semi-preparative HPLC, analyzed for structural correspondence, and investigated for their preliminary antibacterial properties.

### 2.2. Structure Elucidation

The structure of the new derivatives was confirmed by a combination of physical methods and spectral characteristics. Hybrid derivatives **5**, **6**, and **7** demonstrated UV absorption spectra typical for glycopeptides; λ_max_ at 282 nm is characteristic for their phenolic groups. The IR spectra of the new derivatives show intense absorption bands in the region of 3300 cm^−1^ (NH/OH), ~1500 cm^−1^ (amide groups), and 1100–1000 cm^−1^ (CO carbohydrate residues) found in glycopeptides; di-Cbz products **5** and **6** have an additional intense absorption band at 1689 cm^−1^, corresponding to the O–C=O-group of the *N*-Cbz-protective group, which is slightly shifted in the parent *N*^3^,*N*^6′^-di-Cbz-kanamycin A (**4**) at 1694 cm^−1^ (Figure S24).

Structure elucidation via NMR spectra is hampered in **6** and **7** by the broadening of the multiple signals due to the large size of the hybrid antibiotics and their tendency to self-associate in DMSO solutions. Additional freeze-drying of the samples before dissolution did not affect the outcome of the spectra profiles. Nevertheless, it is possible to distinguish typical conservative regions for methyl groups at a region of 0.8–1.5 ppm and aromatic residues at 6.3–8.0 ppm in **5**, **6**, and **7** and to match the overall atom ratio by signal integration (Figure S10).

**Figure 1:**
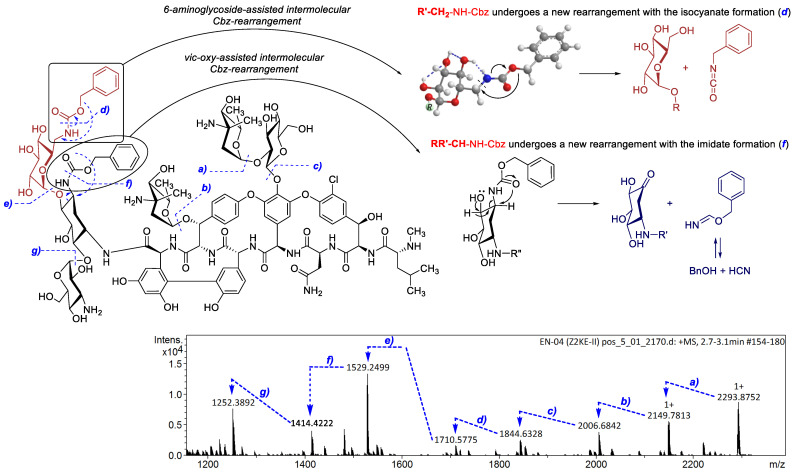
Fragmentation pathways and HRMS spectrum of the *N*-Cbz-protected compound **6**.

The determination of the coupling site of **5**, **6**, and **7** is crucial for such types of hybrid molecules. In this case, MS/MS fragmentation is a very useful tool for analysis and structural assignment. There are scarce examples described for ESI–MS fragmentation of hybrid structures based on glycopeptides **1** or **2** in the literature [24,25], but the method is appropriate for the core antibiotics **1** and **3** [38,39]. During the collision-induced fragmentation in HRMS, we observed consequent splits between different glycosidic moieties of the antibiotic dimer. We also found a previously undescribed rearrangement of the Cbz-amines at the primary carbon, assisted by the intermolecular sugar moiety hydrogen bonding (Figure 1).

We found that Cbz-groups in the products **4**, **5**, and **6** had a unique feature in the fragmentation, depending on the amine residue, which allows for the assignment of the type of substitution. The Cbz-protected amino group at the secondary carbon of the glycoside residue followed the typical fragmentation sequence with the neutral Cbz elimination, leaving the non-protected amino group. At the same time, the Cbz-amino group at the primary carbon atom experienced a new rearrangement, for which the intermediate state can be classified as a retro-Curtius-type/retro-Lossen-type rearrangement, but with the full inversion of the O-/N- linkage of the 6-aminoglycoside.

At the same time, later fragmentation leads to another rearrangement driven by the consequent elimination of the terminal 6-amino-6-deoxy-D-glucose moiety from 2-DOS. The latter undergoes the rearrangement, which resembles the recently proposed fragmentation mechanism for the loss of the C-2 primary fatty acid in the lipid polysaccharides, leading to the formation of a ketone group in the sugar ring. The mechanism was described by Aissa et al. [40]. In our case, the cleavage of the first sugar promoted a short-lived anionic form of the alcohol at position 4, which served as a driving force for intermolecular fragmentation with the release of the C-3 carbamate. However, instead of the earlier described ester oxygen switch to the sugar residue and isocyanate elimination, we observed the attack of the carbonyl oxygen of the Cbz driven by its short-lived anionic state at the C-3 position of 2-DOS. In this rearrangement, benzyl alohol formimidate was formed, which is readily decomposed into hydrogen cyanide and benzyl alcohol. As a result, 2-DOS substituted its amine to oxygen as in the earlier occured rearrangement, but giving ketone instead of the alcohol of the aminoglycoside.

The formed fragmentation ions were unequivocally confirmed by the well-calibrated high resolution MS-data. We witnessed a profound differentiation of Cbz-protected amines between primary and secondary carbon skeletons in aminoglycosides.

Gradual MS-fragmentation in products **4**–**7** indicated the attachment of **3** at the position 1 of deoxy-d-streptamine (the detailed fragmentation of each compound is given in the Supplementary Materials file). The alternative amidation reaction of the 3,6′-di-Cbz-kanamycin A (**4**) at the position of the 3”-amino group of the 3”-deoxy-3”-amino-D-glucopyranose residue was not possible because of the lack of the corresponding late-fragmentation ions containing monoamine-glycosides attached. Kanamycine A is a representative of the 2-deoxy-d-streptamine (2-DOS) containing antibiotics. The 2-DOS core is 4,6-disubstituted, and it remains intact in MS fragmentation up to the latest stages, proving that the binding position with eremomycin/vancomycin is definitely at the *N*^1^-KAN.

### 2.3. Antibacterial Activity

Hybrid antibiotic molecules based on two or more highly active antibiotics can provide a synergetic effect in their antipathogenic properties and even inhibit bacterial species resistant to the parent antibiotics [22,23]. We performed a study of antibacterial activity of the synthesized compounds **17** on a panel of clinical isolates of the Gram-positive microorganisms *S. haemolyticus* 602, *S. aureus* 3798 (VISA), and *Enterococcus faecalis* 560 (Van A) and against the Gram-negative strain *Escherichia coli* 25922 ATCC by the MTT method (Table 1) [24].

Hybrid dimers **5**–**7** appeared to be active against *S. haemoliticus* 602 (MIC = 1.0, 0.25, and 0.25 µg/mL); they demonstrated considerable activity against the resistant strain of *S. aureus* 3798 (VISA) (MIC = 4.0, 2.0, and 2.0 μg/mL). Of the studied samples, only the eremomycin-containing derivative **6** exhibited profound activity (MIC = 8 μg/mL) against the resistant strain of *E. faecalis* 560 (Van A), while other compounds were not active in this test. Natural Kanamycin A (**3**) and its di-Cbz-analogue (**4**) did not exhibit activity against all three strains of Gram-positive bacteria, as was expected. Activity against Gram-negative bacterium *E. coli* 25922 ATCC was found only in the starting kanamycin A (**3**) (MIC = 4 µg/mL); other antibiotics, **1** and **2**, as well as their hybrid analogues **5**–**7**, were not active in the test (MIC > 64 μg/mL).

As a result, the novel heterodimeric derivatives were obtained, and their structures were proven by spectral characteristics. Hybrid antibiotic compounds demonstrated profound antibacterial activity against Gram-positive bacteria, including vancomycin-resistant clinical isolates (VISA and VRE).

## 3. Materials and Methods

### 3.1. Reagents and Equipment

Vancomycin hydrochloride (**1**) was purchased from TEVA (TEVA Gyógyszergyár Zrt., Debrecen, Hungary). Eremomycin sulfate (**2**) was produced at the pilot plant of the Gause Institute of New Antibiotics. Kanamycin A sulphate (**3**) was purchased from Sigma-Aldrich (Merck KGaA, Darmstadt, Germany).

All reagents and solvents were purchased from Sigma-Aldrich (Merck KGaA, Darmstadt, Germany) and Merck (Merck KGaA, Darmstadt, Germany). The evaluation of the reactions, column eluates, and all final samples was analyzed by TLC using Merck Silica Gel 60F_254_ plates (Merck KGaA, Darmstadt, Germany). In addition, the purity of the final compounds was verified by HPLC data acquired on a Shimadzu LC-20 chromatograph with UV detector equipped with an AkzoNobel Kromasil 100-5C8 column (4.6 × 250 mm; 5 μm) (Akzo Nobel N.V., Amsterdam, the Netherlands). The column was eluted with a flow rate of 100 μL/min using two systems: System I with a mixture of acetonitrile (A) and ammonia formate 0.6% aq. solution, pH 7.8 (B), in a linear gradient of concentrations 8–70% A for 40 min; System II with a mixture of acetonitrile (A) and ammonia formate 0.2% aq. solution, pH 6.4 (B), in a linear gradient of concentrations 5–60% A for 35 min.

Reaction products were purified by reverse-phase column chromatography on Merck Silanized Silica Gel (0.063–0.2 mm) (Merck KGaA, Darmstadt, Germany). Melting points were determined using a Buchi SMP-20 apparatus.

UV spectra were determined by a UV spectra recorder of HPLC on Shimadzu LC-20 (Shimadzu Corporation, Kyoto, Japan).

IR spectra were obtained on a Nicolet-iS10 FTIR spectrometer (DTGS detector, splitter-KBr) (Thermo Scientific, Waltham, MA, USA) with a Smart Performer module equipped with a ZnSe-crystal. The spectra were run on the range of 3000–650 cm^−1^ with the resolution of 4 cm^−1^. The spectra were processed using the OMNIC-7.0 program package.

^1^H, ^13^C, and HSQC NMR spectra (δ, ppm; *J*, Hz) were registered on an Avance II spectrometer (Bruker BioSpin GmbH, Ettlingen, Germany) with the working frequency of 300 MHz for ^1^H NMR (Me_4_Si as an internal standard for organic solvents) and 75.5 MHz for ^13^C NMR (with carbon-proton interaction decoupling) at 27 °C. The spectra were processed using the MestReNova 14.2.1 package.

High-resolution mass spectra were recorded on a micrOTOF-Q II device (Bruker Daltonics, Bremen, Germany) by electrospray ionization mass spectrometry (ESI-MS). Measurements were carried out in positive ion mode; samples were injected into the mass-spectrometer chamber from an HPLC system Agilent 1260 (Agilent Technologies Inc., Santa Clara, CA, USA). The following parameters were used: capillary voltage 4500 V; mass scanning range: *m*/*z* 50–3000; external calibration with Electrospray Calibrant Solution (Fluka, Darmstadt, Germany); gas pressure 0.4 bar; nitrogen spray gas (6 L/min); interface temperature: 180 °C; flow rate 3 μL/min. Molecular and fragmentation ions in the spectra were analyzed and matched with the appropriately calculated *m*/*z* and isotopic profiles in the Bruker DataAnalysis 4.0 program. Earlier-prepared dry samples were dissolved in 50% acetonitrile in water and injected into the mass-spectrometer spray chamber from an Agilent 1260 HPLC chromatograph equipped with an Agilent Poroshell 120 EC-C18 column (3.0 × 50 mm; 2.7 μm) (Agilent Technologies Inc., Santa Clara, CA, USA) and a compatible pre-column cartridge using an autosampler. The column was eluted with a mixture of acetonitrile (A) and water (B) in a gradient concentration with a flow rate of 400 μL/min in the following gradient parameters: 0–15% A for 6 min, 15–85% A for 1.5 min, 85–0% A for 0.1 min, and 0% A for 2.4 min.

### 3.2. 3,6′-Di-Benzyloxycarbonyl-Kanamycin A (**4**)

Compound **4** was synthesized according to the method described previously [11], giving the white powder of **4**. Yield was 58%. The melting point was 182–184 °C (decomposed). HRMS (ESI) of C_34_H_48_N_4_O_15_, *m*/*z*: calcd for [M+H]^+^ 753.3189, found: 753.3190; calcd for [M+Na]^+^ 775.3008, found: 775.3015. For the detailed HRMS (ESI) fragmentation ions with isotopic profiles and program-calculated *m*/*z* exact mass comparison, UV, IR, and NMR spectra, see Figures S2–S7 in theSupplementary Materials file.

### 3.3. General Procedure for Synthesis of Conjugates 3,6′-Di-Cbz-Kanamycinyl A 1-Amides of Vancomycin (**5**) and Eremomycin (**6**)

Conjugates **5** and **6** were synthesized from vancomycin (**1**) or eremomycin (**2**) by the previously described method [10] for glycopeptide carboxamide synthesis by the condensation of **1** or **2** (1 eq.) with **4** (5 eq.), using PyBOP as a condensing reagent (1.5 eq.) in DMSO in the presence of Et_3_N (pH~8.5). After 18 h of stirring at 22 °C, Et_2_O was added to the reaction mixture, and it was shaken intensively to partly extract DMSO. The upper ethereal layer was separated, and the oil residue was dissolved in aqueous 0.1 M HCl. The pH value was adjusted to ~2, and the solution was poured into stirred acetone to precipitate the product. The product was filtered off, washed with acetone, and dried in a vacuum to obtain a white powder of crude salts **5** or **6.** The crude product was dissolved in H_2_O and applied on a column with silanized silica gel, pre-equilibrated with water (1 g of solid for 70 cm^3^ of silica gel).

### 3.4. 3,6′-Di-Cbz-Kanamycinyl A 1-Amide of Vancomycin (**5**)

The product was prepared according to the general procedure. The column elution was carried out with H_2_O, then with 0.01 N AcOH/H_2_O, then with the mixture of MeOH–H_2_O–AcOH (10:90:0.1) and MeOH–H_2_O–AcOH (20:80:0.1). Fractions containing the target compound were combined, and solvents were evaporated (with the addition of *n*-BuOH to lower the surface tension) to a small volume.

The addition of the mixture of acetone–Et_2_O (1:1) gave the precipitate, which was filtered, washed with acetone, and dried. Yield was 35% (counted on vancomycin) for the white powder of acetate of **5**. The melting point was 228–230 °C (decomposed). TLC was on Merck Silica Gel 60F_254_ plates in the EtOAc–PrOH–NH_4_OH (25% aq. sol-n) (4:4:1) system, R*_f_* = 0.21; in CHCl_3_–MeOH–NH_4_OH (25% aq. sol-n) (5:4:1) system, R*_f_* = 0.30. The retention time on HPLC (System I) was 29.59 min. HRMS (ESI) of C_100_H_121_Cl_2_N_13_O_38_, *m*/*z*: calcd for [M+H]^+^ 2182.7385, found: 2182.7359. For the detailed HRMS (ESI) fragmentation ions with isotopic profiles and program-calculated *m*/*z* exact mass comparison, UV, IR, and NMR spectra, see Figures S8–S22 in the Supplementary Materials file.

### 3.5. 3,6′-Di-Cbz-Kanamycinyl A 1-Amide of Eremomycin (**6**)

The product was prepared according to the general procedure. The column elution was carried out with H_2_O, then with 0.01 N AcOH/H_2_O. Fractions containing the target compound were combined, and solvents were evaporated (with the addition of *n*-BuOH to lower the surface tension) to a small volume. The addition of acetone gave the precipitate, which was filtered, washed with acetone, and dried. Yield was 55% (counted on eremomycin) for the white powder of acetate of **6**. The melting point was 210–213 °C (decomposed). TLC was on Merck Silica Gel 60F_254_ plates in the EtOAc–PrOH–NH_4_OH (25% aq. sol-n) (45:105:60) system, R*_f_* = 0.45; CHCl_3_–MeOH–NH_4_OH (25% aq. sol-n) (10:12:8) system, R*_f_* = 0.65. The retention time on HPLC (System I) was 36.66 min. HRMS (ESI) of C_107_H_135_ClN_14_O_40_, *m*/*z*: calcd for [M+H]^+^ 2291.8721, found: 2291.8720. For the detailed HRMS (ESI) fragmentation ions with isotopic profiles and program-calculated *m*/*z* exact mass comparison, UV, IR, and NMR spectra, see Figures S23–S36 in the Supplementary Materials file.

### 3.6. Kanamycinyl A 1-Amide of Eremomycin (**7**)

A catalytic amount of 5% Pd/C (300 mg) was added to a water–methanol (8:1) solution (45 mL) of **6** (150 mg, 0.062 mmol), and the reaction mixture was hydrogenated under H_2_ flow at a pressure of ~1.1 atm while stirring with a magnetic stirrer for 1 h. The precipitate of the catalyst was filtered off on a paper filter and washed with water (20 mL × 3). The mother liquors were combined, solvents were evaporated in vacuo at 40 °C (with the addition of *n*-BuOH to lower the surface tension) to a volume of ~1 mL, and acetone (20 mL) was added. The white precipitate was filtered, washed with acetone, and dried in vacuo. The yield was 70 mg (53%) for the white powder of acetate of **7**. The melting point was 238–240 °C (decomposed). Retention time for HPLC (System I) was 2.046 min (54%), 2.355 min (28%); ∑ = 82.546%. Retention time for HPLC (System II) was 20.589 min (31.581%), 21.924 min (24.069%), 22.842 min (17.732%), 23.947 min (7.865%), and 26.077 min (1.728%); ∑ = 82.975%. The HRMS (ESI) of C_91_H_123_ClN_14_O_36_, *m*/*z*: calcd for [M+H]^+^ 2023.7986, found: 2023.8016. For the detailed HRMS (ESI) fragmentation ions with isotopic profiles and program-calculated *m*/*z* exact mass comparison, UV, IR, and NMR spectra, see Figures S37–S50 in the Supplementary Materials file.

### 3.7. Microorganisms

The pre-screening of antibacterial activity of the new derivatives **4**–**7** and the starting antibiotics **1**–**3** was performed on a panel of clinical isolates of Gram-positive microorganisms *S. haemolyticus* 602, *S. aureus* 3798 (VISA), and *E. faecalis* 560 (Van A) (the strains were kindly provided by colleagues from the Lepetit Research Center (Lepetit Group, Biosearch Italia S.p.A., Gerenzano, Varese, Italy)) and the Gram-negative strain *E.coli* 25922 (a clinical isolate from ATCC).

The bacterial inoculum contents of 5 × 10^5^ CFU/mL were incubated for 24 h at 36 °C. The results were usually identical and always within two-fold of each other. The in vitro study was performed according to the recommendations of the European Committee on Antimicrobial Susceptibility testing. The MIC values for Gram-positive and Gram-negative bacterial strains were determined by the broth micro-dilution method using Mueller Hinton broth (Acumedia Manufacturers Inc., Lansing, MI, USA) and the National Committee for Clinical Laboratory Standards procedures as described earlier [33,41].

## 4. Conclusions

We presented a convenient synthesis of new hybrid dimeric conjugates of kanamycin A with glycopeptide antibiotics vancomycin and eremomycin. The compounds were characterized by UV, IR, and HRMS spectra. New MS-fragmentation patterns for Cbz-protected amines in aminoglycosides and 2-deoxy-d-streptamine were found and described, which allows for distinguishing two different cyclic systems. Preliminary testing revealed the antimicrobial activity of the conjugates against Gram-positive bacteria, including some activity against vancomycin-resistant strains. These findings may be further used for broadening the representative library of hybrid antibiotics and their MS characterization.

## Figures and Tables

**Table 1:** Antibacterial activity of compounds **17** against bacterial strains.

Compound	MIC (Minimum Inhibitory Antibiotic Concentration), µg/mL			
	*E. coli* 25922 ATCC	*S. haemoliticus* 602	*S. aureus* 3798 (VISA)	*E. faecalis* 560 (Van A)
Vancomycin (**1**)	>64	1	8	>64
Eremomycin (**2**)	64	0.25	16	>64
Kanamycin A (**3**)	4	>64	>64	64
**4**	>64	>64	>64	>64
**5**	>64	0.25	2	32
**6**	>64	1	4	8
**7**	>64	0.25	2	32

## Data Availability

Not applicable.

## Supplementary Materials

The following supporting information can be downloaded at: https://www.mdpi.com/article/10.3390/antibiotics12050894/s1, Figure S1: UV spectrum of **4**; Figure S2: IR spectra of **4**; Figure S3: ^1^H NMR spectrum of **4**; Figures S4–S7: HRMS spectra of **4**; Figure S8: UV spectrum of **5**; Figure S9: IR spectra of **5**; Figures S10 and S11: ^1^H and HSQC NMR spectra of **5**; Figures S12–S22: HRMS spectra of **5**; Figure S23: UV spectrum of **6**; Figure S24: IR spectra of **6**; Figure S25: ^1^H NMR spectrum of **6**; Figures S26–S36: HRMS spectra of **6**; Figure S37: UV spectrum of **7**; Figure S38: IR spectra of **7**; Figures S39 and S40: ^1^H and HSQC NMR spectra of **7**; Figures S41–S50: HRMS spectra of **7**; Figure S51–S53: ^1^H, ^13^C and HSQC NMR spectra of **1**.

## References

[1] McCormick M.H., McGuire J.M., Pittenger G.E., Pittenger R.C., Stark W.M. (1955). Vancomycin, a new antibiotic. I. Chemical and biologic properties. Antibiot. Annu..

[2] Binda E., Marinelli F., Marcone G.L. (2014). Old and new glycopeptide antibiotics: Action and resistance. Antibiotics.

[3] McDonald L.C., Gerding D.N., Johnson S., Bakken J.S., Carroll K.C., Coffin S.E., Dubberke E.R., Garey K.W., Gould C.V., Kelly C. (2018). Clinical practice guidelines for *Clostridium difficile* infection in adults and children: 2017 update by the Infectious Diseases Society of America (IDSA) and Society for Healthcare Epidemiology of America (SHEA). Clin. Infect. Dis..

[4] Stevens V.W., Khader K., Echevarria K., Nelson R.E., Zhang Y., Jones M., Timbrook T.T., Samore M.H., Rubin M.A. (2020). Use of oral vancomycin for *Clostridioides difficile* infection and the risk of vancomycin-resistant enterococci. Clin. Infect. Dis..

[5] Gause G.F., Brazhnikova M.G., Lomakina N.N., Berdnikova T.F., Fedorova G.B., Tokareva N.L., Borisova V.N., Batta G.Y. (1989). Eremomycin—New glycopeptide antibiotic: Chemical properties and structure. J. Antibiot..

[6] Nicolaou K.C., Boddy C.N.C., Brase S., Winssinger N. (1999). Chemistry, biology, and medicine of the glycopeptide antibiotics. Angew. Chem. Int. Ed..

[7] Hiramatsu K., Aritaka N., Hanaki H., Kawasaki S., Hosoda Y., Hori S., Fukuchi Y., Kobayashi I. (1997). Dissemination in Japanese hos-pitals of strains of Staphylococcus aureus heterogeneously resistant to vancomycin. Lancet.

[8] Bugg T.D.H., Wright G.D., Dutka-Malen S., Arthur M., Courvalin P., Walsh C.T. (1991). Molecular basis of vancomycin resistance in *Enterococcus faecium* BM4147: Biosynthesis of a depsipeptide peptidoglycan precursor by vancomycin resistance proteins VanH and VanA. Biochemistry.

[9] Centers for Disease Control and Prevention (2002). *Staphylococcus aureus* resistant to vancomycin—United States, 2002. MMWR Morb. Mortal. Wkly. Rep..

[10] Sieradzki K., Roberts R.B., Haber S.W., Tomasz A. (1999). The development of vancomycin resistance in a patient with methicillin-resistant *Staphylococcus aureus* infection. N. Engl. J. Med..

[11] Smith T.L., Pearson M.L., Wilcox K.R., Cruz C., Lancaster M.V., Robinson-Dunn B., Tenover F.C., Zervos M.J., Band J.D., White E. (1999). Emergence of vancomycin resistance in *Staphyococcus aureus*. N. Engl. J. Med..

[12] van Groesen E., Innocenti P., Martin N.I. (2022). Recent Advances in the Development of Semisynthetic Glycopeptide Antibiotics: 2014–2022. ACS Infect. Dis..

[13] Ashford P.-A., Bew S.P. (2012). Recent advances in the synthesis of new glycopeptide antibiotics. Chem. Soc. Rev..

[14] Olsufyeva E.N., Tevyashova A.N. (2017). Synthesis, Properties, and Mechanism of Action of New Generation of Polycyclic Glycopeptide Antibiotics. Curr. Top. Med. Chem..

[15] Kim S.J., Cegelski L., Preobrazhenskaya M., Schaefer J. (2006). Structures of *Staphylococcus aureus* cell-wall complexes with vancomycin, eremomycin, and chloroeremomycin derivatives by ^13^C{^19^F} and ^15^N{^19^F} rotational-echo double resonance. Biochemistry.

[16] Balzarini J., Pannecouque C., De Clercq E., Pavlov A.Y., Printsevskaya S.S., Miroshnikova O.V., Reznikova M.I., Preobrazhenskaya M.N. (2003). Antiretroviral activity of semisynthetic derivatives of glycopeptide antibiotics. J. Med. Chem..

[17] Balzarini J., Keyaerts E., Vijgen L., Egberink H., De Clercq E., Van Ranst M., Printsevskaya S.S., Olsufyeva E.N., Solovieva S.E., Preobrazhenskaya M.N. (2006). Inhibition of feline (FIPV) and human (SARS) coronavirus by semisynthetic derivatives of glycopeptide antibiotics. Antivir. Res..

[18] Bereczki I., Papp H., Kuczmog A., Madai M., Nagy V., Agócs A., Batta G., Milánkovits M., Ostorházi E., Mitrović A. (2021). Natural Apocarotenoids and Their Synthetic Glycopeptide Conjugates Inhibit SARS-CoV-2 Replication. Pharmaceuticals.

[19] Pokrovskaya V., Baasov T. (2010). Dual-acting hybrid antibiotics: A promising strategy to combat bacterial resistance. Expert Opin. Drug Discovery.

[20] Parkes A.L., Yule I.A. (2016). Hybrid antibiotics—Clinical progress and novel designs. Expert Opin. Drug Discov..

[21] Klahn P., Bronstrup M. (2017). Bifunctional antimicrobial conjugates and hybrid antimicrobials. Nat. Prod. Rep..

[22] Tevyashova A.N., Olsufyeva E.N., Preobrazhenskaya M.N. (2015). Design of dual action antibiotics as an approach to search for new promising drugs. Russ. Chem. Rev..

[23] Domalaon R., Idowu T., Zhanel G.G., Schweizer F. (2018). Antibiotic Hybrids: The Next Generation of Agents and Adjuvants against Gram-Negative Pathogens?. Clin. Microbiol. Rev..

[24] Tevyashova A.N., Bychkova E.N., Korolev A.M., Isakova E.B., Mirchink E.P., Osterman I.A., Erdei R., Szücs Z., Batta G. (2019). Synthesis and evaluation of biological activity for dual-acting antibiotics on the basis of azithromycin and glycopeptides. Bioorg. Med. Chem. Lett..

[25] Printsevskaya S.S., Reznikova M.I., Korolev A.M., Lapa G.B., Olsufyeva E.N., Preobrazhenskaya M.N., Plattner J.J., Zhang Y.-K. (2013). Synthesis and study of antibacterial activities of antibacterial glycopeptide antibiotics conjugated with benzoxaboroles. Future Med. Chem..

[26] Moiseenko E.I., Grammatikova N.E., Shchekotikhin A.E. (2019). Eremomycin Picolylamides and Their Cationic Lipoglycopeptides: Synthesis and Antimicrobial Properties. Macroheterocycles.

[27] Olsufyeva E.N., Shchekotikhin A.E., Bychkova E.N., Pereverzeva E.R., Treshalin I.D., Mirchink E.P., Isakova E.B., Chernobrovkin M.G., Kozlov R.S., Dekhnich A.V. (2018). Eremomycin pyrrolidide: A novel semisynthetic glycopeptide with improved chemotherapeutic properties. Drug Des. Devel. Ther..

[28] Moiseenko E.I., Erdei R., Grammatikova N.E., Mirchink E.P., Isakova E.B., Pereverzeva E.R., Batta G., Shchekotikhin A.E. (2021). Aminoalkylamides of eremomycin exhibit an improved antibacterial activity. Pharmaceuticals.

[29] World Health Organization—Global Observatory on Health Research and Development (2022). Antibacterial Products in Clinical Development for Priority Pathogens. https://www.who.int/observatories/global-observatory-on-health-research-and-development/monitoring/antibacterial-products-in-clinical-development-for-priority-pathogens.

[30] Umezawa H. (1957). Production and isolation of a new antibiotic, kanamycin. J. Antibiot..

[31] Chandrika N.T., Garneau-Tsodikova S. (2018). Comprehensive review of chemical strategies for the preparation of new aminoglycosides and their biological activities. Chem. Soc. Rev..

[32] Pavlov A.Y., Berdnikova T.F., Olsufyeva E.N., Miroshnikova O.V., Filipposyanz S.T., Preobrazheskaya M.N., Sottani C., Colombo L., Goldstein B.P. (1996). Carboxamides and hydrazide of eremomycin. J. Antibiotics.

[33] Maples K.R., Wheeler C., Ip E., Plattner J., Chu D., Zhang Y.-K., Preobrazhenskaya M.N., Printsevskaya S.S., Solovieva S.E., Olsufyeva E.N. (2007). Novel semisynthetic derivative of antibiotic eremomycin active against drug-resistant Gram-positive pathogens including *Bacillus anthracis*. J. Med. Chem..

[34] Hanckok R.E.W., Farmer S.W. (1993). Mechanism of uptake of deglucoteicoplanin amide derivatives across outer membranes of Escherichia coli and Pseudomonas aeruginosa. Antimicrob. Agents Chemother..

[35] Yarlagadda V., Manjunath G.B., Sarkar P., Akkapeddi P., Paramanandham K., Shome B.R., Ravikumar R., Haldar J. (2016). Glycopeptide antibiotic to overcome the intrinsic resistance of gramnegative bacteria. ACS Infect. Dis..

[36] Umezawa H., Umezawa S., Tsuchiya T., Takagi Y., Jikihara T. (1981). Production of a Selectively Protected N-Acylated Derivative of an Aminoglycosidic Antibiotic. U.S. Patent.

[37] Berdnikova T.F., Lomakina N.N., Olsufyeva E.N., Aleksandrova L.G., Potapova N.P., Rozynov B.V., Malkova I.V., Orlova G.I. (1999). Structure antibacterial activity relationships of the products of degradation of antibiotic eremomycin. Antibiot. Chemother..

[38] Cao M., Feng Y., Zhang Y., Kang W., Lian K., Ai L. (2018). Studies on the metabolism and degradation of vancomycin in simulated in vitro and aquatic environment by UHPLC-Triple-TOF-MS/MS. Sci. Rep..

[39] Kotretsou S.I., Constantinou-Kokotou V. (1998). Mass spectrometric studies on the fragmentation and structural characterization of aminoacyl derivatives of kanamycin A. Carbohydr. Res..

[40] Aissa I., Kilár A., Dörnyei Á. (2021). Study on the CID Fragmentation Pathways of Deprotonated 4′-Monophosphoryl Lipid A. Molecules.

[41] Pavlov A.Y., Lazhko E.I., Preobrazhenskaya M.N. (1997). A new type of chemical modification of glycopeptides antibiotics: Aminomethylated derivatives of eremomycin and their antibacterial activity. J. Antibiot..

