# Supplementary material for: Antibacterial Conjugates of Kanamycin A with Vancomycin and Eremomycin: Biological Activity and a New MS-Fragmentation Pattern of Cbz-Protected Amines
Source: Antibiotics (Basel). 2023 May 11;12(5):894. doi: 10.3390/antibiotics12050894 (PMC10215198; doi:10.3390/antibiotics12050894)

ELECTRONIC SUPPLEMENTARY MATERIAL

Antibacterial conjugates of kanamycin A with vancomycin and eremomycin: biological activity and a new MS-fragmentation pattern of Cbz-protected amines

Pavel N. Solyev [1,*], Elena B. Isakova [2], Evgenia N. Olsufyeva [2,*]

[1] Engelhardt Institute of Molecular Biology, 32 Vavilov St., 119991 Moscow, Russia
[2] Gause Institute of New Antibiotics, 11 Bolshaya Pirogovskaya St., 119021 Moscow, Russia
* Correspondence: solyev@gmail.com (P.N.S.); eolsufeva@list.ru (E.N.O).

UV, FTIR, NMR and HRMS spectra of the compounds:

Figure S1. UV spectrum of 3,6'-di-benzyloxycarbonyl-kanamycin A (**4**)---------------- 2
Figure S2. IR spectra of 3,6'-di-benzyloxycarbonyl-kanamycin A (**4**) -------------------- 2
Figure S3. ^{1}H NMR spectrum of 3,6'-di-benzyloxycarbonyl-kanamycin A (**4**)--------- 3
Figures S4-S7. HRMS spectra of 3,6'-di-benzyloxycarbonyl-kanamycin (**4**) ------------ 4-7
Figure S8. UV spectrum of 3,6'-di-Cbz-kanamycinyl A 1-amide of vancomycin (**5**) - 8
Figure S9. IR spectra of 3,6'-di-Cbz-kanamycinyl A 1-amide of vancomycin (**5**)------ 8
Figure S10. ^{1}H NMR spectrum of 3,6'-di-Cbz-kanamycinyl A 1-amide of vancomycin (**5**) -- 9
Figure S11. HSQC NMR spectrum of 3,6'-di-Cbz-kanamycinyl A 1-amide of vancomycin (**5**) -- 9
Figures S12-S22. HRMS spectra of 3,6'-di-Cbz-kanamycinyl A 1-amide of vancomycin (**5**) -- 10-20
Figure S23. UV spectrum of 3,6'-di-Cbz-kanamycinyl A 1-amide of eremomycin (**6**) 21
Figure S24. IR spectra of 3,6'-di-Cbz-kanamycinyl A 1-amide of eremomycin (**6**) ---- 21
Figure S25. ^{1}H NMR spectrum of 3,6'-di-Cbz-kanamycinyl A 1-amide of eremomycin (**6**) -- 22
Figures S26-S36. HRMS spectra of 3,6'-di-Cbz-kanamycinyl A 1-amide of eremomycin (**6**) -- 23-33
Figure S37. UV spectra of kanamycinyl A 1-amide of eremomycin (**7**)------------------ 34
Figure S38. IR spectra of kanamycinyl A 1-amide of eremomycin (**7**) ------------------- 34
Figure S39. ^{1}H NMR spectrum of kanamycinyl A 1-amide of eremomycin (**7**)-------- 35
Figure S40. HSQC NMR spectrum of kanamycinyl A 1-amide of eremomycin (**7**) -- 35
Figures S41-S50. HRMS spectra of kanamycinyl A 1-amide of eremomycin (**7**) ------ 36-45
Figure S51. ^{1}H NMR spectrum of vancomycin (**1**) --- 46
Figure S52. ^{13}C NMR spectrum of vancomycin (**1**) -- 46
Figure S53. HSQC NMR spectrum of vancomycin (**1**)--------------------------------------- 47

3,6'-Di-benzyloxycarbonyl-kanamycin A (4)

Figure S1. UV-spectrum of 3,6'-di-benzyloxycarbonyl-kanamycin A (**4**)

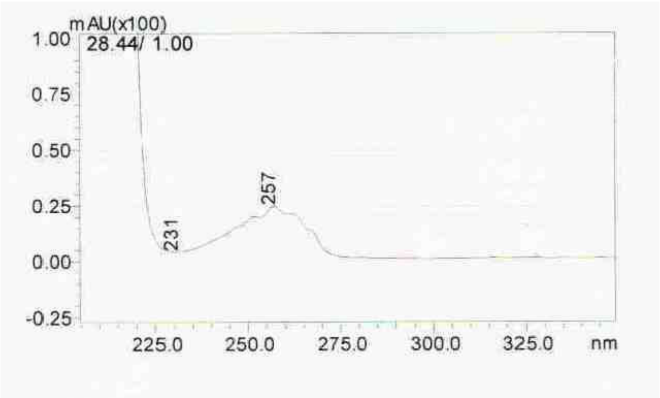

Figure S2. IR-spectra of 3,6'-di-benzyloxycarbonyl-kanamycin A (**4**)

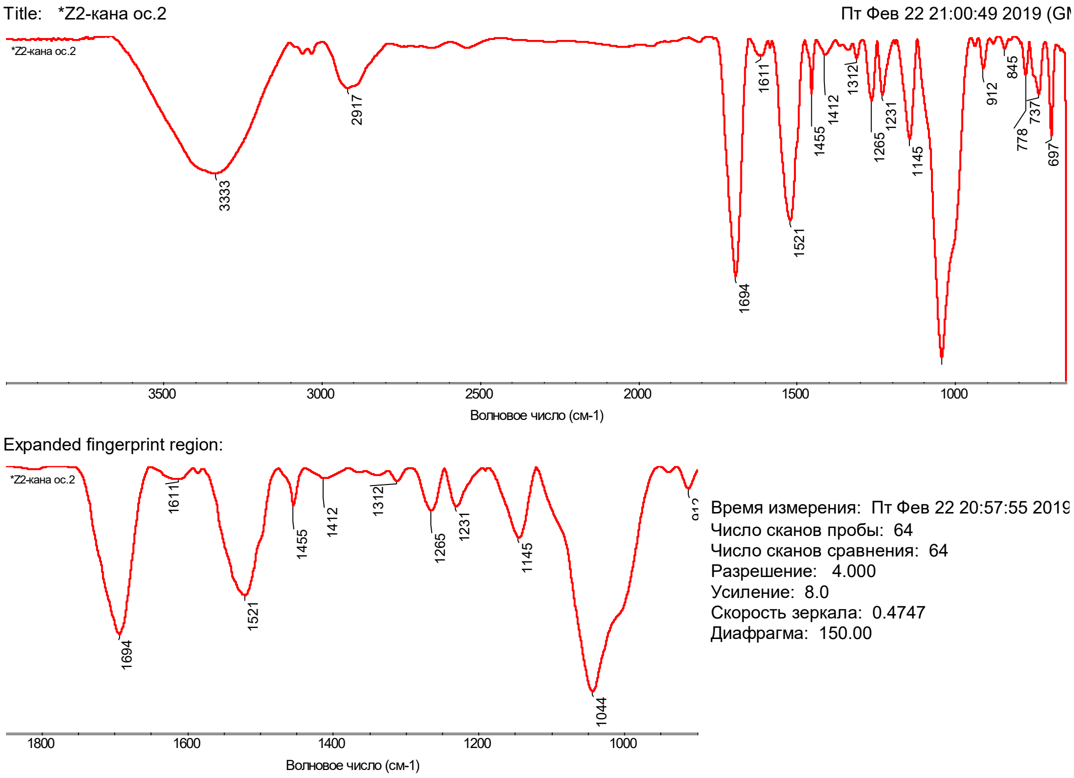

Figure S3. 1H NMR spectrum of 3,6'-di-benzyloxycarbonyl-kanamycin A (**4**)

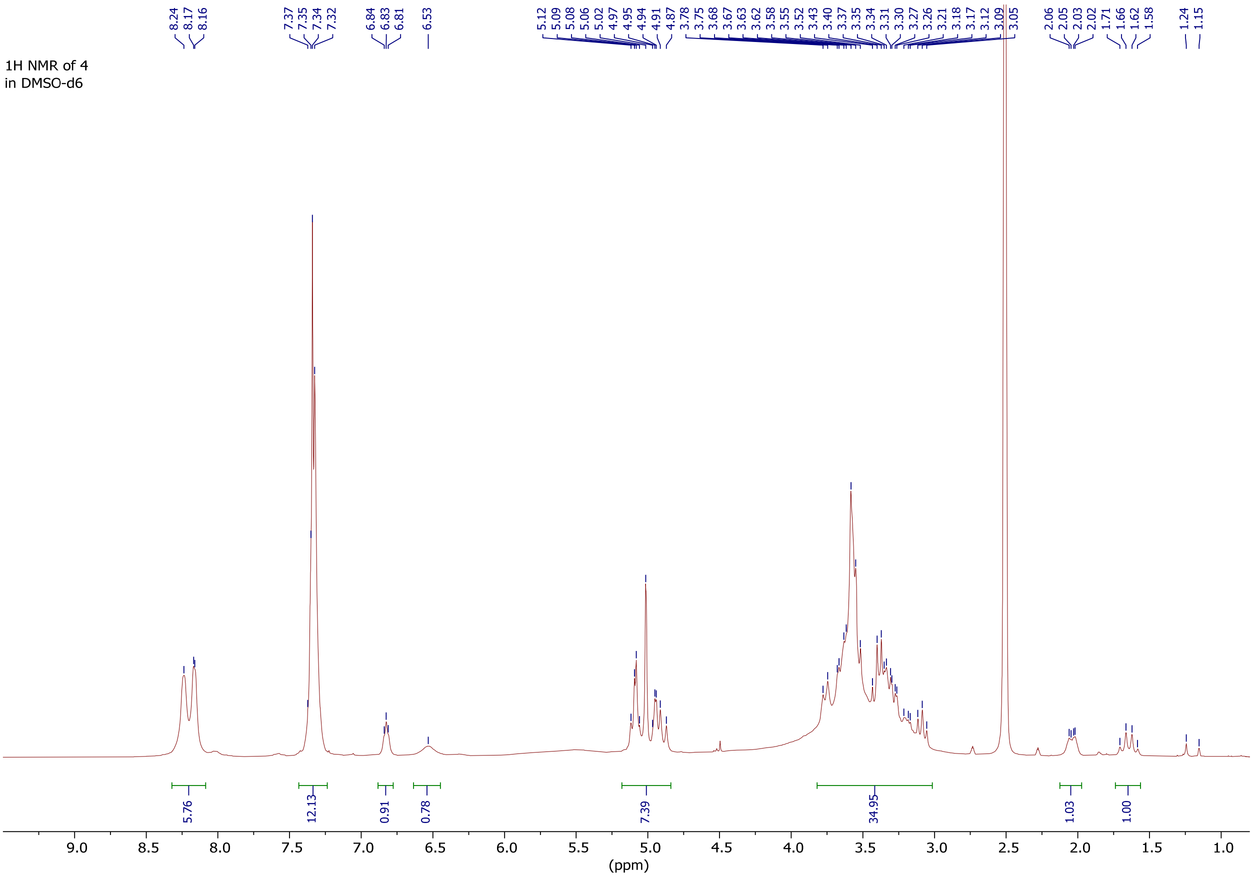

Figure S4. HRMS spectrum of 3,6'-di-benzyloxycarbonyl-kanamycin A (**4**)

Molecular ion $[M+H]^+$, $[M+Na]^+$ (m/z, z=1):

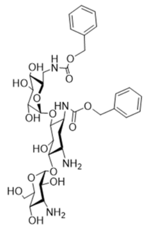

Display Report

Analysis Info

Analysis Name	D:\Data\EN-01 (Z2K) pos_2_01_2167.d	Acquisition Date	10/22/2018 3:43:43 PM
Method	la-2.2-energy.m	Operator	BDAL@DE
Sample Name	EN-01 (Z2K) pos	Instrument	compact 8255754.20088
Comment			

Acquisition Parameter

Source Type	ESI	Ion Polarity	Positive	Set Nebulizer	0.4 Bar
Focus	Active	Set Capillary	4500 V	Set Dry Heater	180 °C
Scan Begin	50 m/z	Set End Plate Offset	-500 V	Set Dry Gas	6.0 l/min
Scan End	3000 m/z	Set Charging Voltage	2000 V	Set Divert Valve	Source
		Set Corona	0 nA	Set APCI Heater	0 °C

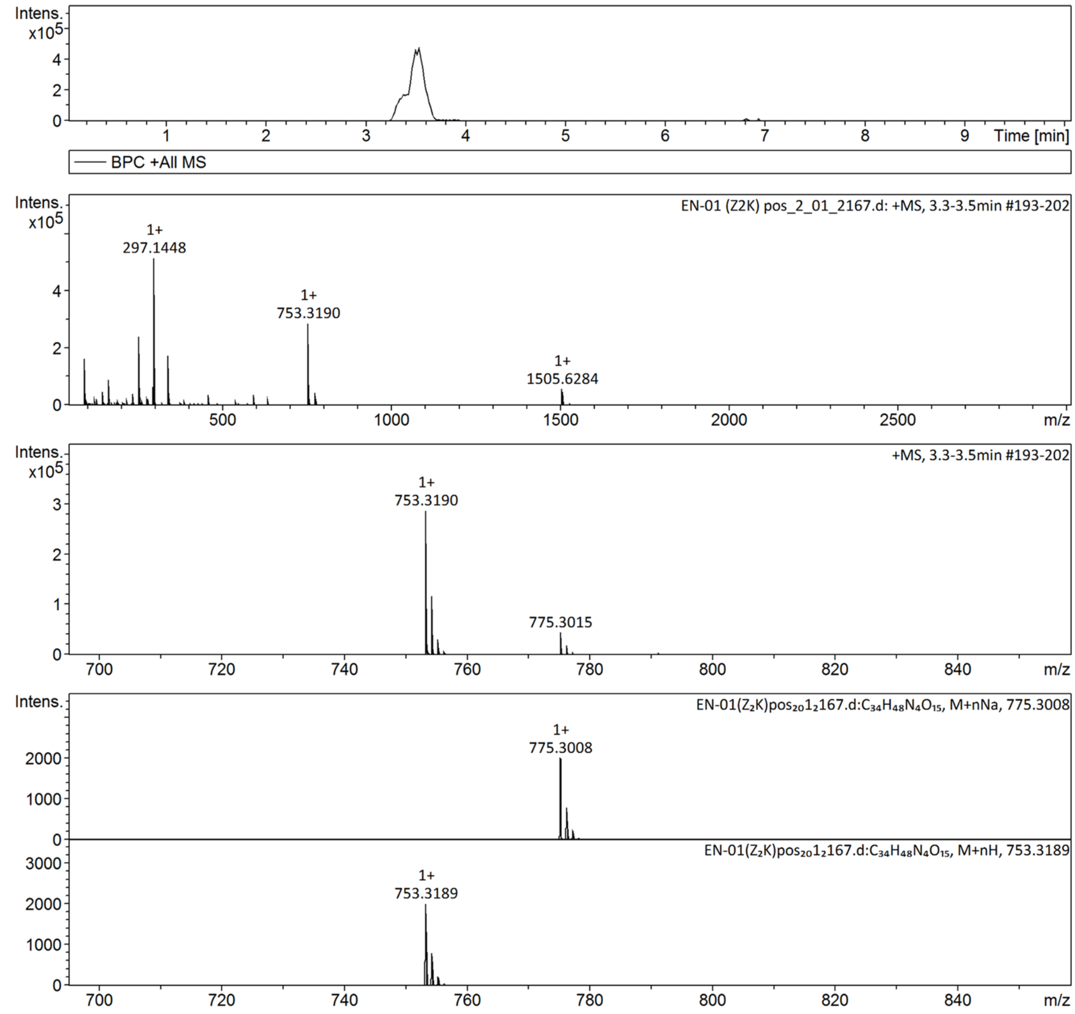

Figure S5. HRMS spectrum of 3,6'-di-benzyloxycarbonyl-kanamycin A (**4**)

Fragmentation ions $[M+H]^+$:

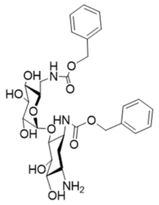

and

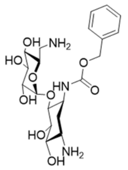

Display Report

Analysis Info

Analysis Name	D:\Data\EN-01 (Z2K) pos_2_01_2167.d	Acquisition Date	10/22/2018 3:43:43 PM
Method	la-2.2-energy.m	Operator	BDAL@DE
Sample Name	EN-01 (Z2K) pos	Instrument	compact 8255754.20088
Comment			

Acquisition Parameter

Source Type	ESI	Ion Polarity	Positive	Set Nebulizer	0.4 Bar
Focus	Active	Set Capillary	4500 V	Set Dry Heater	180 °C
Scan Begin	50 m/z	Set End Plate Offset	-500 V	Set Dry Gas	6.0 l/min
Scan End	3000 m/z	Set Charging Voltage	2000 V	Set Divert Valve	Source
		Set Corona	0 nA	Set APCI Heater	0 °C

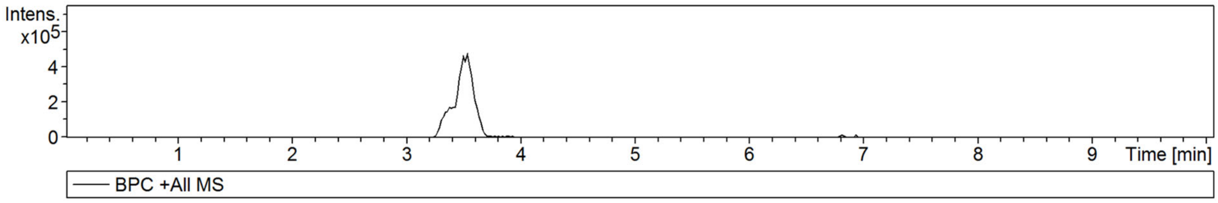

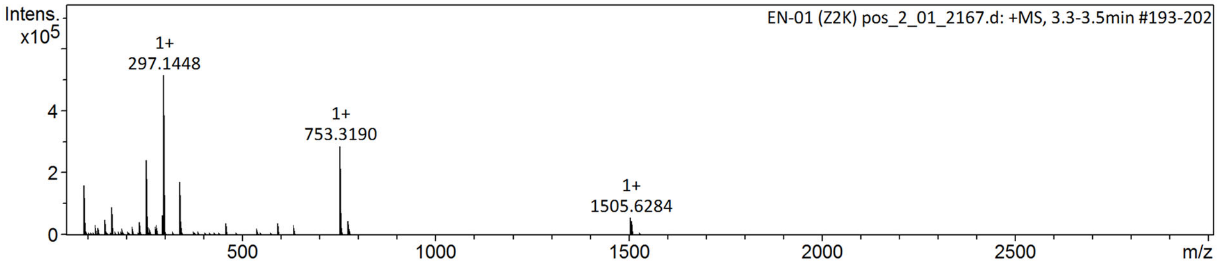

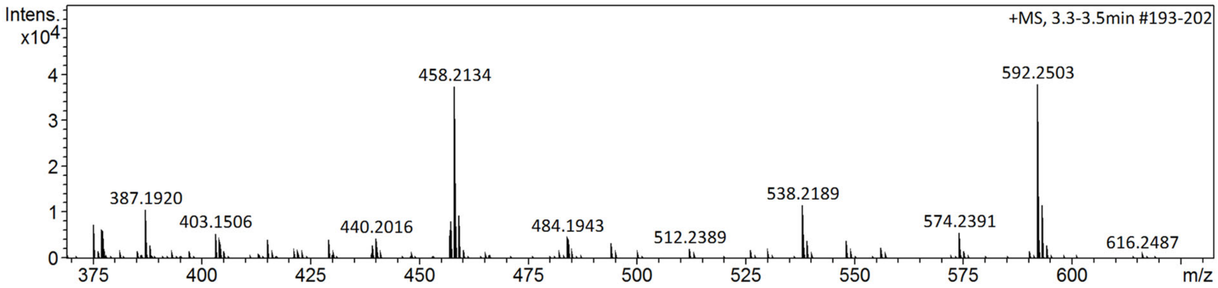

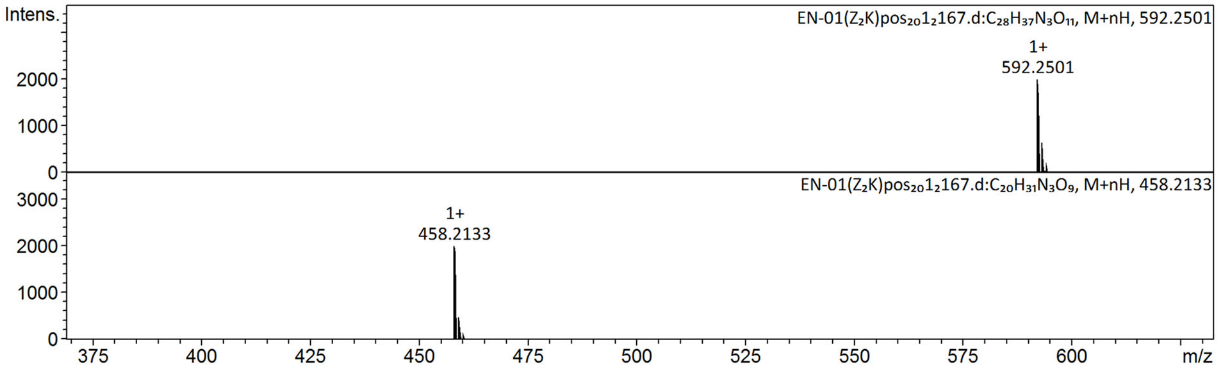

Figure S6. HRMS spectrum of 3,6'-di-benzyloxycarbonyl-kanamycin A (**4**)

Fragmentation ions $[M+H]^+$:

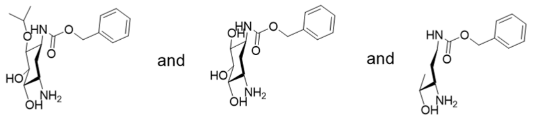

Display Report

Analysis Info

Analysis Name	D:\Data\EN-01 (Z2K) pos_2_01_2167.d	Acquisition Date	10/22/2018 3:43:43 PM
Method	la-2.2-energy.m	Operator	BDAL@DE
Sample Name	EN-01 (Z2K) pos	Instrument	compact 8255754.20088
Comment			

Acquisition Parameter

Source Type	ESI	Ion Polarity	Positive	Set Nebulizer	0.4 Bar
Focus	Active	Set Capillary	4500 V	Set Dry Heater	180 °C
Scan Begin	50 m/z	Set End Plate Offset	-500 V	Set Dry Gas	6.0 l/min
Scan End	3000 m/z	Set Charging Voltage	2000 V	Set Divert Valve	Source
		Set Corona	0 nA	Set APCI Heater	0 °C

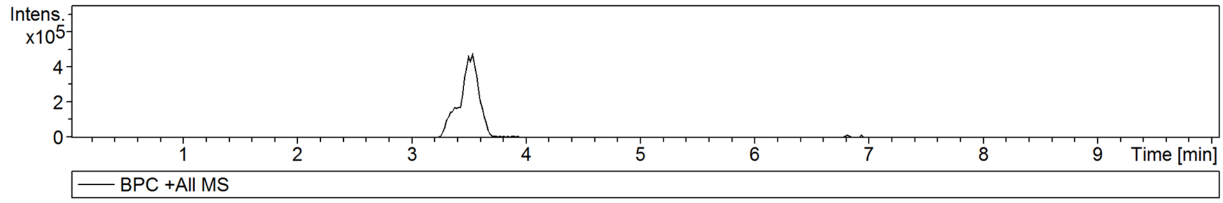

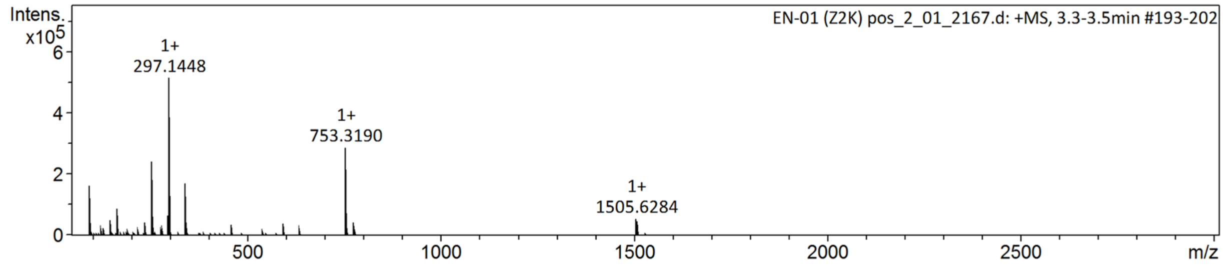

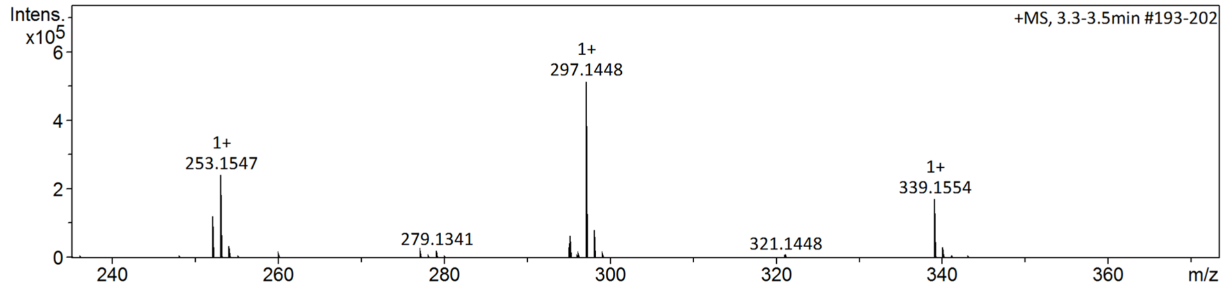

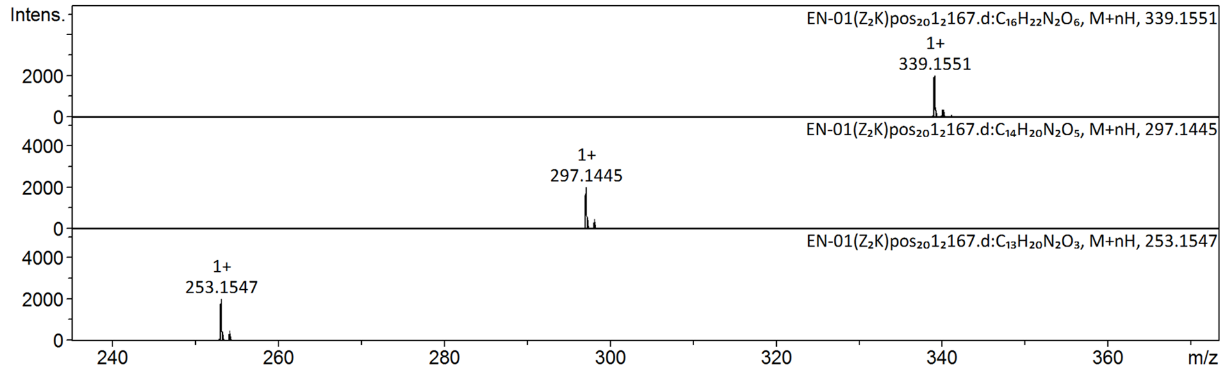

Figure S7. HRMS spectrum of 3,6'-di-benzyloxycarbonyl-kanamycin A (**4**)

Fragmentation ions $[M+H]^+$ and $[tropylium]^+$:

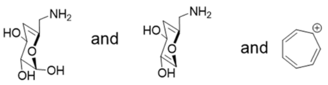

Display Report

Analysis Info

Analysis Name	D:\Data\EN-01 (Z2K) pos_2_01_2167.d	Acquisition Date	10/22/2018 3:43:43 PM
Method	la-2.2-energy.m	Operator	BDAL@DE
Sample Name	EN-01 (Z2K) pos	Instrument	compact 8255754.20088
Comment			

Acquisition Parameter

Source Type	ESI	Ion Polarity	Positive	Set Nebulizer	0.4 Bar
Focus	Active	Set Capillary	4500 V	Set Dry Heater	180 °C
Scan Begin	50 m/z	Set End Plate Offset	-500 V	Set Dry Gas	6.0 l/min
Scan End	3000 m/z	Set Charging Voltage	2000 V	Set Divert Valve	Source
		Set Corona	0 nA	Set APCI Heater	0 °C

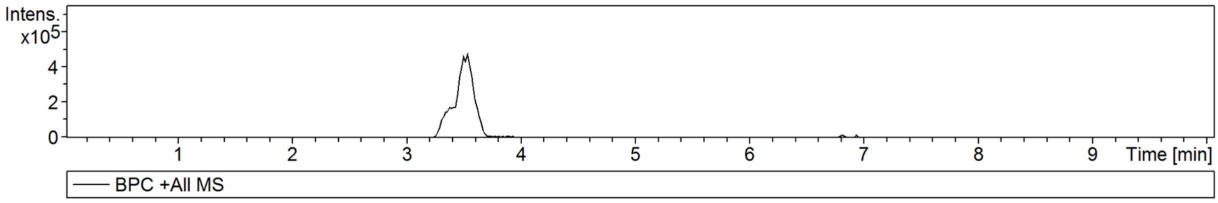

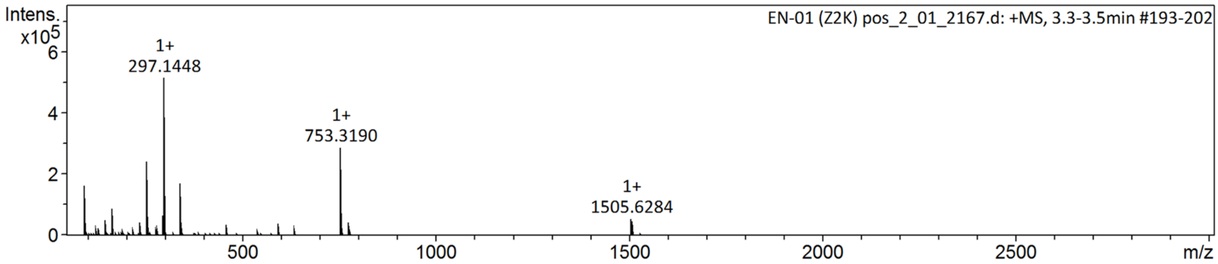

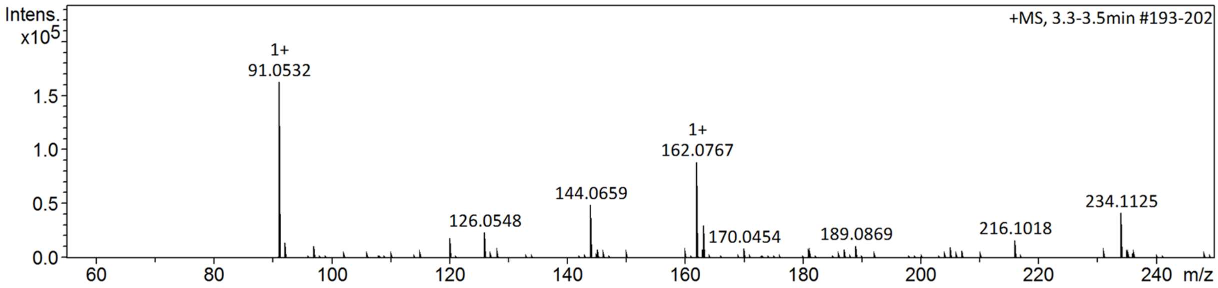

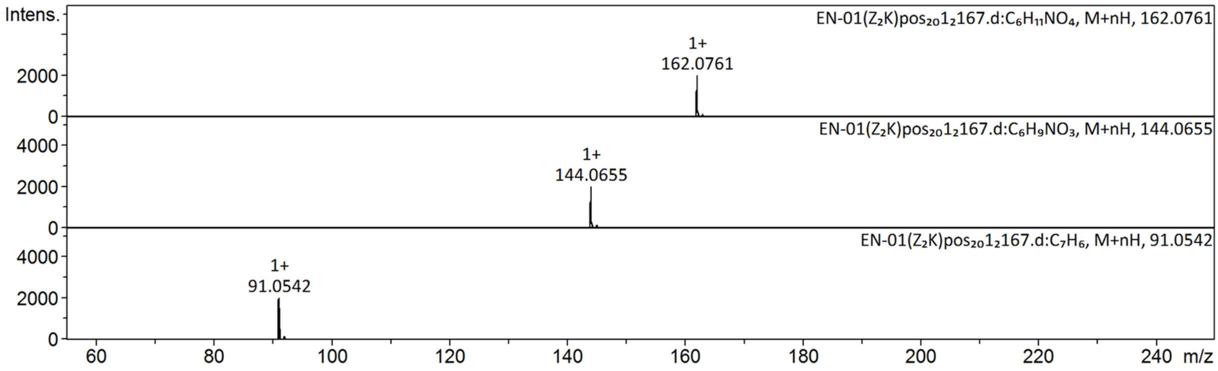

3,6'-Di-Cbz-kanamycinyl A 1-amide of vancomycin (5)

Figure S8. UV-spectrum of 3,6'-di-Cbz-kanamycinyl A 1-amide of vancomycin (**5**)

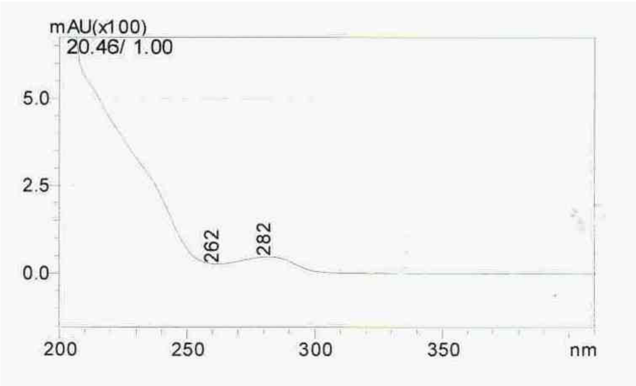

Figure S9. IR spectra of 3,6'-di-Cbz-kanamycinyl A 1-amide of vancomycin (**5**)

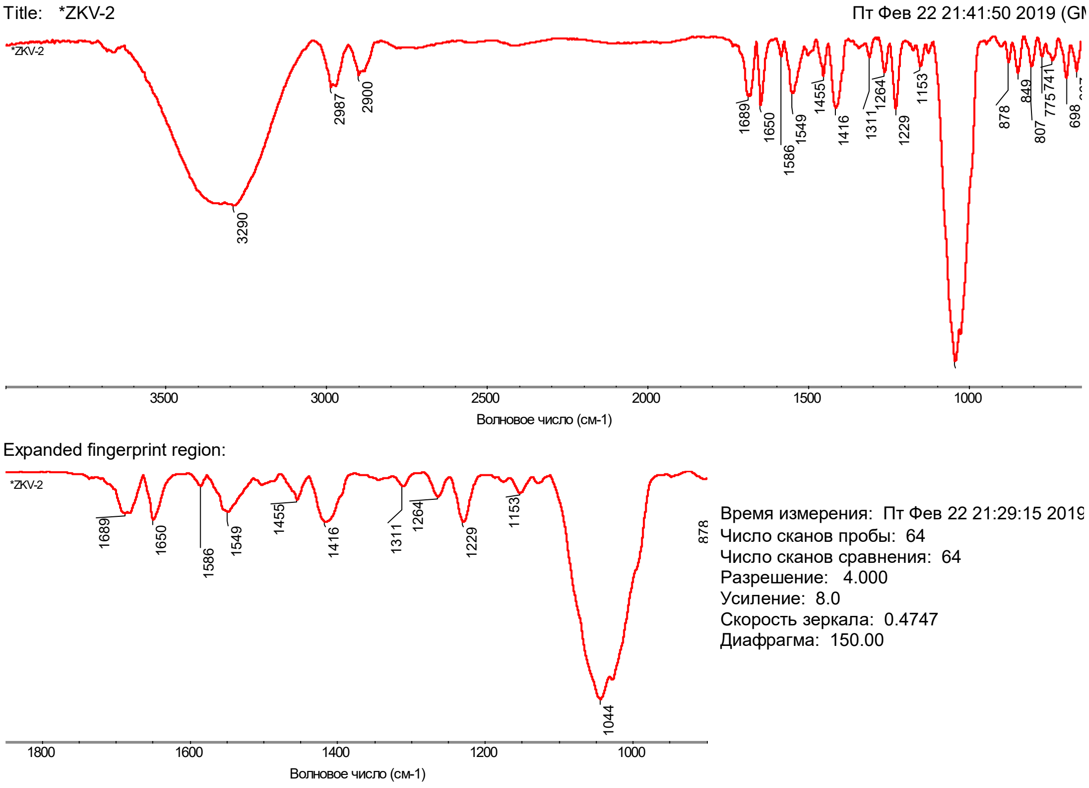

Figure S10. ^{1}H NMR spectrum of 3,6'-di-Cbz-kanamycinyl A 1-amide of vancomycin (**5**)

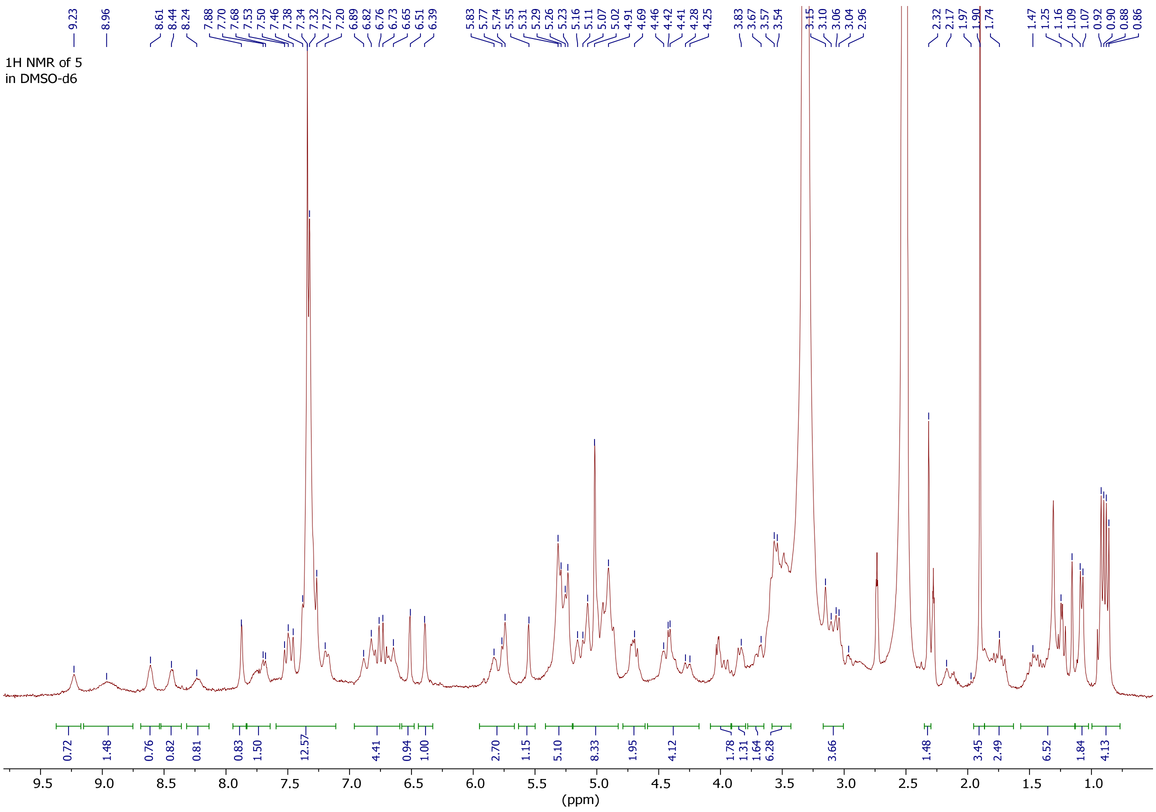

Figure S11. HSQC NMR spectrum of 3,6'-di-Cbz-kanamycinyl A 1-amide of vancomycin (**5**)

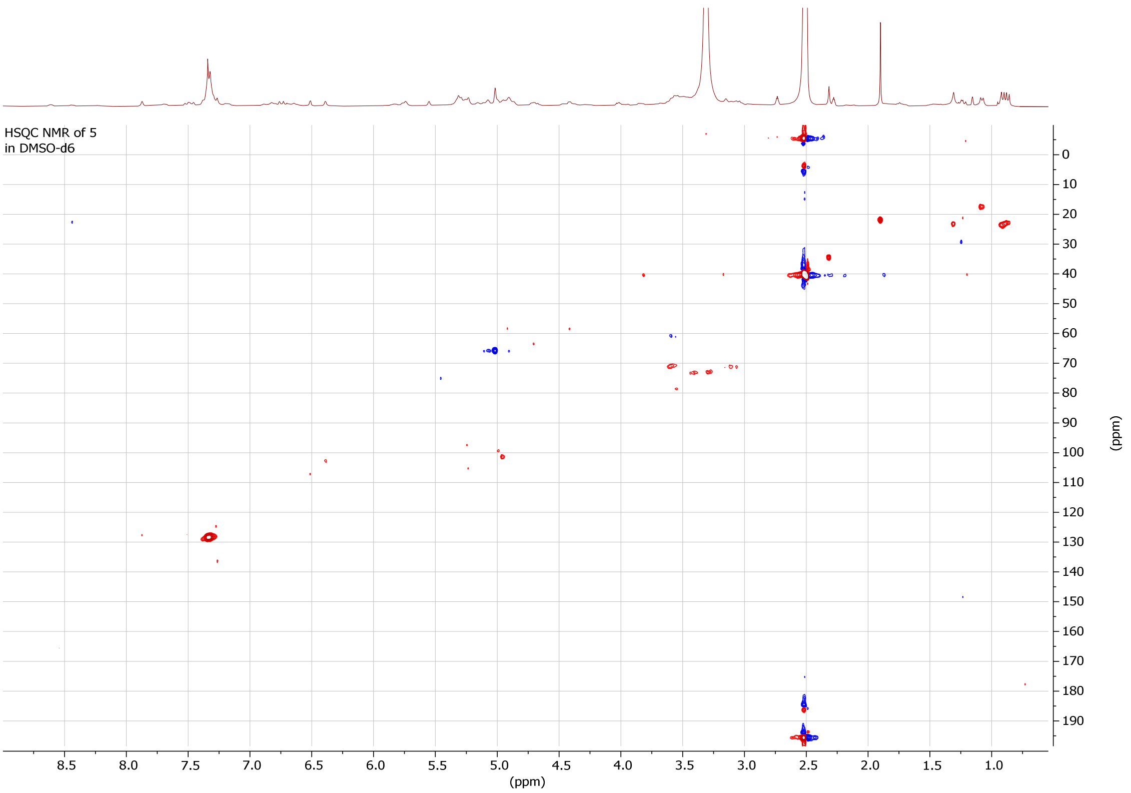

Figure S12. HRMS spectrum of 3,6'-di-Cbz-kanamycinyl A 1-amide of vancomycin (**5**)

Molecular ion $[M+H]^+$ (*m*/*z*, *z*=1):

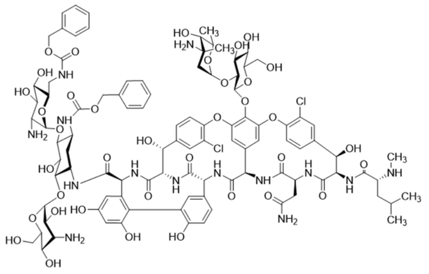

Display Report

Analysis Info

Analysis Name	D:\Data\EN-02 (Z2KV) pos_3_01_2168.d	Acquisition Date	10/22/2018 3:55:22 PM
Method	la-2.2-energy.m	Operator	BDAL@DE
Sample Name	EN-02 (Z2KV) pos	Instrument	compact 8255754.20088
Comment			

Acquisition Parameter

Source Type	ESI	Ion Polarity	Positive	Set Nebulizer	0.4 Bar
Focus	Active	Set Capillary	4500 V	Set Dry Heater	180 °C
Scan Begin	50 m/z	Set End Plate Offset	-500 V	Set Dry Gas	6.0 l/min
Scan End	3000 m/z	Set Charging Voltage	2000 V	Set Divert Valve	Source
		Set Corona	0 nA	Set APCI Heater	0 °C

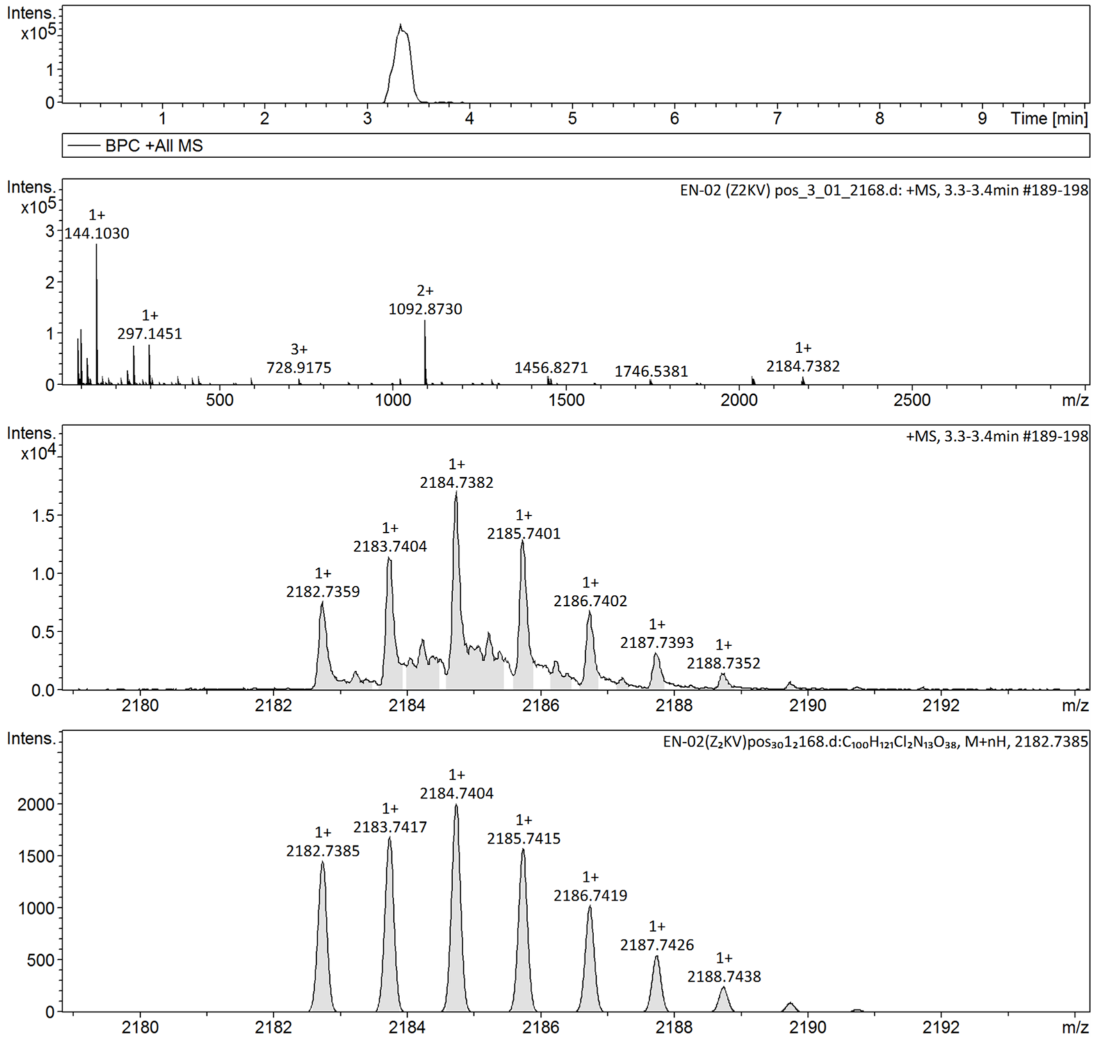

Figure S13. HRMS spectrum of 3,6'-di-Cbz-kanamycinyl A 1-amide of vancomycin (**5**)

Fragmentation ion $[M+H]^+$:

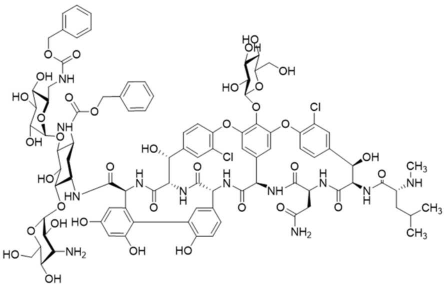

Display Report

Analysis Info

Analysis Name	D:\Data\EN-02 (Z2KV) pos_3_01_2168.d	Acquisition Date	10/22/2018 3:55:22 PM
Method	la-2.2-energy.m	Operator	BDAL@DE
Sample Name	EN-02 (Z2KV) pos	Instrument	compact 8255754.20088
Comment			

Acquisition Parameter

Source Type	ESI	Ion Polarity	Positive	Set Nebulizer	0.4 Bar
Focus	Active	Set Capillary	4500 V	Set Dry Heater	180 °C
Scan Begin	50 m/z	Set End Plate Offset	-500 V	Set Dry Gas	6.0 l/min
Scan End	3000 m/z	Set Charging Voltage	2000 V	Set Divert Valve	Source
		Set Corona	0 nA	Set APCI Heater	0 °C

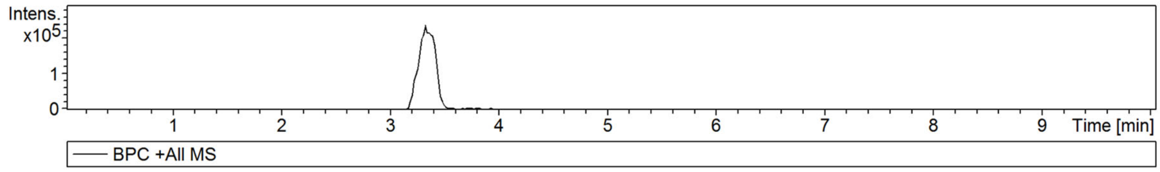

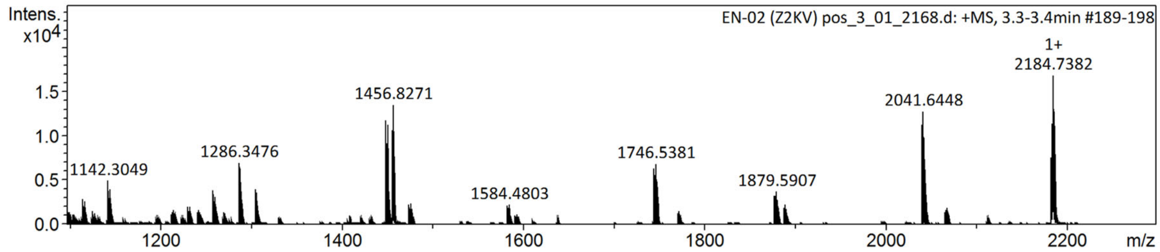

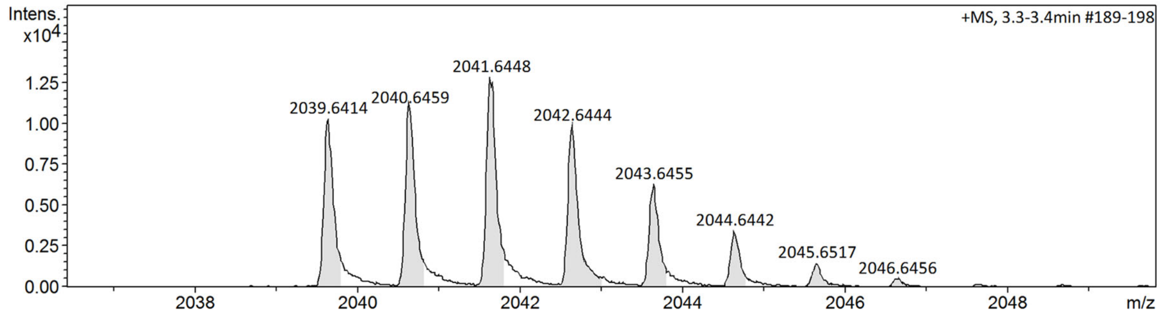

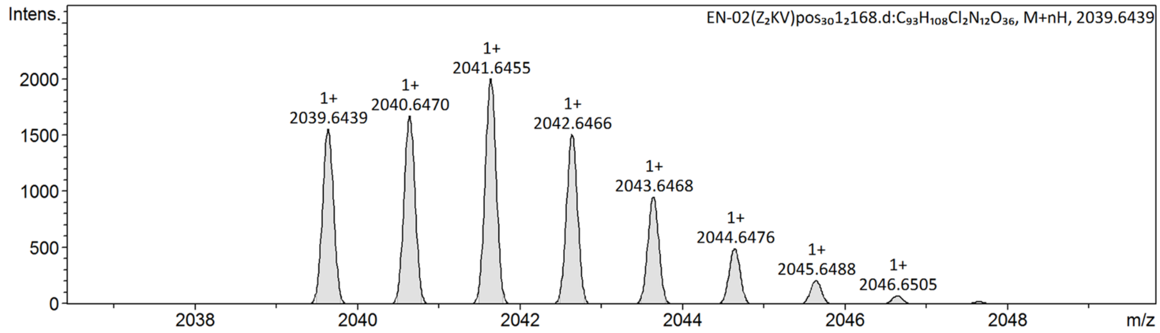

Figure S14. HRMS spectrum of 3,6'-di-Cbz-kanamycinyl A 1-amide of vancomycin (**5**)

Fragmentation ion $[M+H]^{+}$:

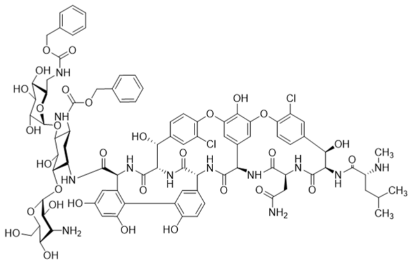

Display Report

Analysis Info

Analysis Name	D:\Data\EN-02 (Z2KV) pos_3_01_2168.d	Acquisition Date	10/22/2018 3:55:22 PM	
Method	la-2.2-energy.m	Operator	BDAL@DE	
Sample Name	EN-02 (Z2KV) pos	Instrument	compact	8255754.20088
Comment				

Acquisition Parameter

Source Type	ESI	Ion Polarity	Positive	Set Nebulizer	0.4 Bar
Focus	Active	Set Capillary	4500 V	Set Dry Heater	180 °C
Scan Begin	50 m/z	Set End Plate Offset	-500 V	Set Dry Gas	6.0 l/min
Scan End	3000 m/z	Set Charging Voltage	2000 V	Set Divert Valve	Source
		Set Corona	0 nA	Set APCI Heater	0 °C

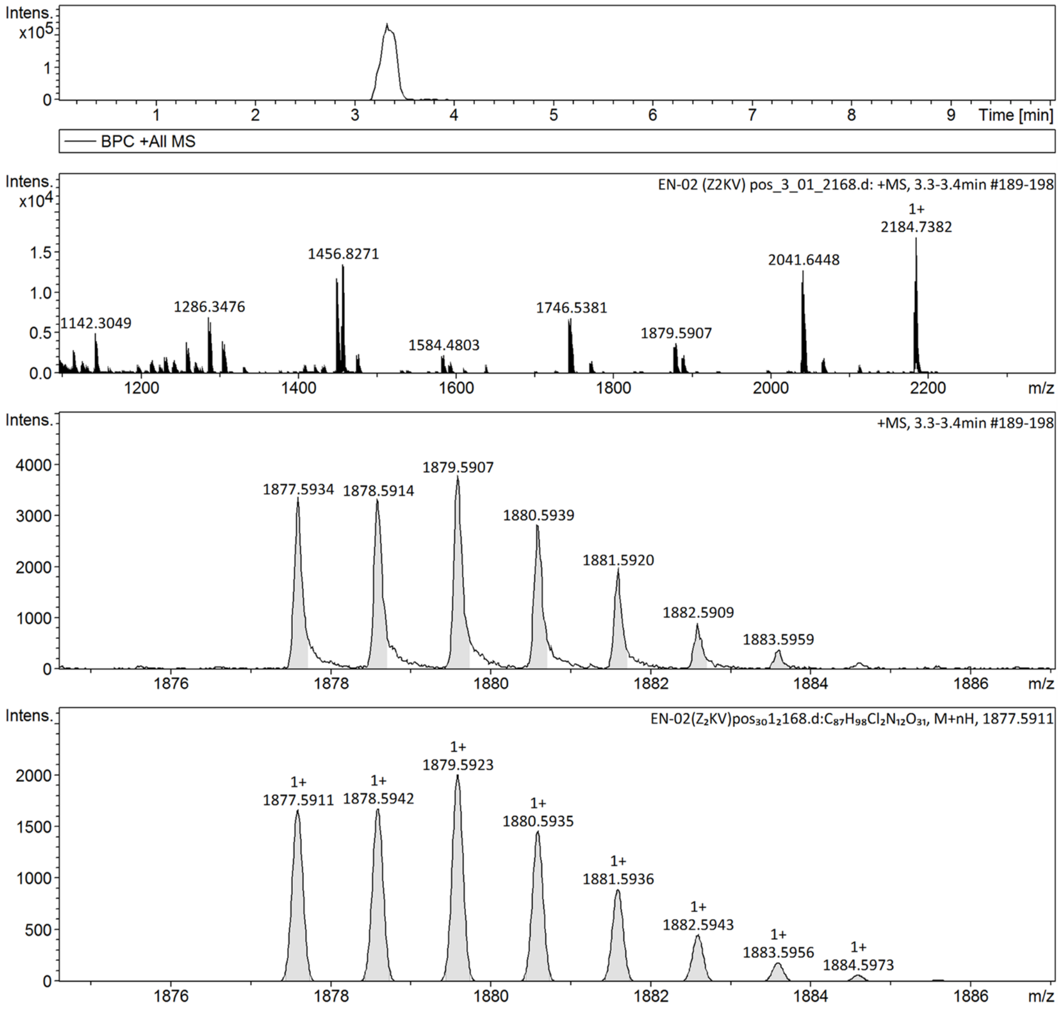

Figure S15. HRMS spectrum of 3,6'-di-Cbz-kanamycinyl A 1-amide of vancomycin (**5**)

Fragmentation ion $[M+H]^+$:

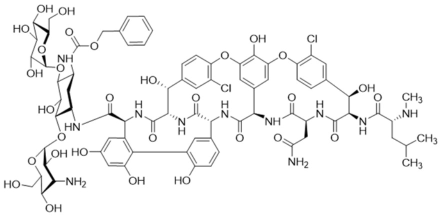

Display Report

Analysis Info

Analysis Name	D:\Data\EN-02 (Z2KV) pos_3_01_2168.d	Acquisition Date	10/22/2018 3:55:22 PM
Method	la-2.2-energy.m	Operator	BDAL@DE
Sample Name	EN-02 (Z2KV) pos	Instrument	compact 8255754.20088
Comment			

Acquisition Parameter

Source Type	ESI	Ion Polarity	Positive	Set Nebulizer	0.4 Bar
Focus	Active	Set Capillary	4500 V	Set Dry Heater	180 °C
Scan Begin	50 m/z	Set End Plate Offset	-500 V	Set Dry Gas	6.0 l/min
Scan End	3000 m/z	Set Charging Voltage	2000 V	Set Divert Valve	Source
		Set Corona	0 nA	Set APCI Heater	0 °C

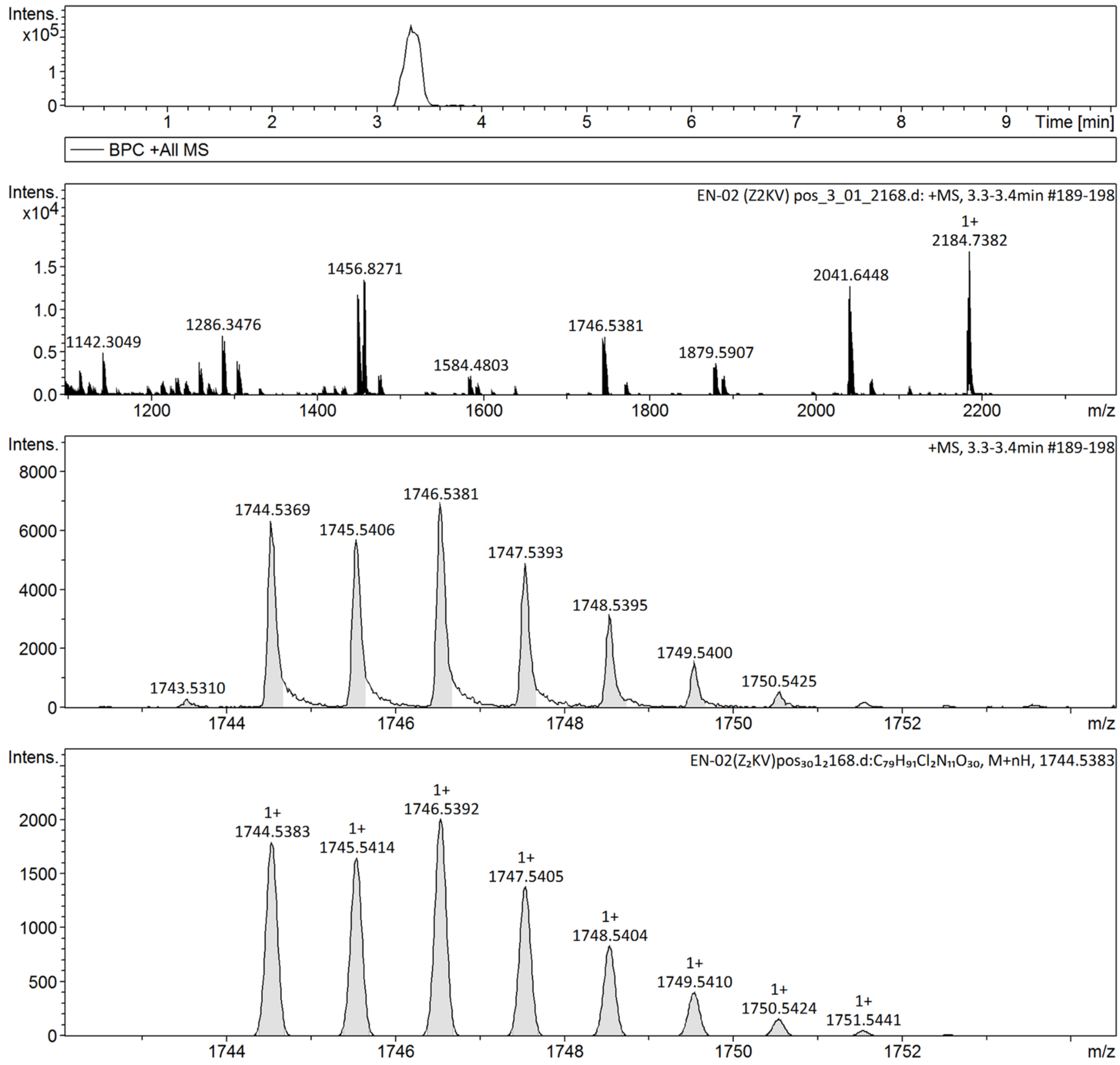

Figure S16. HRMS spectrum of 3,6'-di-Cbz-kanamycinyl A 1-amide of vancomycin (**5**)

Fragmentation ion $[M+H]^+$:

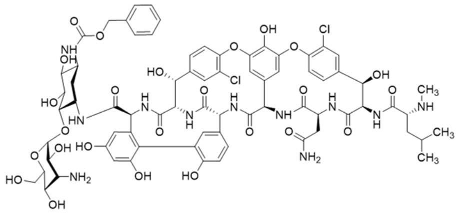

Display Report

Analysis Info

Analysis Name	D:\Data\EN-02 (Z2KV) pos_3_01_2168.d	Acquisition Date	10/22/2018 3:55:22 PM
Method	la-2.2-energy.m	Operator	BDAL@DE
Sample Name	EN-02 (Z2KV) pos	Instrument	compact 8255754.20088
Comment			

Acquisition Parameter

Source Type	ESI	Ion Polarity	Positive	Set Nebulizer	0.4 Bar
Focus	Active	Set Capillary	4500 V	Set Dry Heater	180 °C
Scan Begin	50 m/z	Set End Plate Offset	-500 V	Set Dry Gas	6.0 l/min
Scan End	3000 m/z	Set Charging Voltage	2000 V	Set Divert Valve	Source
		Set Corona	0 nA	Set APCI Heater	0 °C

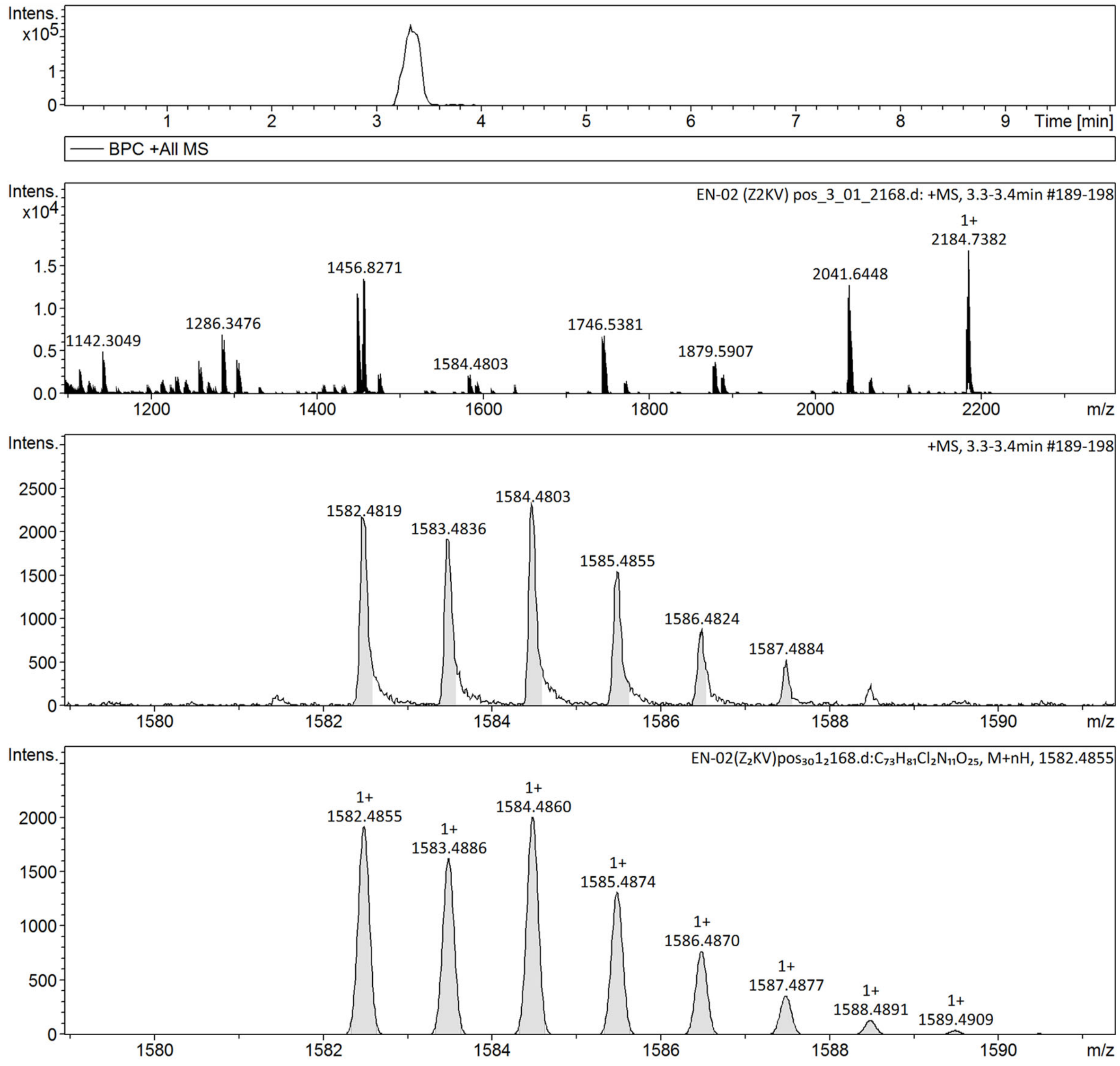

Figure S17. HRMS spectrum of 3,6'-di-Cbz-kanamycinyl A 1-amide of vancomycin (**5**)

Fragmentation ion $[M+H]^+$:

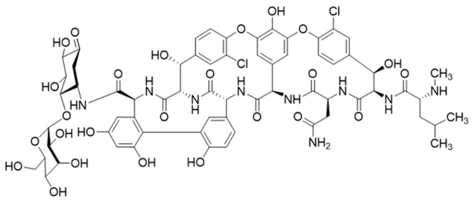

Display Report

Analysis Info

Analysis Name	D:\Data\EN-02 (Z2KV) pos_3_01_2168.d	Acquisition Date	10/22/2018 3:55:22 PM	
Method	la-2.2-energy.m	Operator	BDAL@DE	
Sample Name	EN-02 (Z2KV) pos	Instrument	compact	8255754.20088
Comment				

Acquisition Parameter

Source Type	ESI	Ion Polarity	Positive	Set Nebulizer	0.4 Bar
Focus	Active	Set Capillary	4500 V	Set Dry Heater	180 °C
Scan Begin	50 m/z	Set End Plate Offset	-500 V	Set Dry Gas	6.0 l/min
Scan End	3000 m/z	Set Charging Voltage	2000 V	Set Divert Valve	Source
		Set Corona	0 nA	Set APCI Heater	0 °C

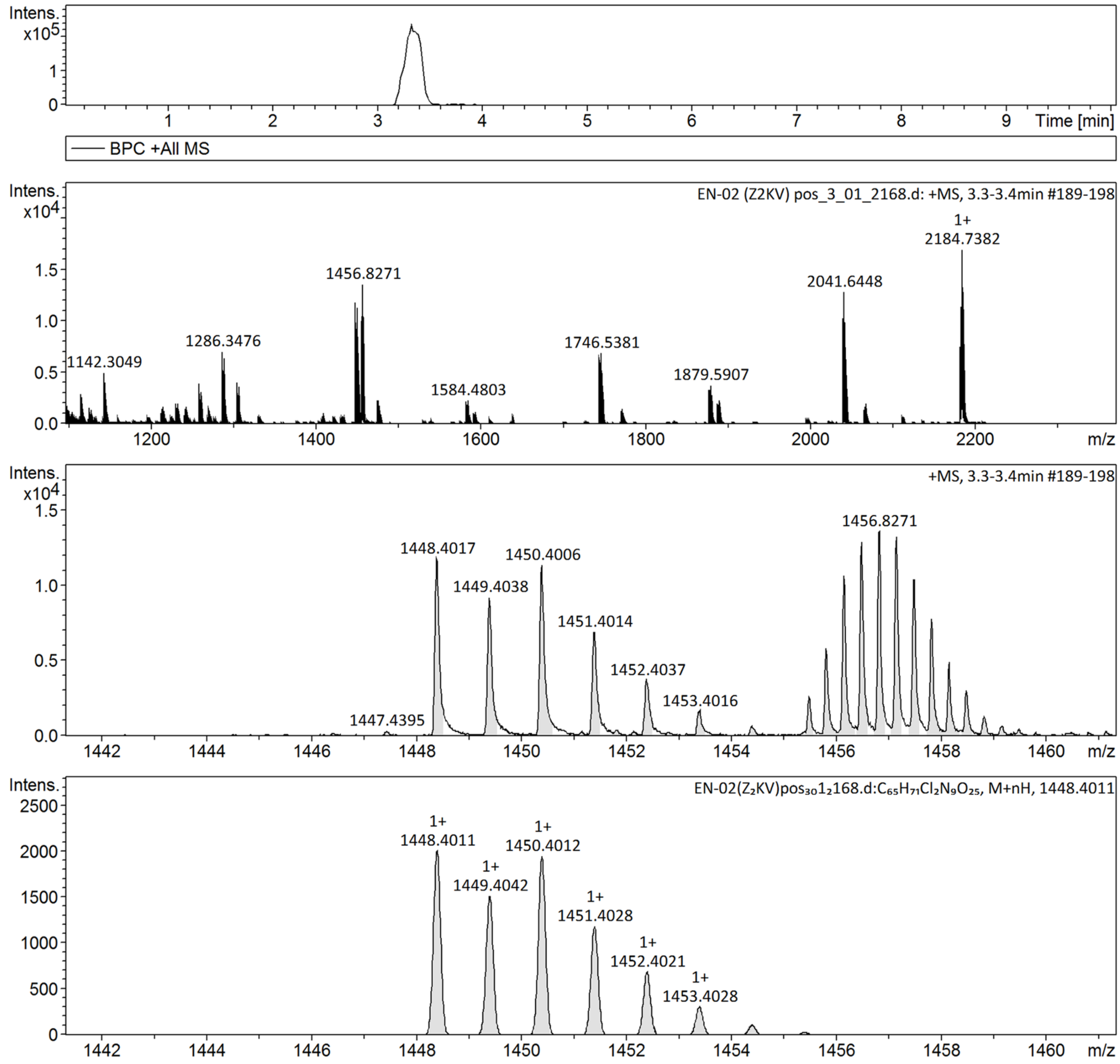

Figure S18. HRMS spectrum of 3,6'-di-Cbz-kanamycinyl A 1-amide of vancomycin (**5**)

Fragmentation ion $[M+H]^+$:

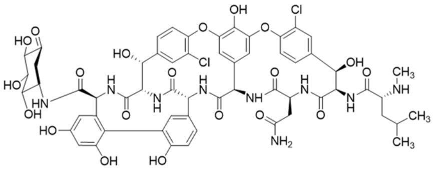

Display Report

Analysis Info

Analysis Name	D:\Data\EN-02 (Z2KV) pos_3_01_2168.d	Acquisition Date	10/22/2018 3:55:22 PM
Method	la-2.2-energy.m	Operator	BDAL@DE
Sample Name	EN-02 (Z2KV) pos	Instrument	compact 8255754.20088
Comment			

Acquisition Parameter

Source Type	ESI	Ion Polarity	Positive	Set Nebulizer	0.4 Bar
Focus	Active	Set Capillary	4500 V	Set Dry Heater	180 °C
Scan Begin	50 m/z	Set End Plate Offset	-500 V	Set Dry Gas	6.0 l/min
Scan End	3000 m/z	Set Charging Voltage	2000 V	Set Divert Valve	Source
		Set Corona	0 nA	Set APCI Heater	0 °C

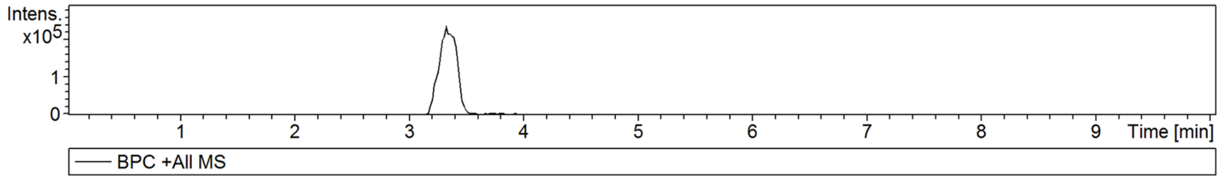

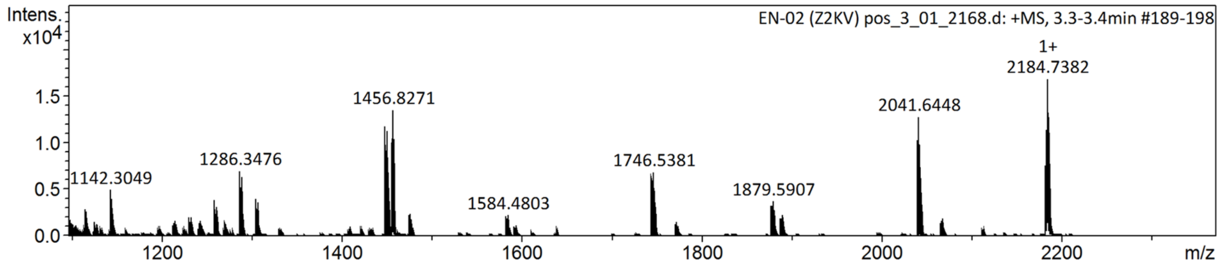

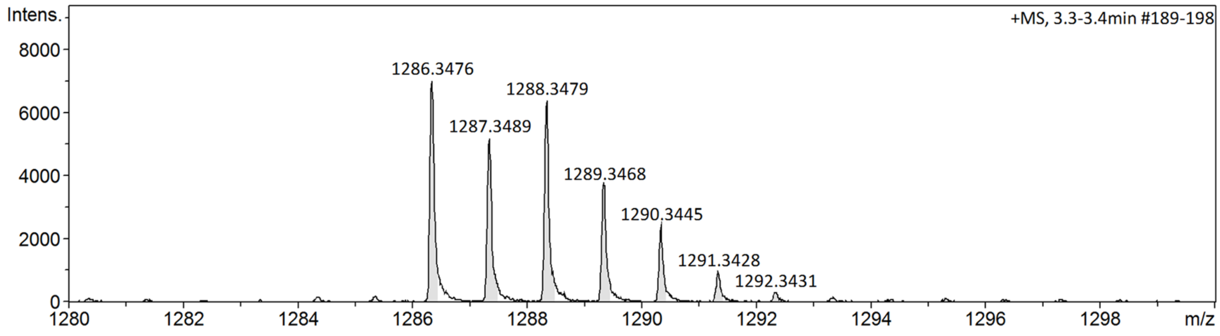

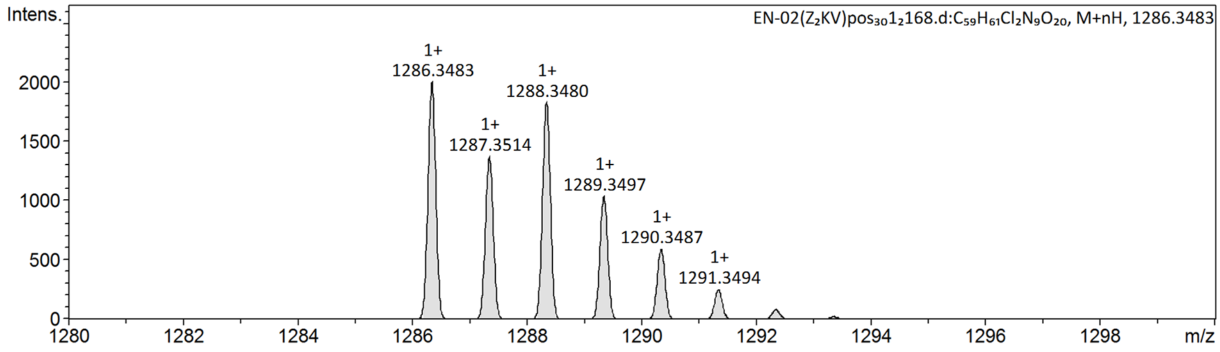

Figure S19. HRMS spectrum of 3,6'-di-Cbz-kanamycinyl A 1-amide of vancomycin (**5**)

Fragmentation ion $[M+H]^+$:

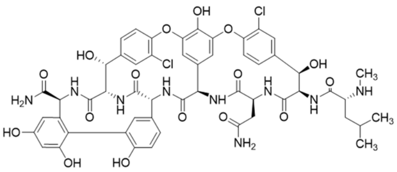

Display Report

Analysis Info

Analysis Name	D:\Data\EN-02 (Z2KV) pos_3_01_2168.d	Acquisition Date	10/22/2018 3:55:22 PM
Method	la-2.2-energy.m	Operator	BDAL@DE
Sample Name	EN-02 (Z2KV) pos	Instrument	compact 8255754.20088
Comment			

Acquisition Parameter

Source Type	ESI	Ion Polarity	Positive	Set Nebulizer	0.4 Bar
Focus	Active	Set Capillary	4500 V	Set Dry Heater	180 °C
Scan Begin	50 m/z	Set End Plate Offset	-500 V	Set Dry Gas	6.0 l/min
Scan End	3000 m/z	Set Charging Voltage	2000 V	Set Divert Valve	Source
		Set Corona	0 nA	Set APCI Heater	0 °C

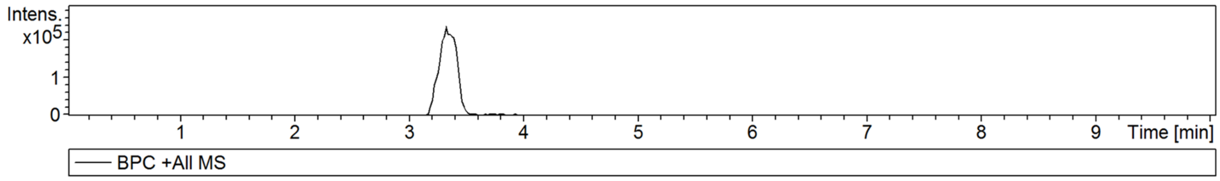

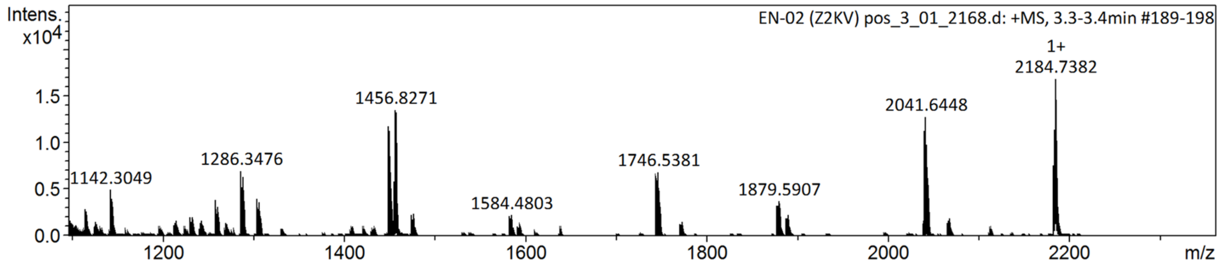

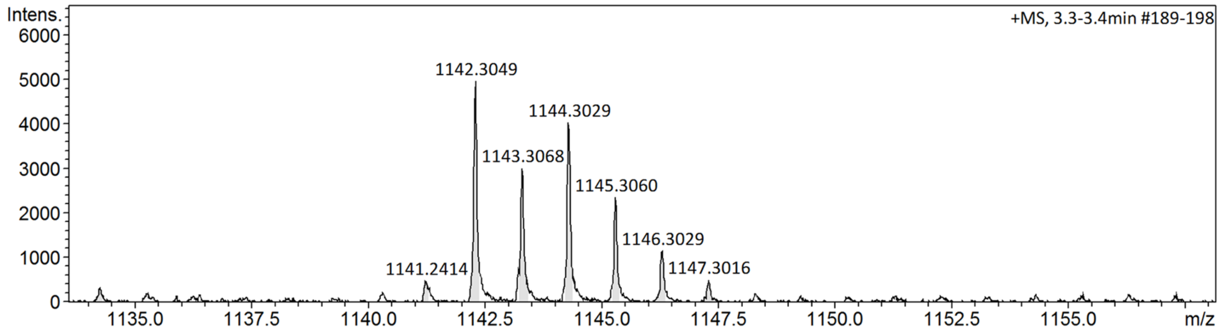

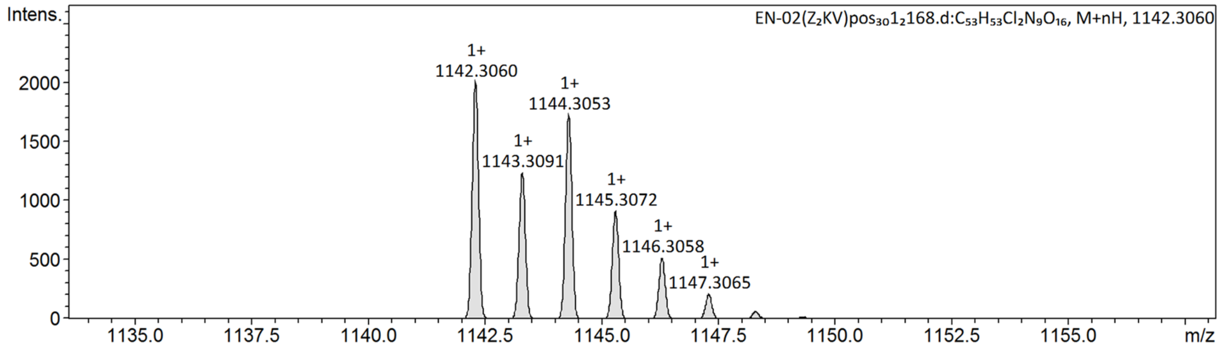

Figure S20. HRMS spectrum of 3,6'-di-Cbz-kanamycinyl A 1-amide of vancomycin (**5**)

Molecular ion $[M+H]^+$ (m/z, z=2):

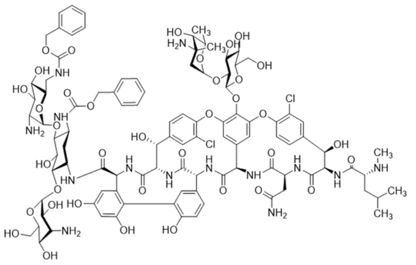

Display Report

Analysis Info

Analysis Name	D:\Data\EN-02 (Z2KV) pos_3_01_2168.d	Acquisition Date	10/22/2018 3:55:22 PM
Method	la-2.2-energy.m	Operator	BDAL@DE
Sample Name	EN-02 (Z2KV) pos	Instrument	compact 8255754.20088
Comment			

Acquisition Parameter

Source Type	ESI	Ion Polarity	Positive	Set Nebulizer	0.4 Bar
Focus	Active	Set Capillary	4500 V	Set Dry Heater	180 °C
Scan Begin	50 m/z	Set End Plate Offset	-500 V	Set Dry Gas	6.0 l/min
Scan End	3000 m/z	Set Charging Voltage	2000 V	Set Divert Valve	Source
		Set Corona	0 nA	Set APCI Heater	0 °C

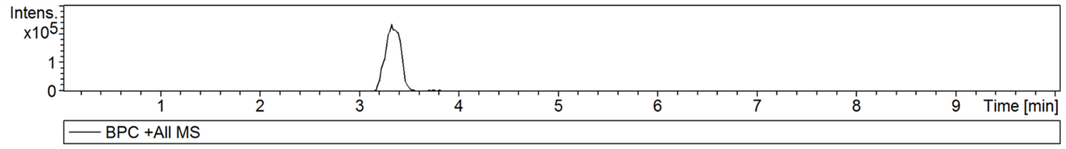

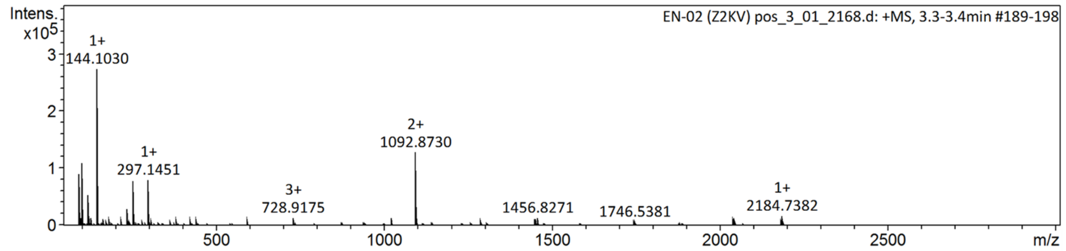

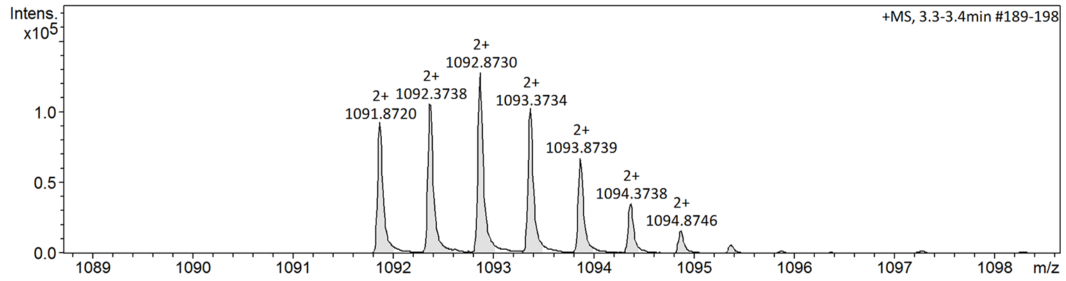

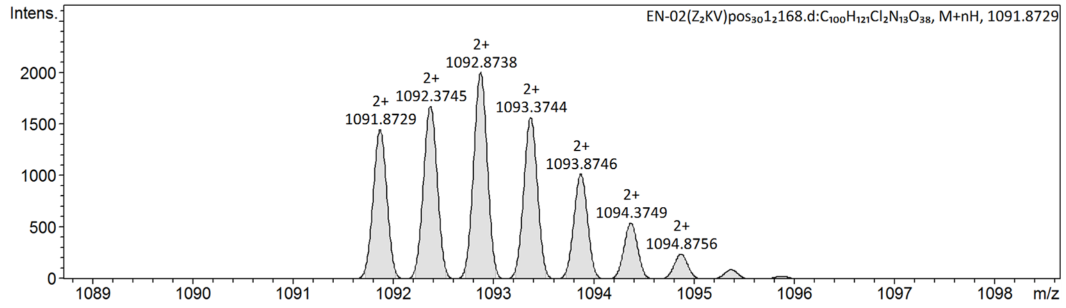

Figure S21. HRMS spectrum of 3,6'-di-Cbz-kanamycinyl A 1-amide of vancomycin (**5**)

Molecular ion [M+H]$^+$ (m/z, z=3):

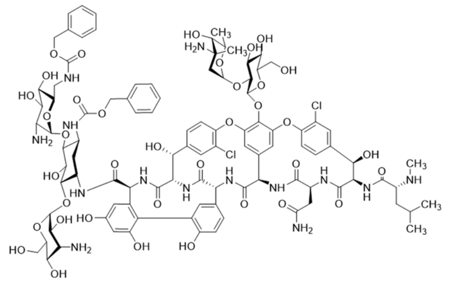

Display Report

Analysis Info

Analysis Name	D:\Data\EN-02 (Z2KV) pos_3_01_2168.d	Acquisition Date	10/22/2018 3:55:22 PM
Method	la-2.2-energy.m	Operator	BDAL@DE
Sample Name	EN-02 (Z2KV) pos	Instrument	compact 8255754.20088
Comment			

Acquisition Parameter

Source Type	ESI	Ion Polarity	Positive	Set Nebulizer	0.4 Bar
Focus	Active	Set Capillary	4500 V	Set Dry Heater	180 °C
Scan Begin	50 m/z	Set End Plate Offset	-500 V	Set Dry Gas	6.0 l/min
Scan End	3000 m/z	Set Charging Voltage	2000 V	Set Divert Valve	Source
		Set Corona	0 nA	Set APCI Heater	0 °C

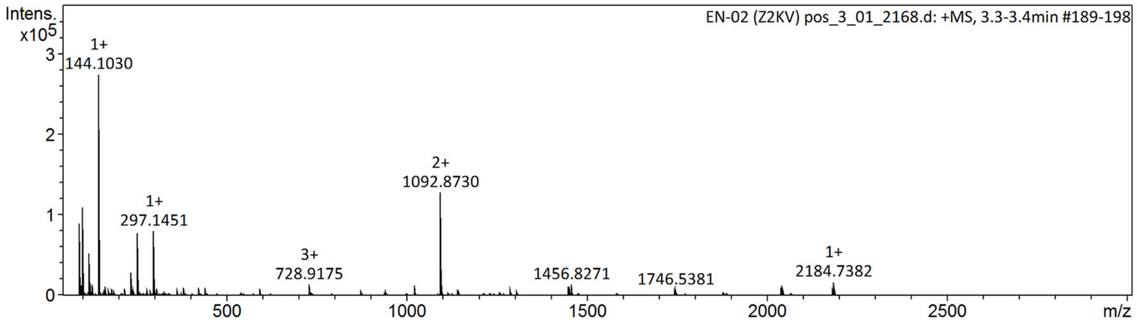

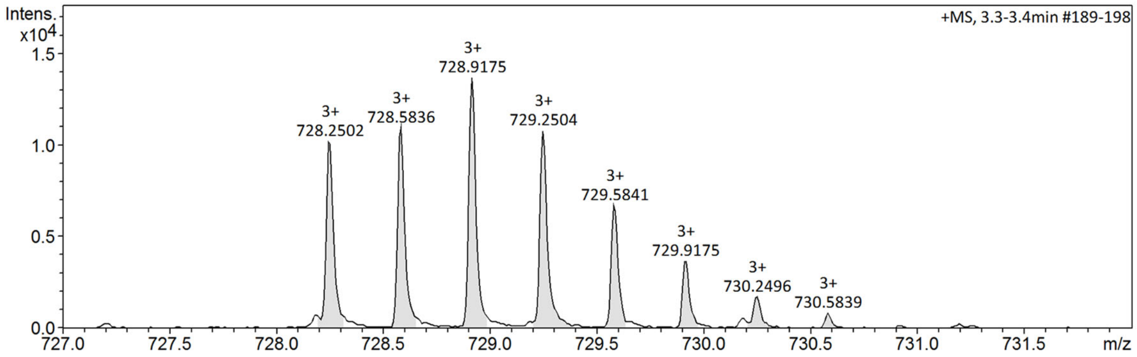

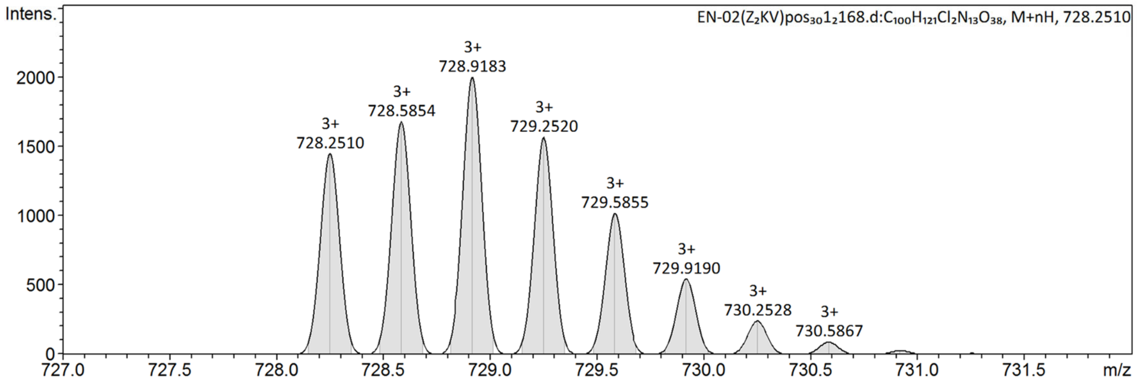

Figure S22. HRMS spectrum of 3,6'-di-Cbz-kanamycinyl A 1-amide of vancomycin (**5**)

Fragmentation ions $[M+H]^+$ and $[tropylium]^+$:

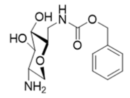

and
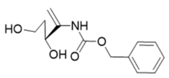

and
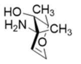

and
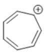

Display Report

Analysis Info

Analysis Name	D:\Data\EN-02 (Z2KV) pos_3_01_2168.d	Acquisition Date	10/22/2018 3:55:22 PM
Method	la-2.2-energy.m	Operator	BDAL@DE
Sample Name	EN-02 (Z2KV) pos	Instrument	compact 8255754.20088
Comment			

Acquisition Parameter

Source Type	ESI	Ion Polarity	Positive	Set Nebulizer	0.4 Bar
Focus	Active	Set Capillary	4500 V	Set Dry Heater	180 °C
Scan Begin	50 m/z	Set End Plate Offset	-500 V	Set Dry Gas	6.0 l/min
Scan End	3000 m/z	Set Charging Voltage	2000 V	Set Divert Valve	Source
		Set Corona	0 nA	Set APCI Heater	0 °C

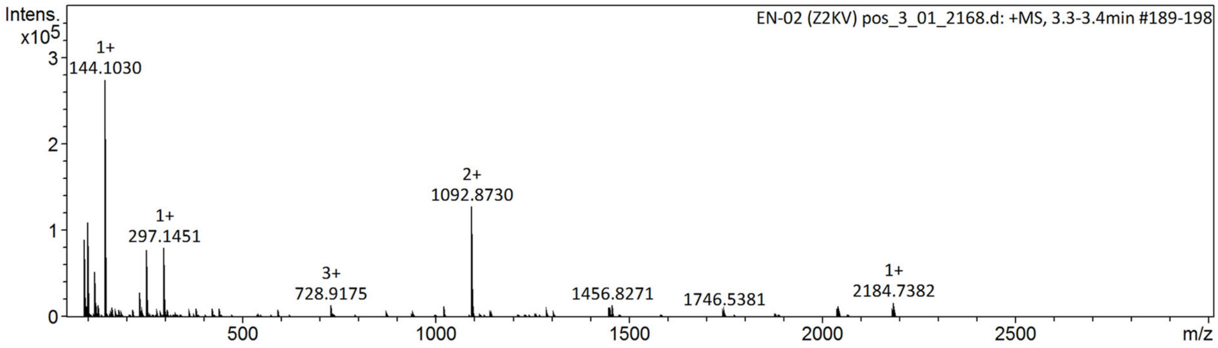

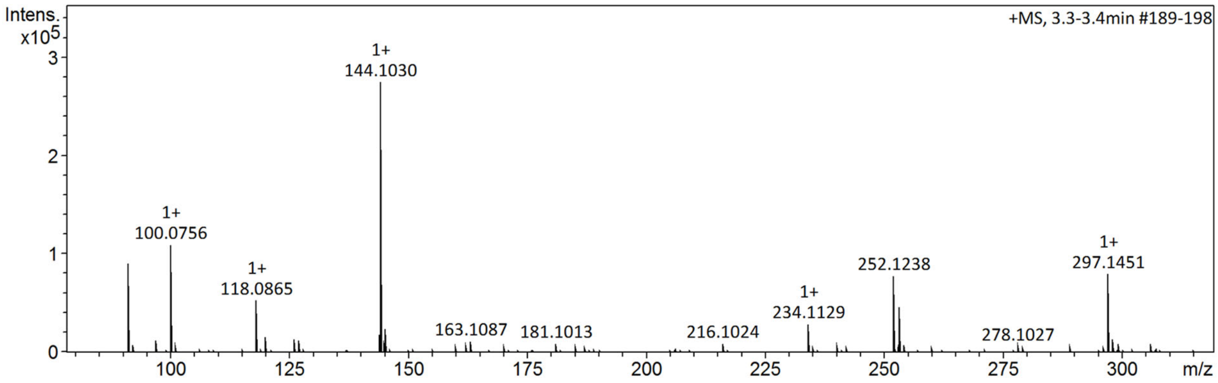

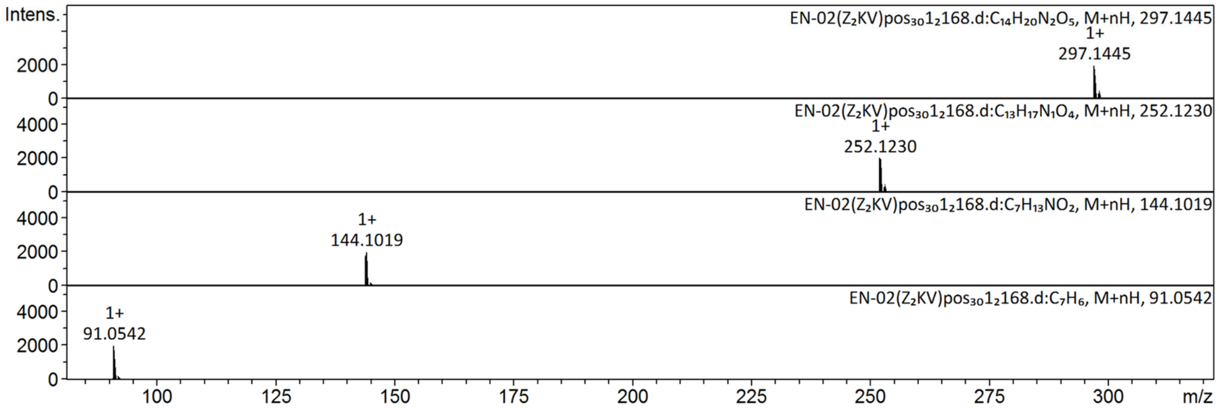

3,6'-Di-Cbz-kanamycinyl A 1-amide of eremomycin (6)

Figure S23. UV-spectrum of 3,6'-di-Cbz-kanamycinyl A 1-amide of eremomycin (**6**)

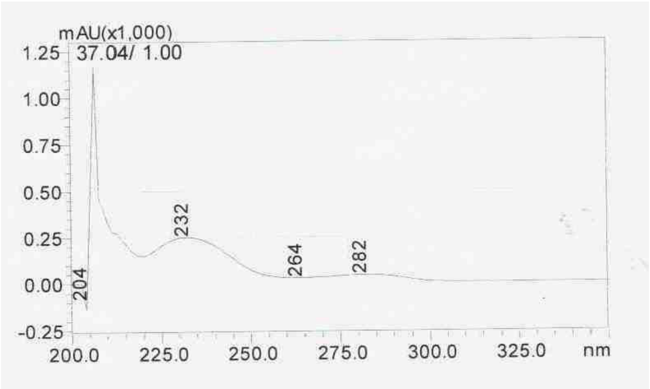

Figure S24. IR spectra of 3,6'-di-Cbz-kanamycinyl A 1-amide of eremomycin (**6**)

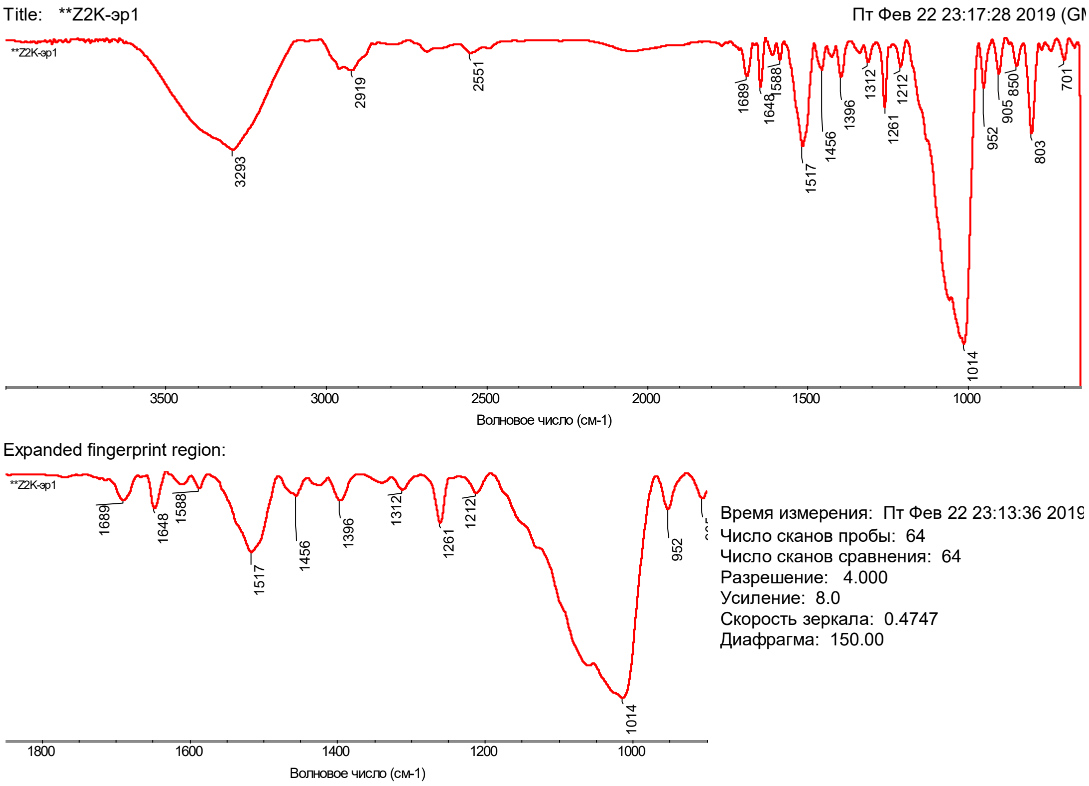

Figure S25. ^{1}H NMR spectrum of 3,6'-di-Cbz-kanamycinyl A 1-amide of eremomycin (**6**)

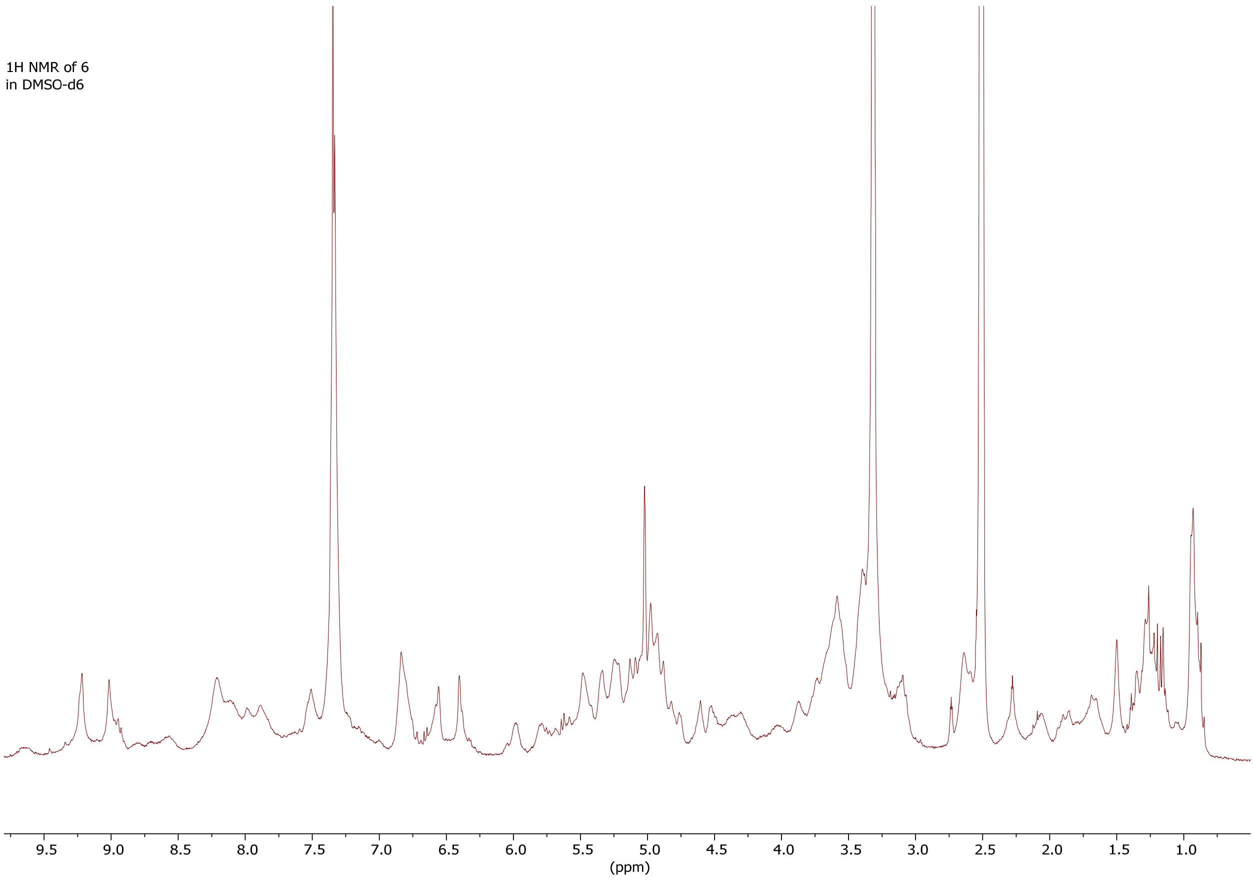

Figure S26. HRMS spectrum of 3,6'-di-Cbz-kanamycinyl A 1-amide of eremomycin (**6**)

Molecular ion $[M+H]^+$ (m/z, z=1):

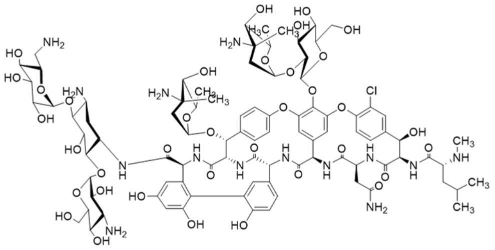

Display Report

Analysis Info

Analysis Name	D:\Data\EN-04 (Z2KE-II) pos_5_01_2170.d	Acquisition Date	10/22/2018 4:18:46 PM
Method	la-2.2-energy.m	Operator	BDAL@DE
Sample Name	EN-04 (Z2KE-II) pos	Instrument	compact 8255754.20088
Comment			

Acquisition Parameter

Source Type	ESI	Ion Polarity	Positive	Set Nebulizer	0.4 Bar
Focus	Active	Set Capillary	4500 V	Set Dry Heater	180 °C
Scan Begin	50 m/z	Set End Plate Offset	-500 V	Set Dry Gas	6.0 l/min
Scan End	3000 m/z	Set Charging Voltage	2000 V	Set Divert Valve	Source
		Set Corona	0 nA	Set APCI Heater	0 °C

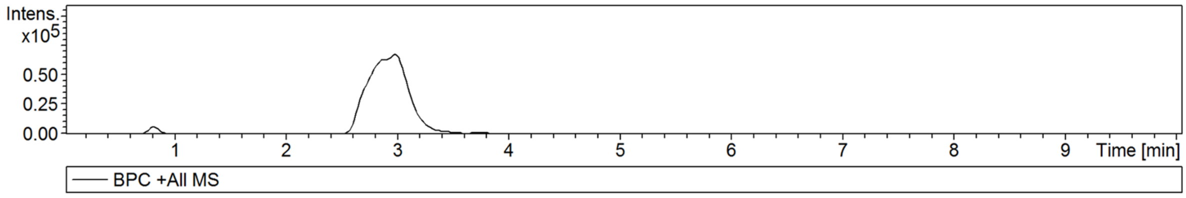

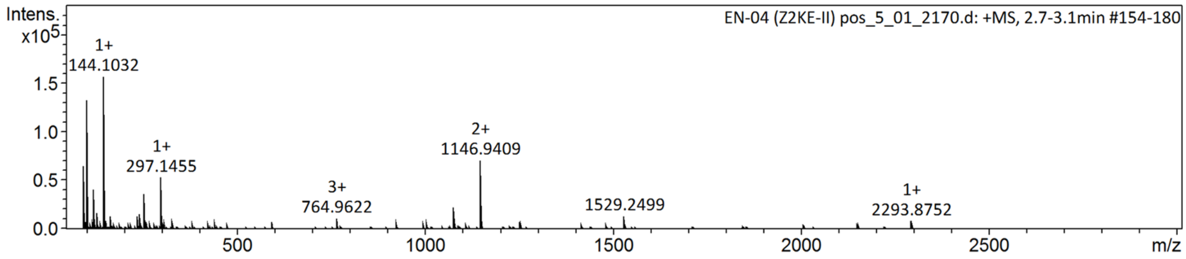

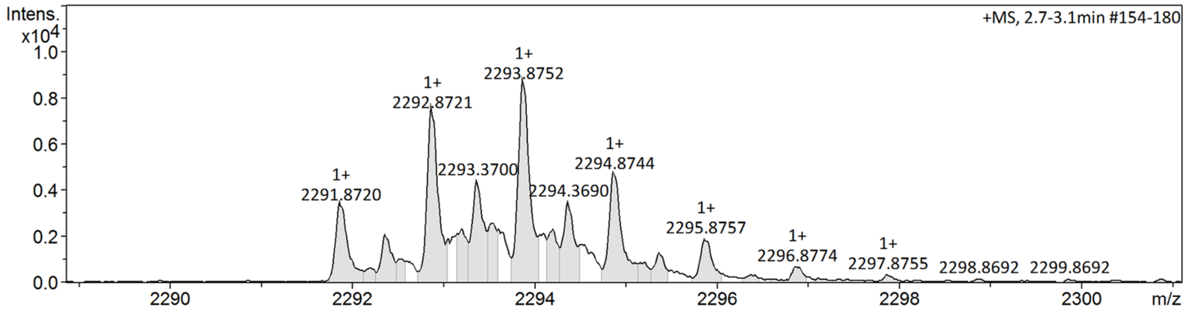

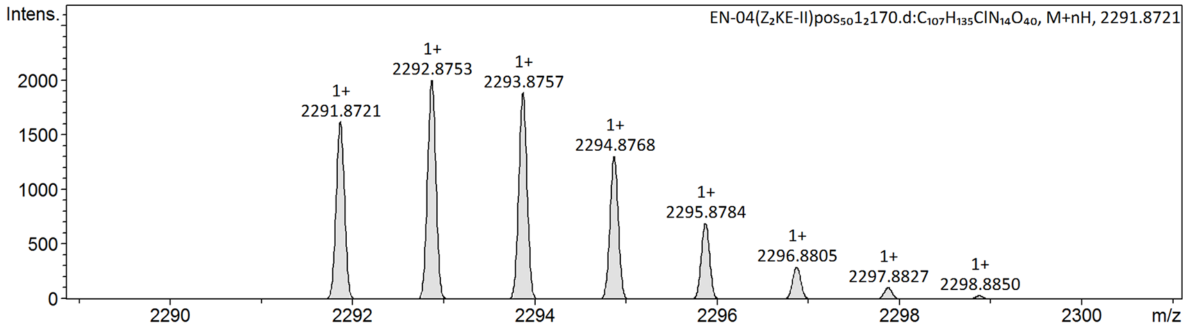

Figure S27. HRMS spectrum of 3,6'-di-Cbz-kanamycinyl A 1-amide of eremomycin (**6**)

Fragmentation ion $[M+H]^+$:

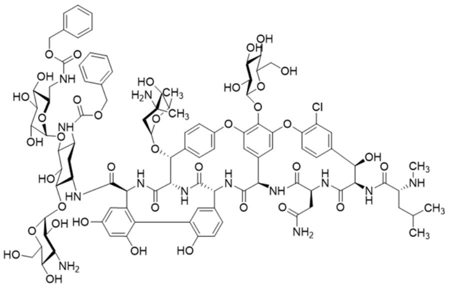

Display Report

Analysis Info

Analysis Name	D:\Data\EN-04 (Z2KE-II) pos_5_01_2170.d	Acquisition Date	10/22/2018 4:18:46 PM
Method	la-2.2-energy.m	Operator	BDAL@DE
Sample Name	EN-04 (Z2KE-II) pos	Instrument	compact 8255754.20088
Comment			

Acquisition Parameter

Source Type	ESI	Ion Polarity	Positive	Set Nebulizer	0.4 Bar
Focus	Active	Set Capillary	4500 V	Set Dry Heater	180 °C
Scan Begin	50 m/z	Set End Plate Offset	-500 V	Set Dry Gas	6.0 l/min
Scan End	3000 m/z	Set Charging Voltage	2000 V	Set Divert Valve	Source
		Set Corona	0 nA	Set APCI Heater	0 °C

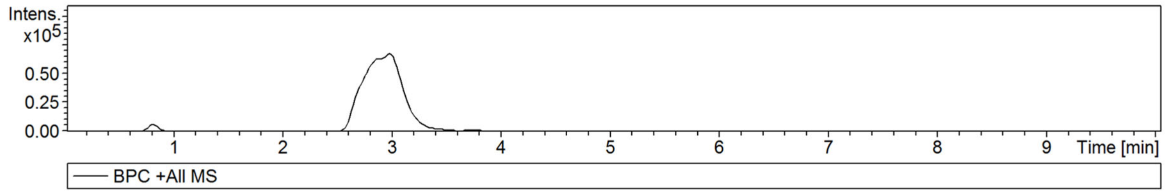

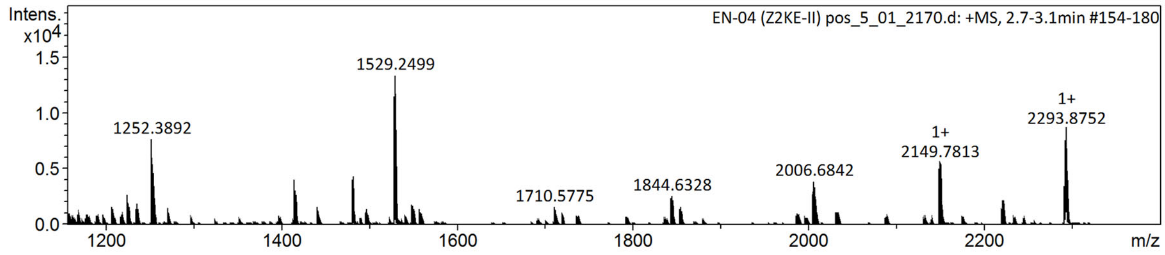

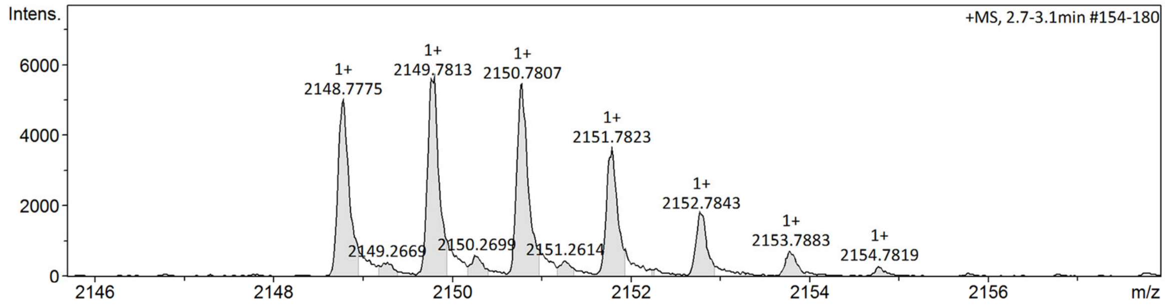

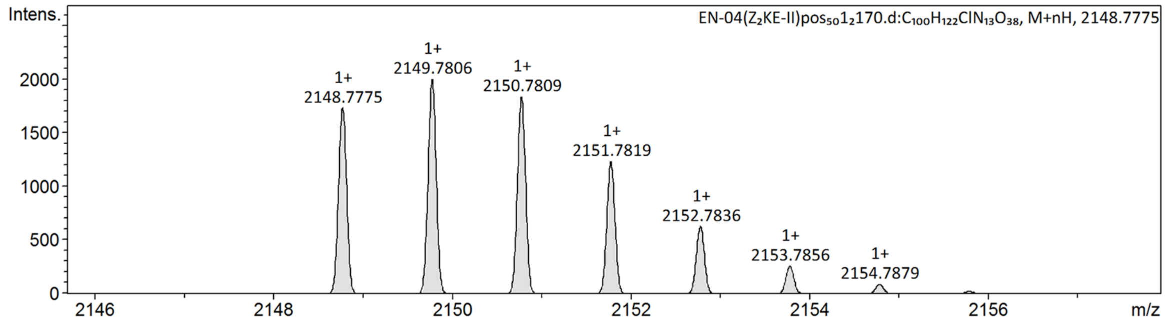

Figure S28. HRMS spectrum of 3,6'-di-Cbz-kanamycinyl A 1-amide of eremomycin (**6**)

Fragmentation ion $[M+H]^+$:

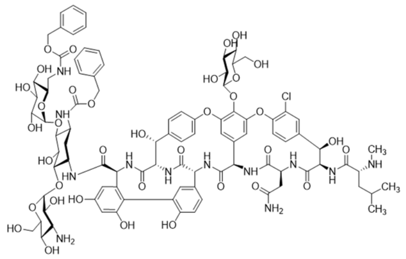

Display Report

Analysis Info

Analysis Name	D:\Data\EN-04 (Z2KE-II) pos_5_01_2170.d	Acquisition Date	10/22/2018 4:18:46 PM
Method	la-2.2-energy.m	Operator	BDAL@DE
Sample Name	EN-04 (Z2KE-II) pos	Instrument	compact 8255754.20088
Comment			

Acquisition Parameter

Source Type	ESI	Ion Polarity	Positive	Set Nebulizer	0.4 Bar
Focus	Active	Set Capillary	4500 V	Set Dry Heater	180 °C
Scan Begin	50 m/z	Set End Plate Offset	-500 V	Set Dry Gas	6.0 l/min
Scan End	3000 m/z	Set Charging Voltage	2000 V	Set Divert Valve	Source
		Set Corona	0 nA	Set APCI Heater	0 °C

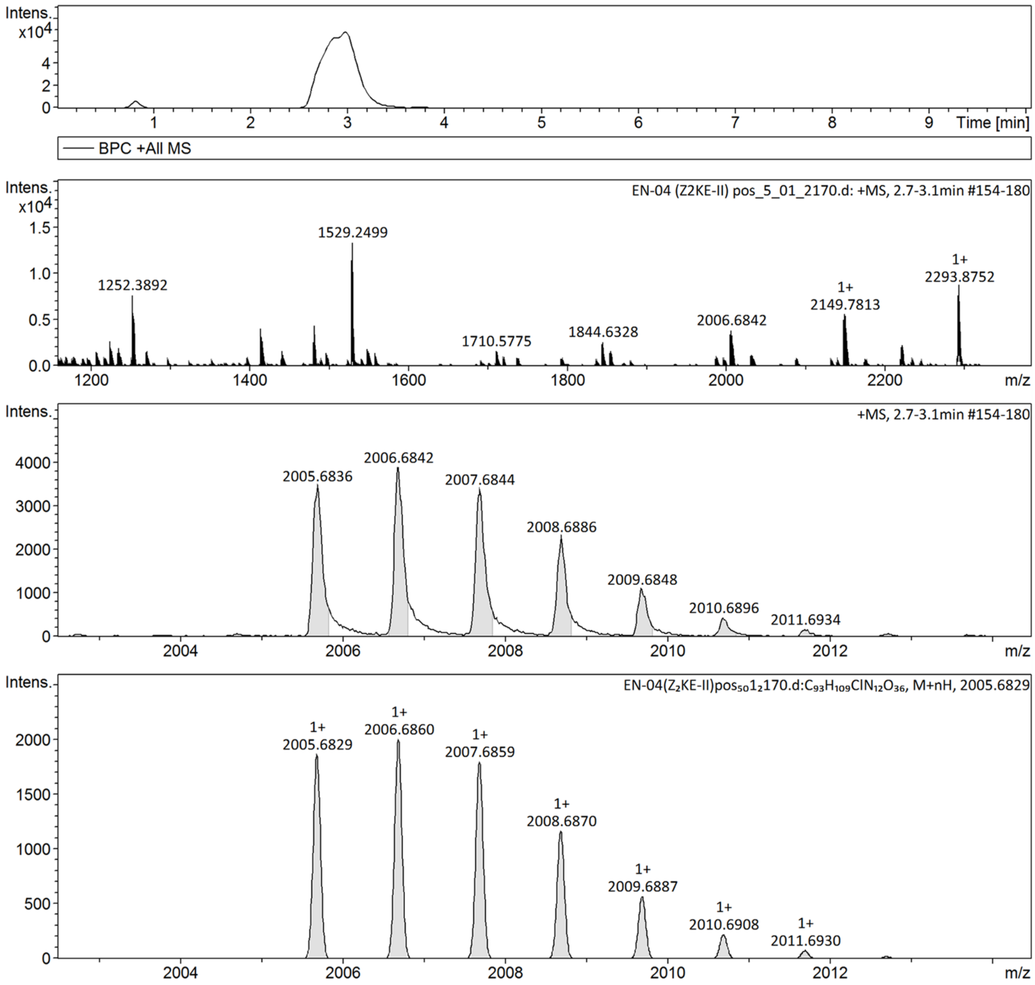

Figure S29. HRMS spectrum of 3,6'-di-Cbz-kanamycinyl A 1-amide of eremomycin (**6**)

Fragmentation ion [M+H]+:

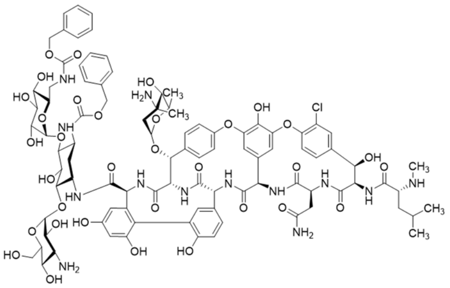

Display Report

Analysis Info

Analysis Name	D:\Data\EN-04 (Z2KE-II) pos_5_01_2170.d	Acquisition Date	10/22/2018 4:18:46 PM
Method	la-2.2-energy.m	Operator	BDAL@DE
Sample Name	EN-04 (Z2KE-II) pos	Instrument	compact 8255754.20088
Comment			

Acquisition Parameter

Source Type	ESI	Ion Polarity	Positive	Set Nebulizer	0.4 Bar
Focus	Active	Set Capillary	4500 V	Set Dry Heater	180 °C
Scan Begin	50 m/z	Set End Plate Offset	-500 V	Set Dry Gas	6.0 l/min
Scan End	3000 m/z	Set Charging Voltage	2000 V	Set Divert Valve	Source
		Set Corona	0 nA	Set APCI Heater	0 °C

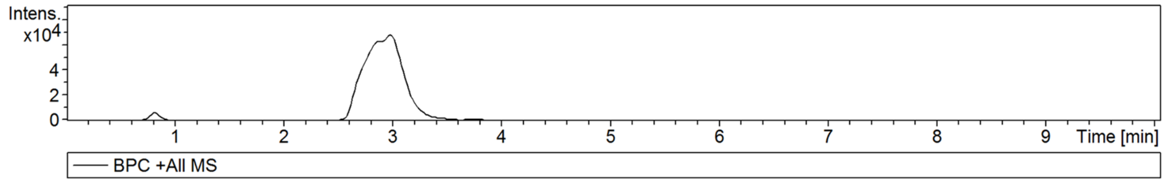

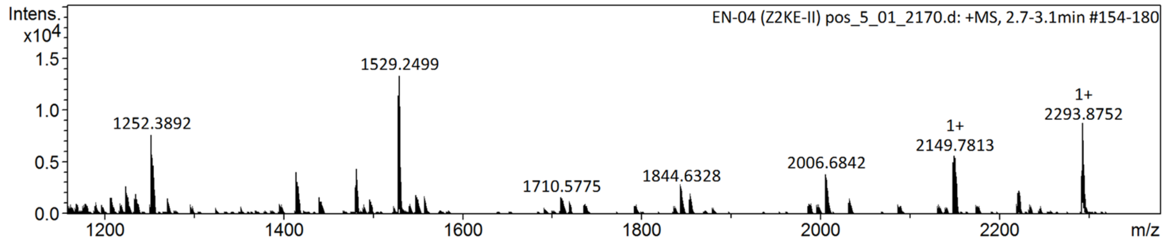

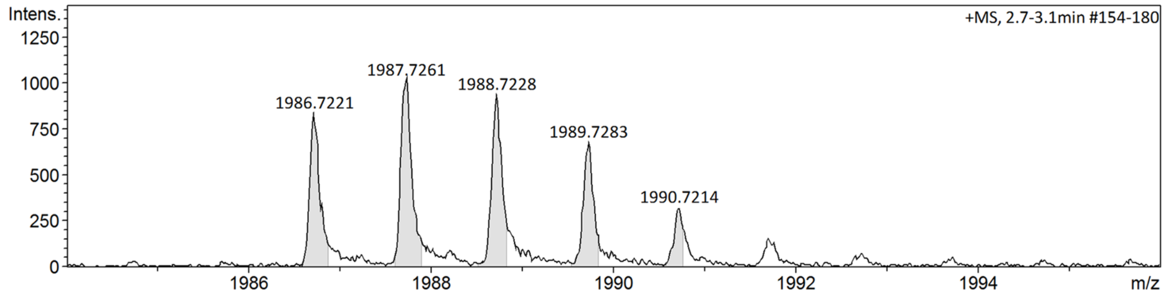

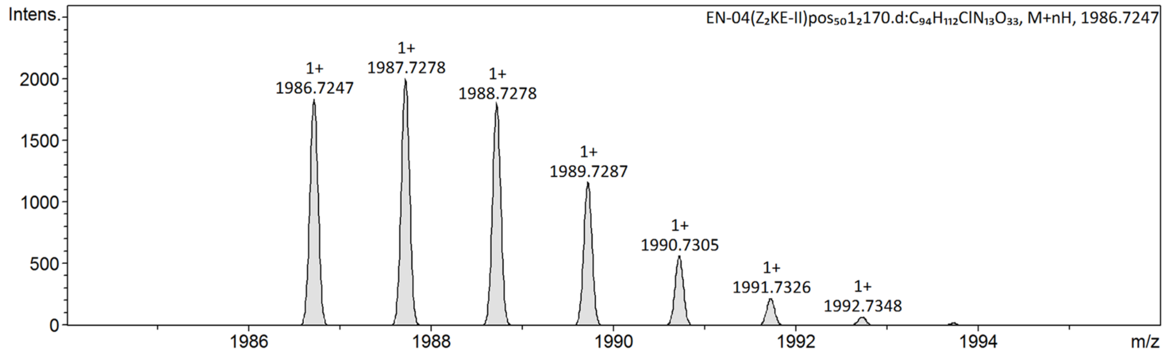

`Figure S30. HRMS spectrum of 3,6'-di-Cbz-kanamycinyl A 1-amide of eremomycin (**6**)

Fragmentation ion [M+H]+:

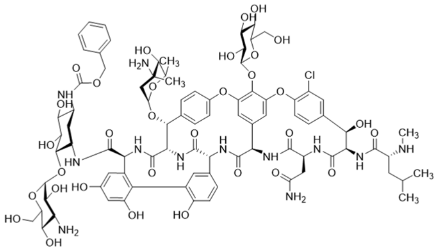

Display Report

Analysis Info

Analysis Name	D:\Data\EN-04 (Z2KE-II) pos_5_01_2170.d	Acquisition Date	10/22/2018 4:18:46 PM
Method	la-2.2-energy.m	Operator	BDAL@DE
Sample Name	EN-04 (Z2KE-II) pos	Instrument	compact 8255754.20088
Comment			

Acquisition Parameter

Source Type	ESI	Ion Polarity	Positive	Set Nebulizer	0.4 Bar
Focus	Active	Set Capillary	4500 V	Set Dry Heater	180 °C
Scan Begin	50 m/z	Set End Plate Offset	-500 V	Set Dry Gas	6.0 l/min
Scan End	3000 m/z	Set Charging Voltage	2000 V	Set Divert Valve	Source
		Set Corona	0 nA	Set APCI Heater	0 °C

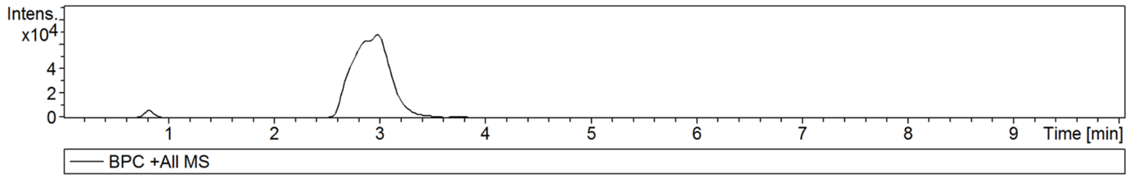

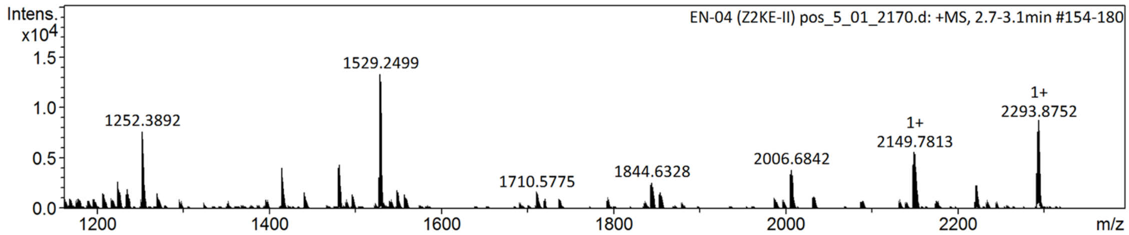

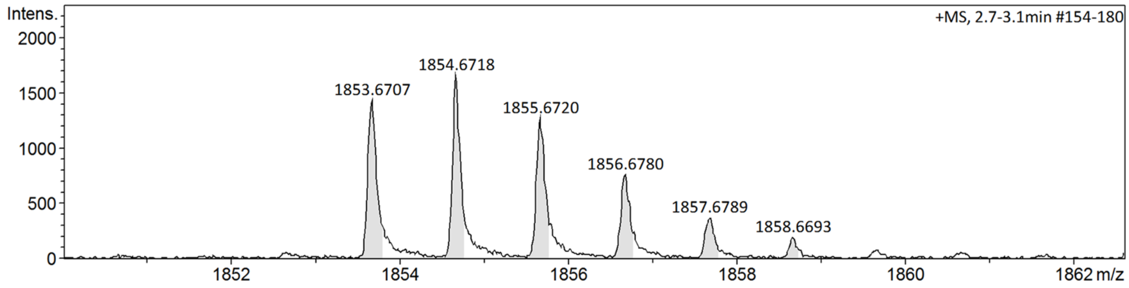

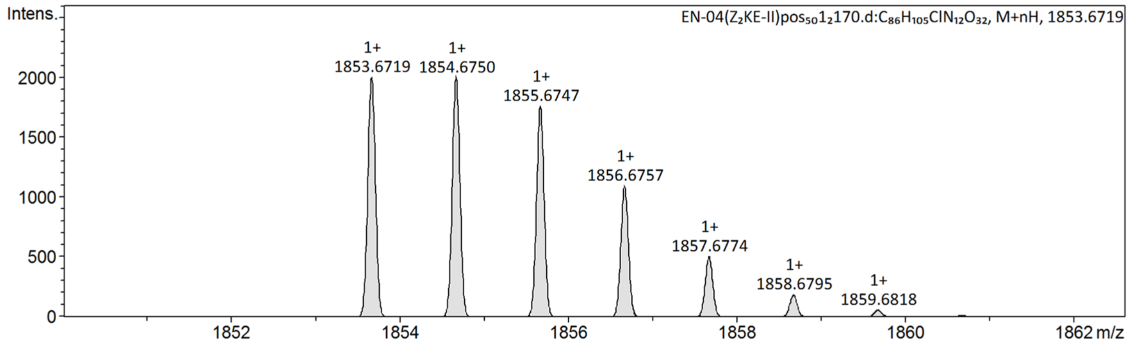

Figure S31. HRMS spectrum of 3,6'-di-Cbz-kanamycinyl A 1-amide of eremomycin (**6**)

Fragmentation ion $[M+H]^+$:

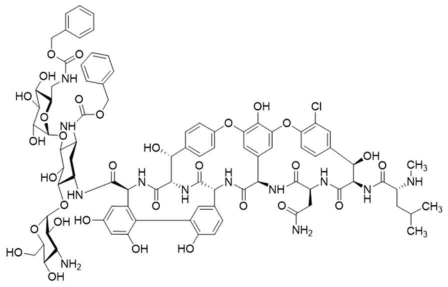

Display Report

Analysis Info

Analysis Name	D:\Data\EN-04 (Z2KE-II) pos_5_01_2170.d	Acquisition Date	10/22/2018 4:18:46 PM
Method	la-2.2-energy.m	Operator	BDAL@DE
Sample Name	EN-04 (Z2KE-II) pos	Instrument	compact 8255754.20088
Comment			

Acquisition Parameter

Source Type	ESI	Ion Polarity	Positive	Set Nebulizer	0.4 Bar
Focus	Active	Set Capillary	4500 V	Set Dry Heater	180 °C
Scan Begin	50 m/z	Set End Plate Offset	-500 V	Set Dry Gas	6.0 l/min
Scan End	3000 m/z	Set Charging Voltage	2000 V	Set Divert Valve	Source
		Set Corona	0 nA	Set APCI Heater	0 °C

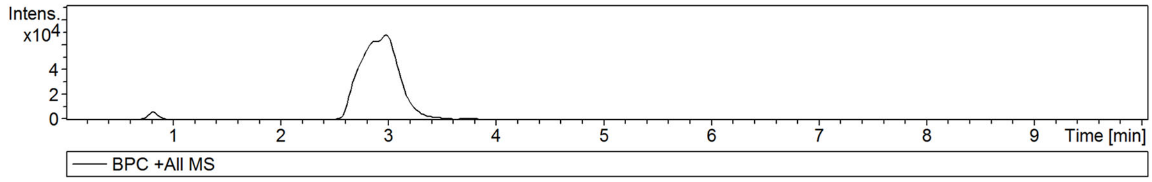

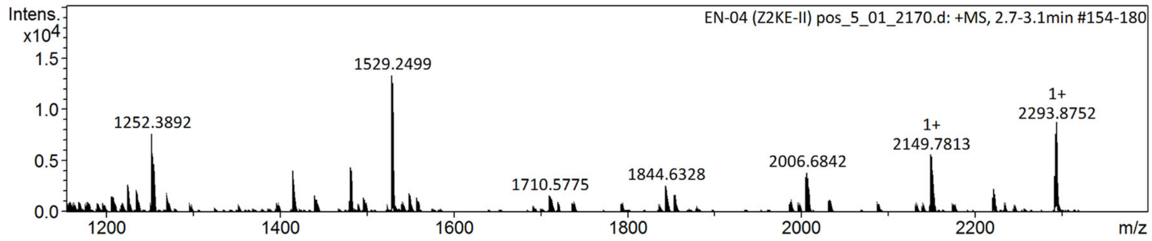

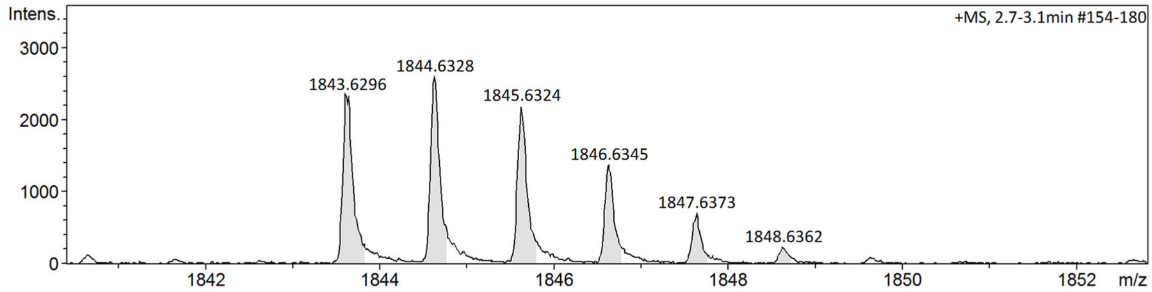

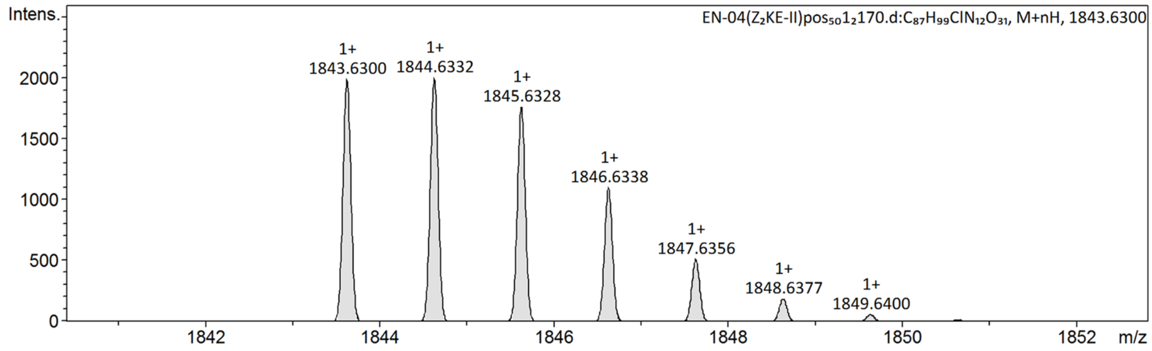

Figure S32. HRMS spectrum of 3,6'-di-Cbz-kanamycinyl A 1-amide of eremomycin (**6**)

Fragmentation ion [M+H]+:

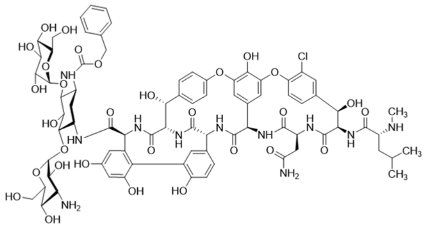

Display Report

Analysis Info

Analysis Name	D:\Data\EN-04 (Z2KE-II) pos_5_01_2170.d	Acquisition Date	10/22/2018 4:18:46 PM
Method	la-2.2-energy.m	Operator	BDAL@DE
Sample Name	EN-04 (Z2KE-II) pos	Instrument	compact 8255754.20088
Comment			

Acquisition Parameter

Source Type	ESI	Ion Polarity	Positive	Set Nebulizer	0.4 Bar
Focus	Active	Set Capillary	4500 V	Set Dry Heater	180 °C
Scan Begin	50 m/z	Set End Plate Offset	-500 V	Set Dry Gas	6.0 l/min
Scan End	3000 m/z	Set Charging Voltage	2000 V	Set Divert Valve	Source
		Set Corona	0 nA	Set APCI Heater	0 °C

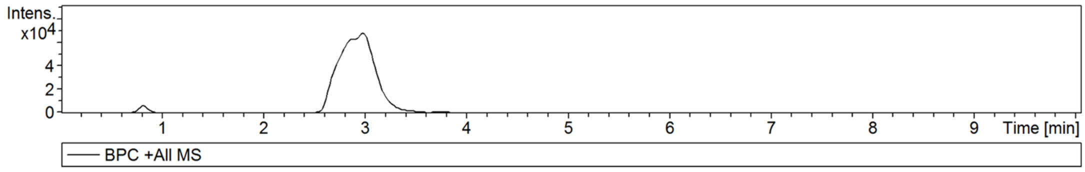

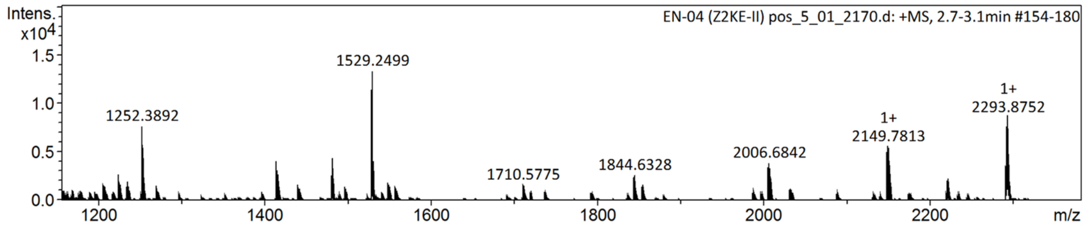

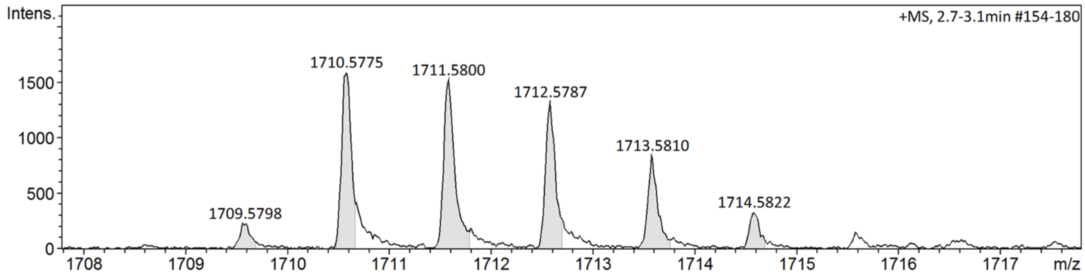

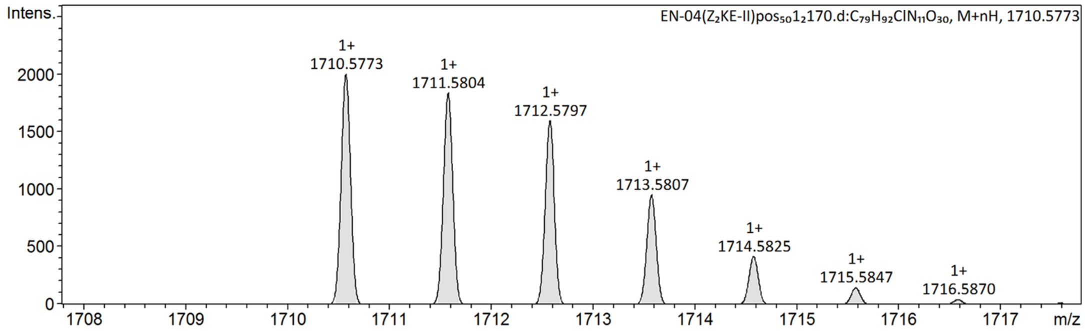

Figure S33. HRMS spectrum of 3,6'-di-Cbz-kanamycinyl A 1-amide of eremomycin (**6**)

Fragmentation ion $[M+H]^+$:

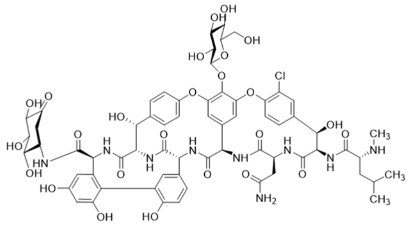

Display Report

Analysis Info

Analysis Name	D:\Data\EN-04 (Z2KE-II) pos_5_01_2170.d	Acquisition Date	10/22/2018 4:18:46 PM
Method	la-2.2-energy.m	Operator	BDAL@DE
Sample Name	EN-04 (Z2KE-II) pos	Instrument	compact 8255754.20088
Comment			

Acquisition Parameter

Source Type	ESI	Ion Polarity	Positive	Set Nebulizer	0.4 Bar
Focus	Active	Set Capillary	4500 V	Set Dry Heater	180 °C
Scan Begin	50 m/z	Set End Plate Offset	-500 V	Set Dry Gas	6.0 l/min
Scan End	3000 m/z	Set Charging Voltage	2000 V	Set Divert Valve	Source
		Set Corona	0 nA	Set APCI Heater	0 °C

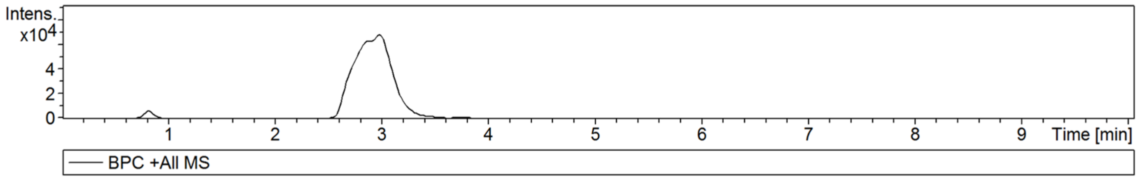

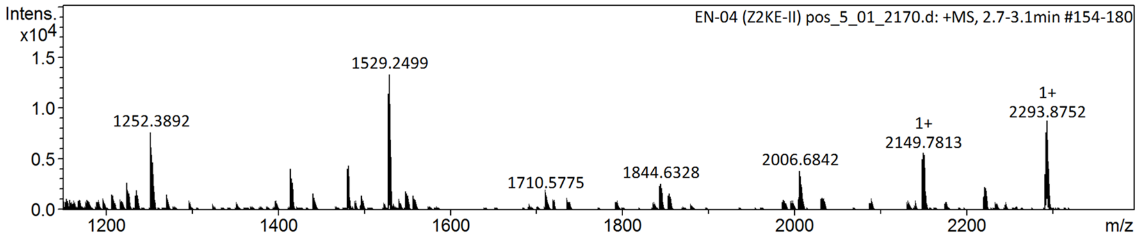

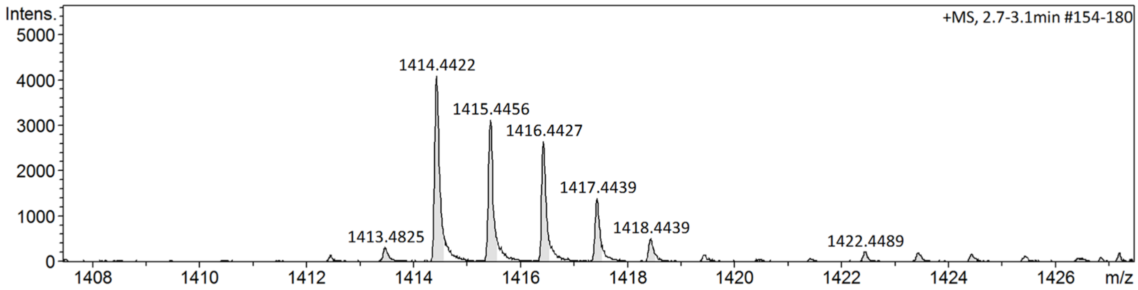

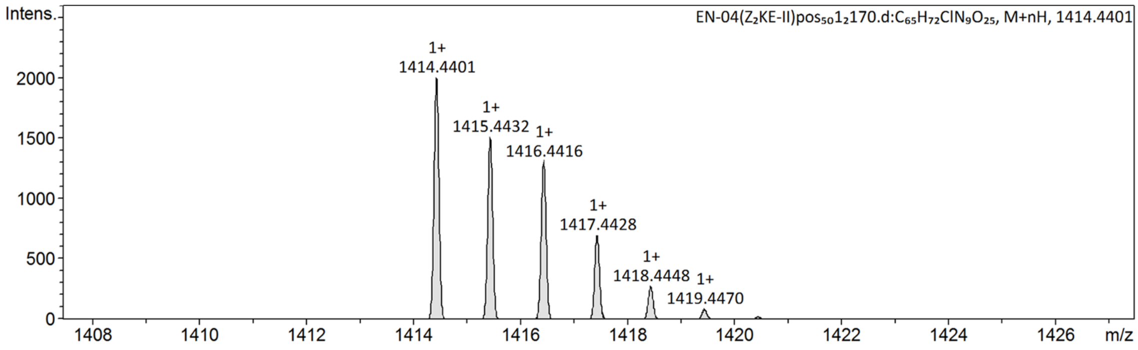

Figure S34. HRMS spectrum of 3,6'-di-Cbz-kanamycinyl A 1-amide of eremomycin (**6**)

Fragmentation ions $[M+H]^+$:

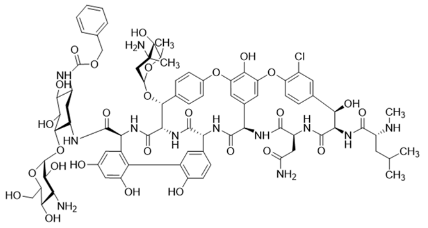

and

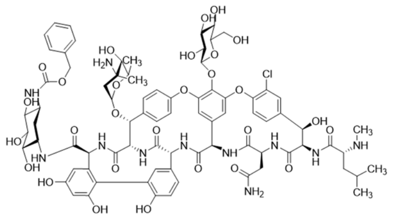

Display Report

Analysis Info

Analysis Name	D:\Data\EN-04 (Z2KE-II) pos_5_01_2170.d	Acquisition Date	10/22/2018 4:18:46 PM
Method	la-2.2-energy.m	Operator	BDAL@DE
Sample Name	EN-04 (Z2KE-II) pos	Instrument	compact 8255754.20088
Comment			

Acquisition Parameter

Source Type	ESI	Ion Polarity	Positive	Set Nebulizer	0.4 Bar
Focus	Active	Set Capillary	4500 V	Set Dry Heater	180 °C
Scan Begin	50 m/z	Set End Plate Offset	-500 V	Set Dry Gas	6.0 l/min
Scan End	3000 m/z	Set Charging Voltage	2000 V	Set Divert Valve	Source
		Set Corona	0 nA	Set APCI Heater	0 °C

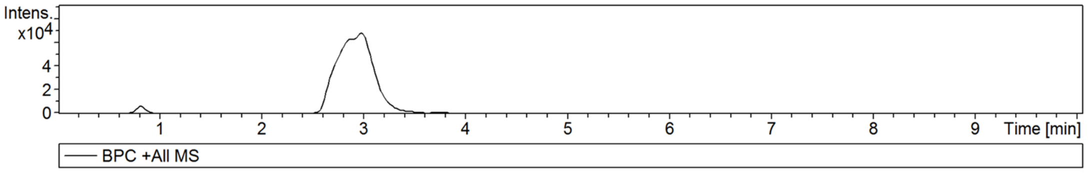

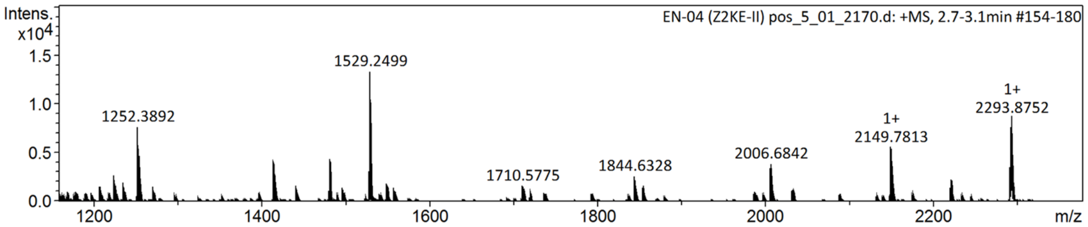

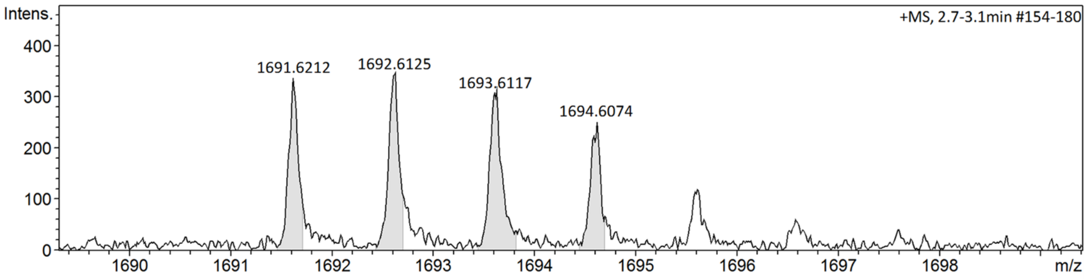

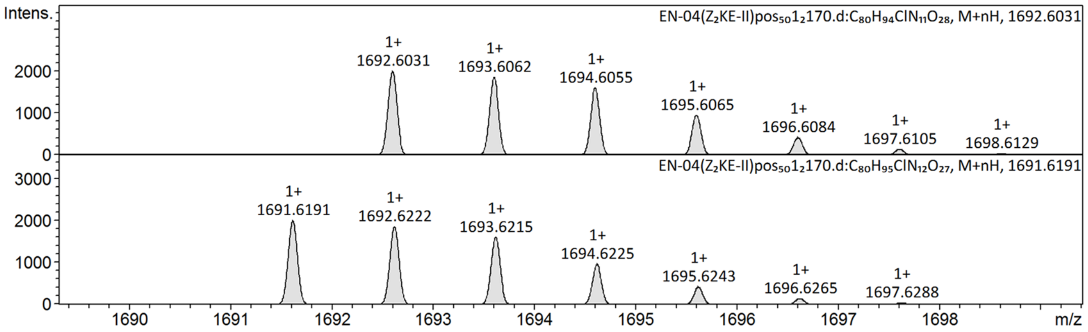

Figure S35. HRMS spectrum of 3,6'-di-Cbz-kanamycinyl A 1-amide of eremomycin (**6**)

Fragmentation ion [M+H]⁺:

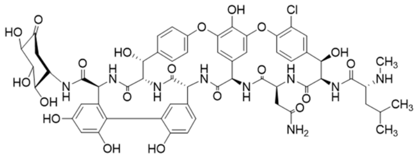

Display Report

Analysis Info

Analysis Name	D:\Data\EN-04 (Z2KE-II) pos_5_01_2170.d	Acquisition Date	10/22/2018 4:18:46 PM
Method	la-2.2-energy.m	Operator	BDAL@DE
Sample Name	EN-04 (Z2KE-II) pos	Instrument	compact 8255754.20088
Comment			

Acquisition Parameter

Source Type	ESI	Ion Polarity	Positive	Set Nebulizer	0.4 Bar
Focus	Active	Set Capillary	4500 V	Set Dry Heater	180 °C
Scan Begin	50 m/z	Set End Plate Offset	-500 V	Set Dry Gas	6.0 l/min
Scan End	3000 m/z	Set Charging Voltage	2000 V	Set Divert Valve	Source
		Set Corona	0 nA	Set APCI Heater	0 °C

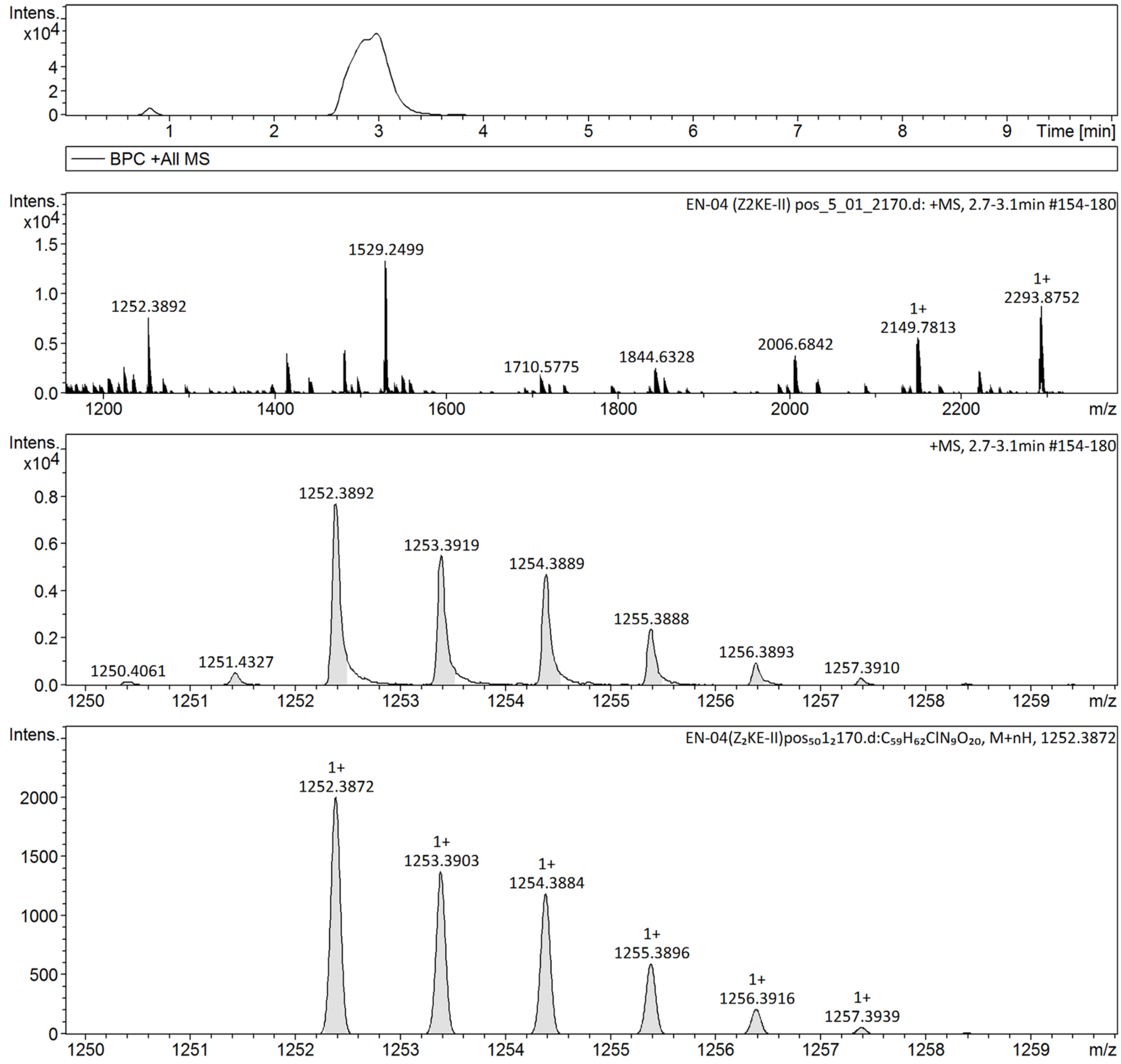

Figure S36. HRMS spectrum of 3,6'-di-Cbz-kanamycinyl A 1-amide of eremomycin (**6**)

Molecular ion $[M+H]^+$ (m/z, z=2):

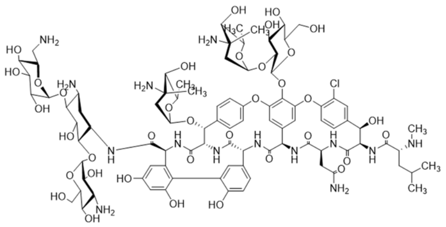

Display Report

Analysis Info

Analysis Name	D:\Data\EN-04 (Z2KE-II) pos_5_01_2170.d	Acquisition Date	10/22/2018 4:18:46 PM
Method	la-2.2-energy.m	Operator	BDAL@DE
Sample Name	EN-04 (Z2KE-II) pos	Instrument	compact 8255754.20088
Comment			

Acquisition Parameter

Source Type	ESI	Ion Polarity	Positive	Set Nebulizer	0.4 Bar
Focus	Active	Set Capillary	4500 V	Set Dry Heater	180 °C
Scan Begin	50 m/z	Set End Plate Offset	-500 V	Set Dry Gas	6.0 l/min
Scan End	3000 m/z	Set Charging Voltage	2000 V	Set Divert Valve	Source
		Set Corona	0 nA	Set APCI Heater	0 °C

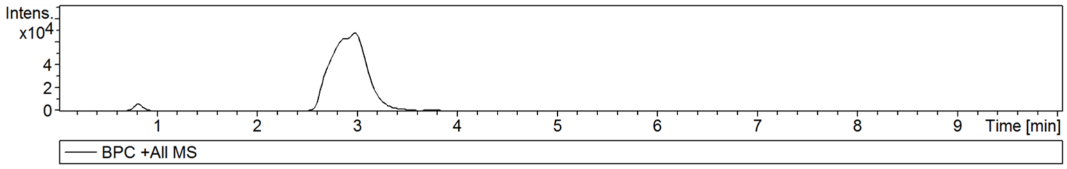

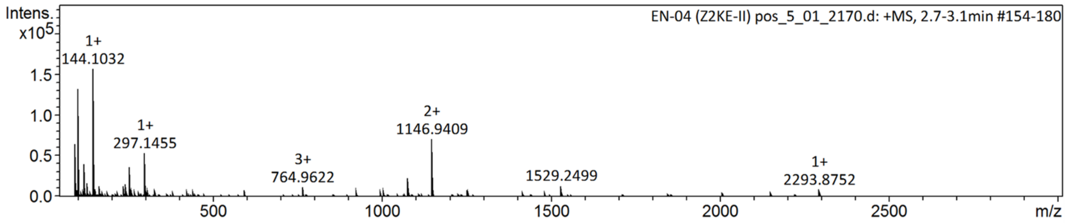

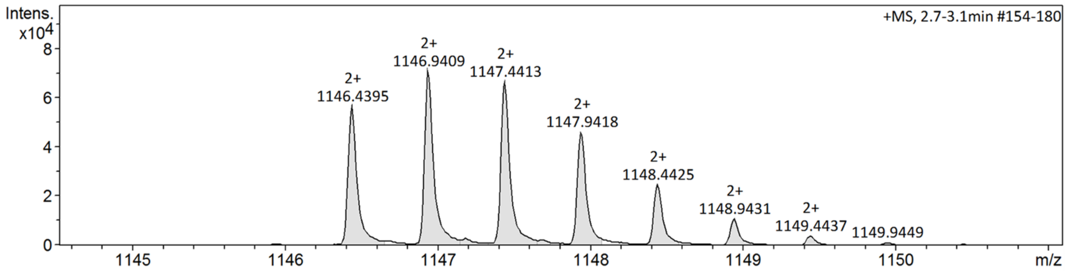

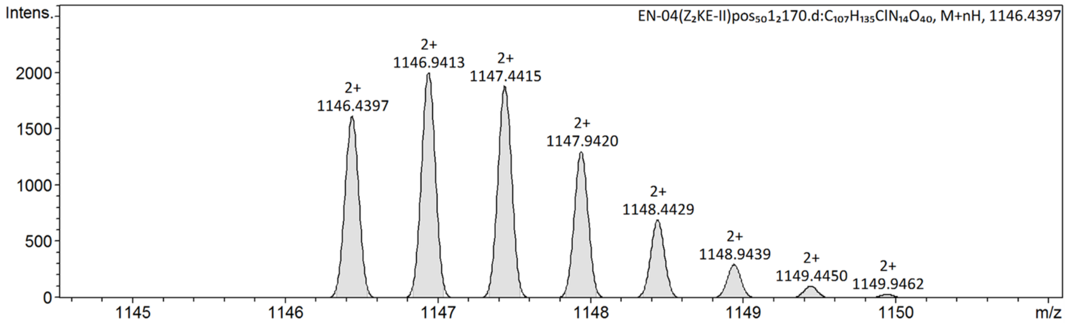

Kanamycinyl A 1-amide of eremomycin (7)

Figure S37. UV spectra of kanamycinyl A 1-amide of eremomycin (7)

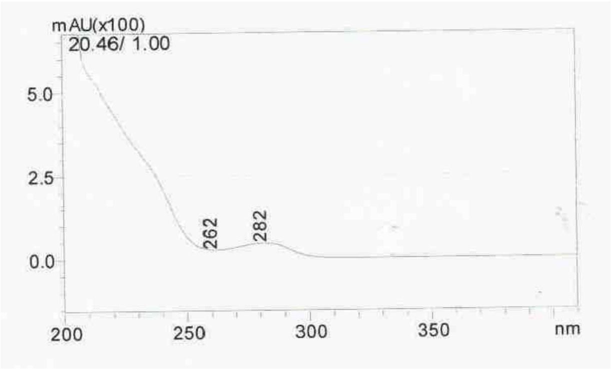

Figure S38. IR spectra of kanamycinyl A 1-amide of eremomycin (7)

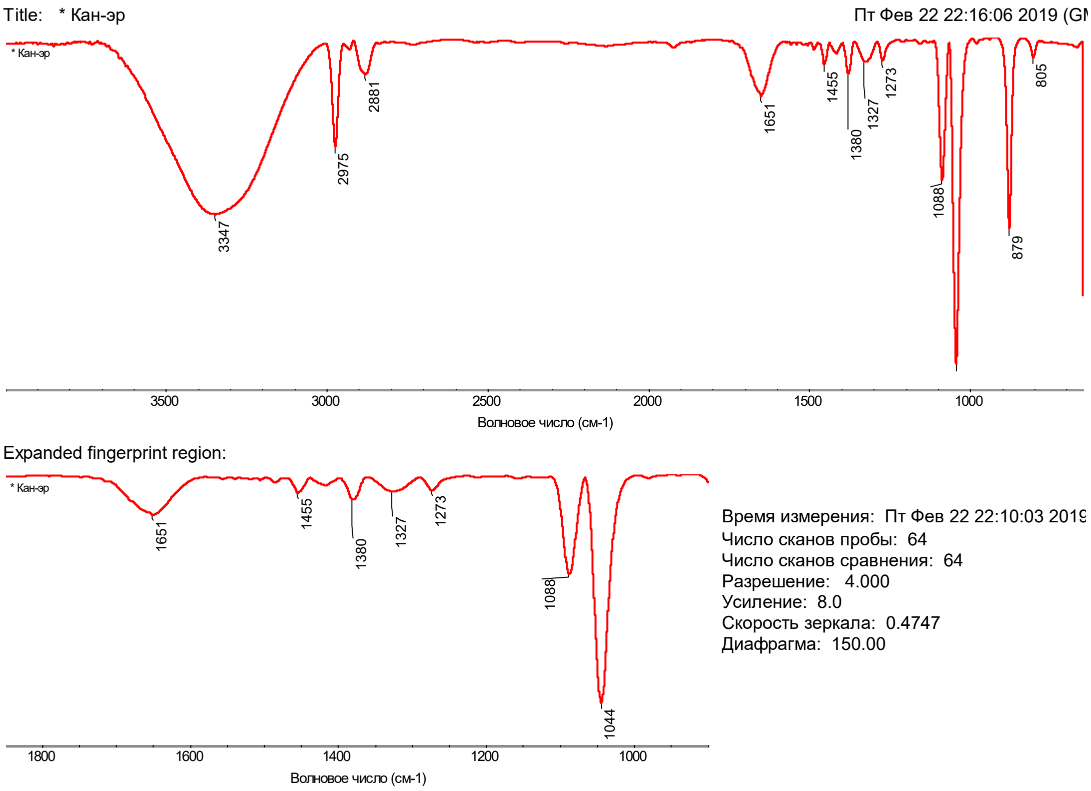

Figure S39. ¹H NMR spectrum of kanamycinyl A 1-amide of eremomycin (**7**)

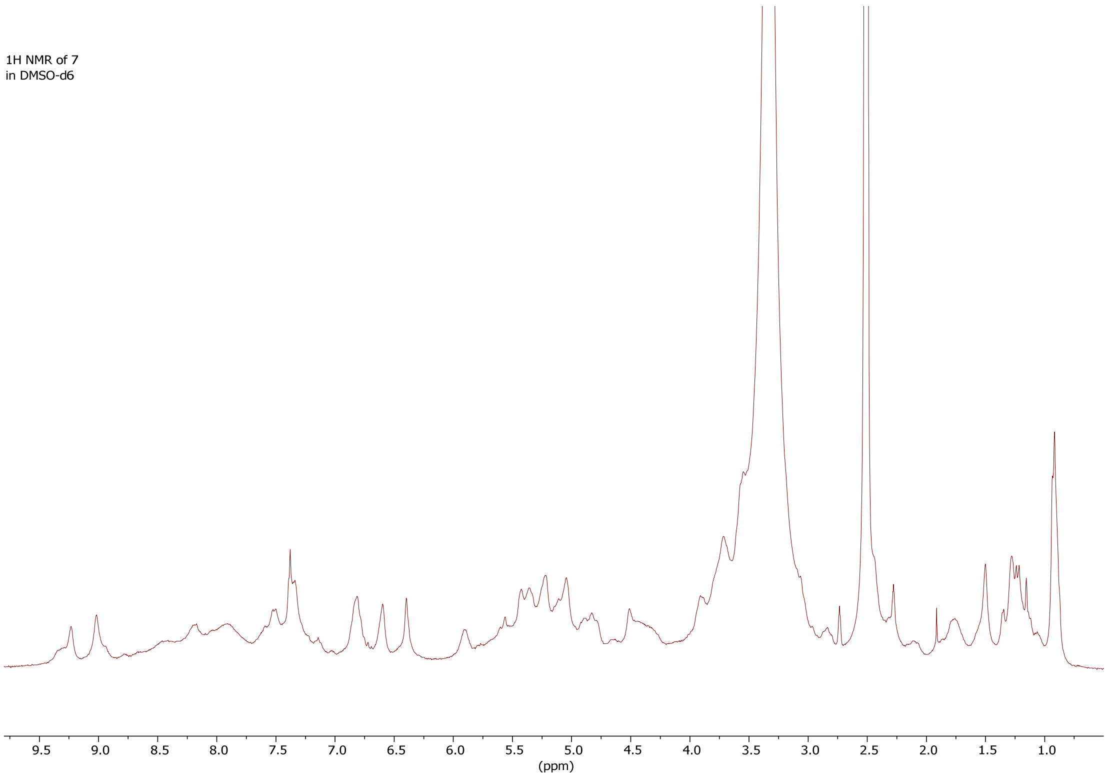

Figure S40. HSQC NMR spectrum of kanamycinyl A 1-amide of eremomycin (**7**)

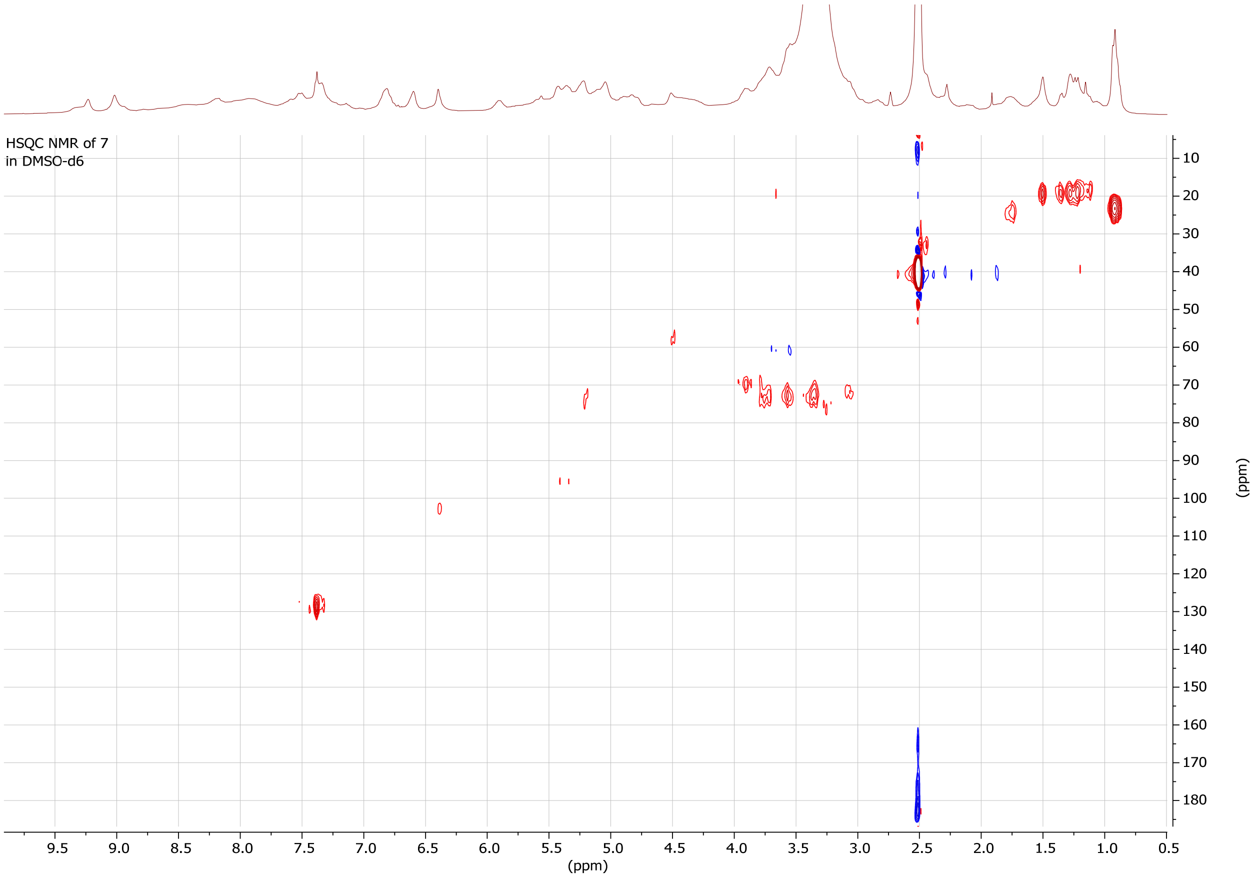

Figure S41. HRMS spectrum of kanamycinyl A 1-amide of eremomycin (**7**)

Molecular ion $[M+H]^+$ (m/z, z=1):

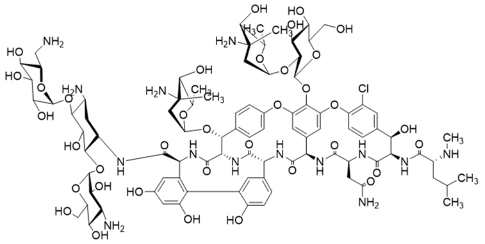

Display Report

Analysis Info

Analysis Name	D:\Data\EN-05 (KE) pos_6_01_2171.d	Acquisition Date	10/22/2018 4:30:30 PM
Method	la-2.2-energy.m	Operator	BDAL@DE
Sample Name	EN-05 (KE) pos	Instrument	compact 8255754.20088
Comment			

Acquisition Parameter

Source Type	ESI	Ion Polarity	Positive	Set Nebulizer	0.4 Bar
Focus	Active	Set Capillary	4500 V	Set Dry Heater	180 °C
Scan Begin	50 m/z	Set End Plate Offset	-500 V	Set Dry Gas	6.0 l/min
Scan End	3000 m/z	Set Charging Voltage	2000 V	Set Divert Valve	Source
		Set Corona	0 nA	Set APCI Heater	0 °C

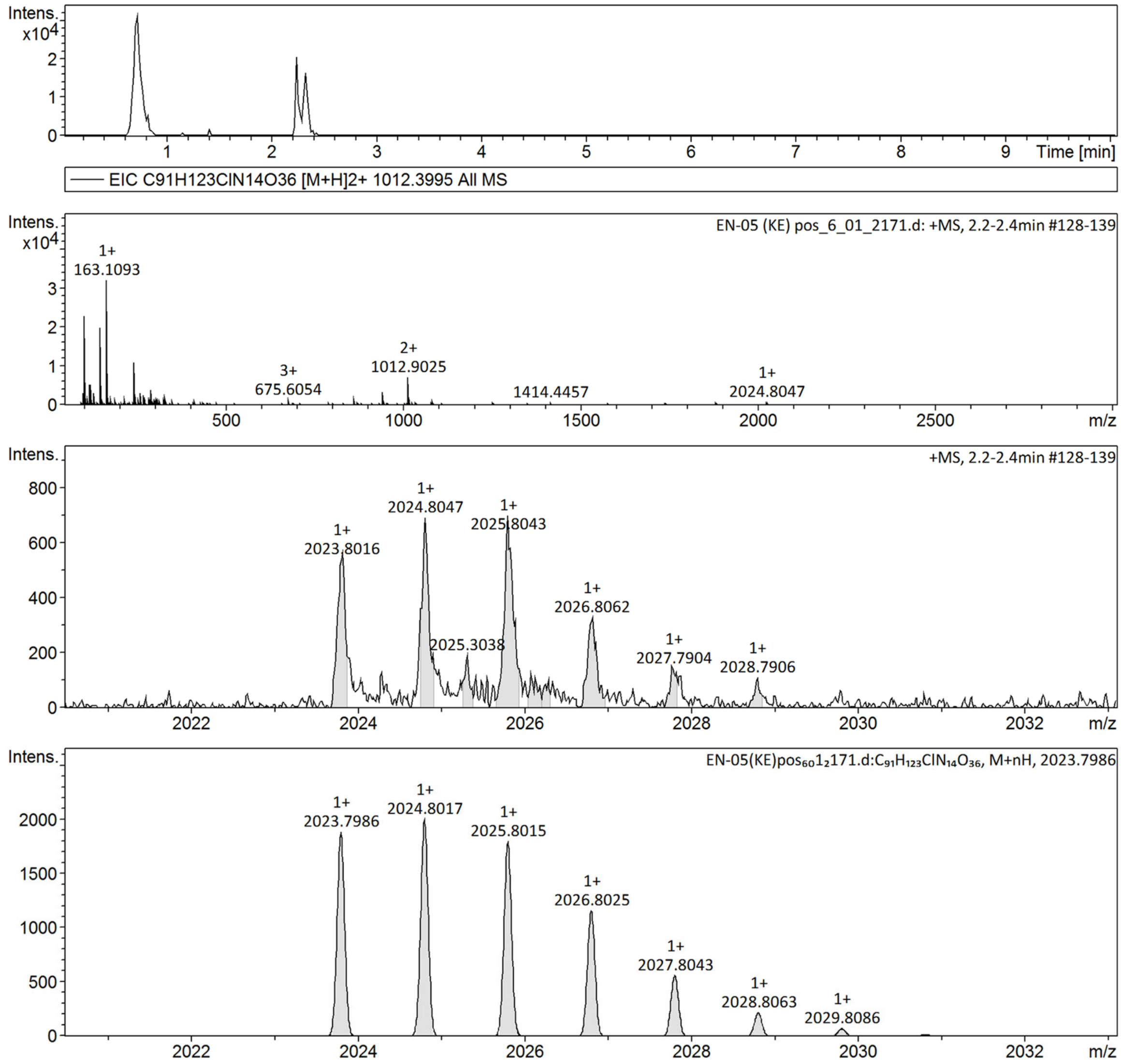

Figure S42. HRMS spectrum of kanamycinyl A 1-amide of eremomycin (**7**)

Fragmentation ion [M+H]$^+$:

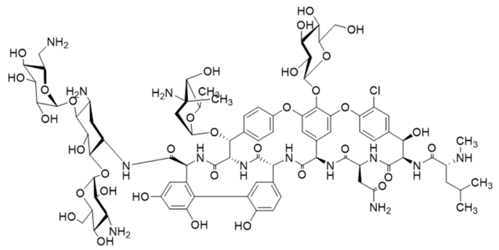

Display Report

Analysis Info

Analysis Name	D:\Data\EN-05 (KE) pos_6_01_2171.d	Acquisition Date	10/22/2018 4:30:30 PM
Method	la-2.2-energy.m	Operator	BDAL@DE
Sample Name	EN-05 (KE) pos	Instrument	compact 8255754.20088
Comment			

Acquisition Parameter

Source Type	ESI	Ion Polarity	Positive	Set Nebulizer	0.4 Bar
Focus	Active	Set Capillary	4500 V	Set Dry Heater	180 °C
Scan Begin	50 m/z	Set End Plate Offset	-500 V	Set Dry Gas	6.0 l/min
Scan End	3000 m/z	Set Charging Voltage	2000 V	Set Divert Valve	Source
		Set Corona	0 nA	Set APCI Heater	0 °C

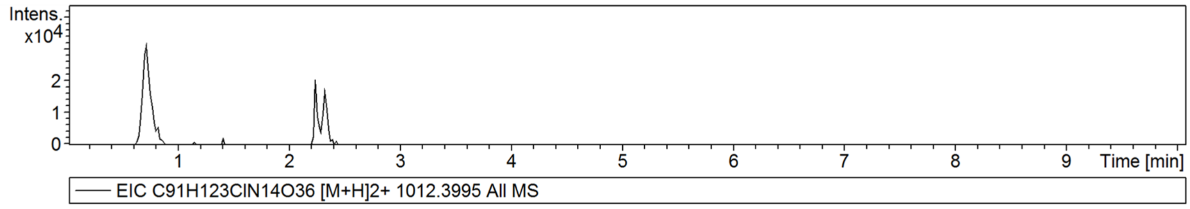

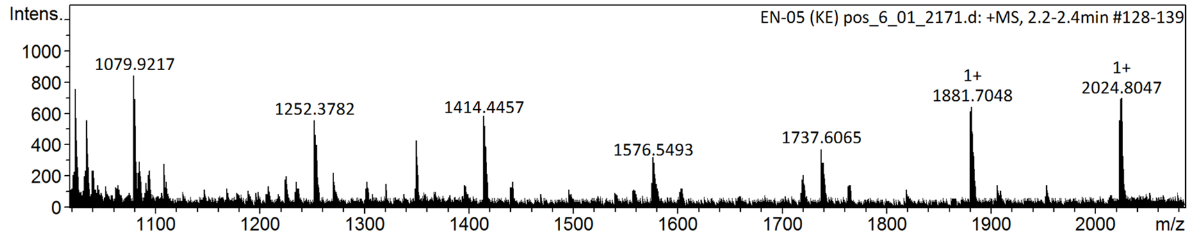

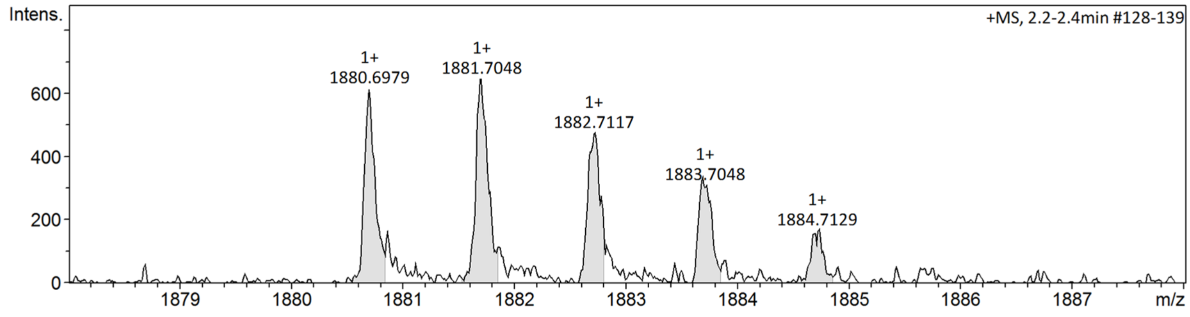

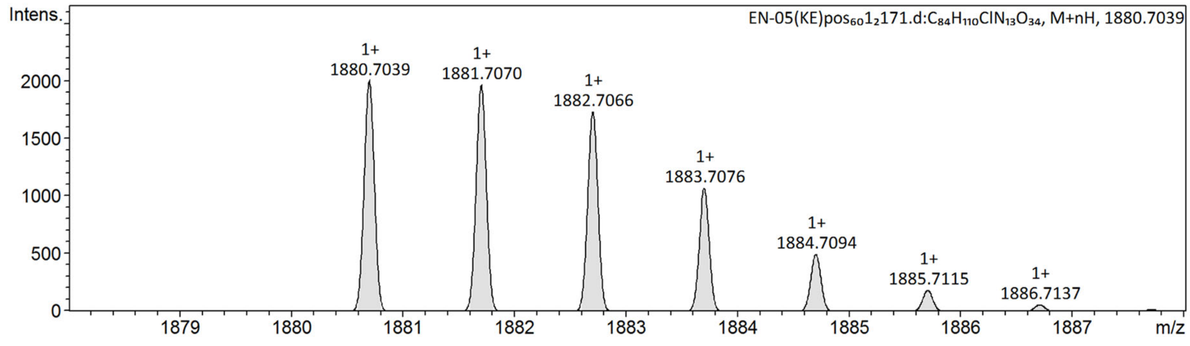

Figure S43. HRMS spectrum of kanamycinyl A 1-amide of eremomycin (**7**)

Fragmentation ion $[M+H]^+$:

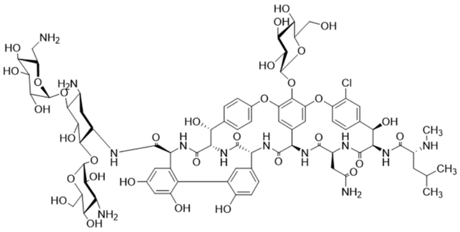

Display Report

Analysis Info

Analysis Name	D:\Data\EN-05 (KE) pos_6_01_2171.d	Acquisition Date	10/22/2018 4:30:30 PM
Method	la-2.2-energy.m	Operator	BDAL@DE
Sample Name	EN-05 (KE) pos	Instrument	compact 8255754.20088
Comment			

Acquisition Parameter

Source Type	ESI	Ion Polarity	Positive	Set Nebulizer	0.4 Bar
Focus	Active	Set Capillary	4500 V	Set Dry Heater	180 °C
Scan Begin	50 m/z	Set End Plate Offset	-500 V	Set Dry Gas	6.0 l/min
Scan End	3000 m/z	Set Charging Voltage	2000 V	Set Divert Valve	Source
		Set Corona	0 nA	Set APCI Heater	0 °C

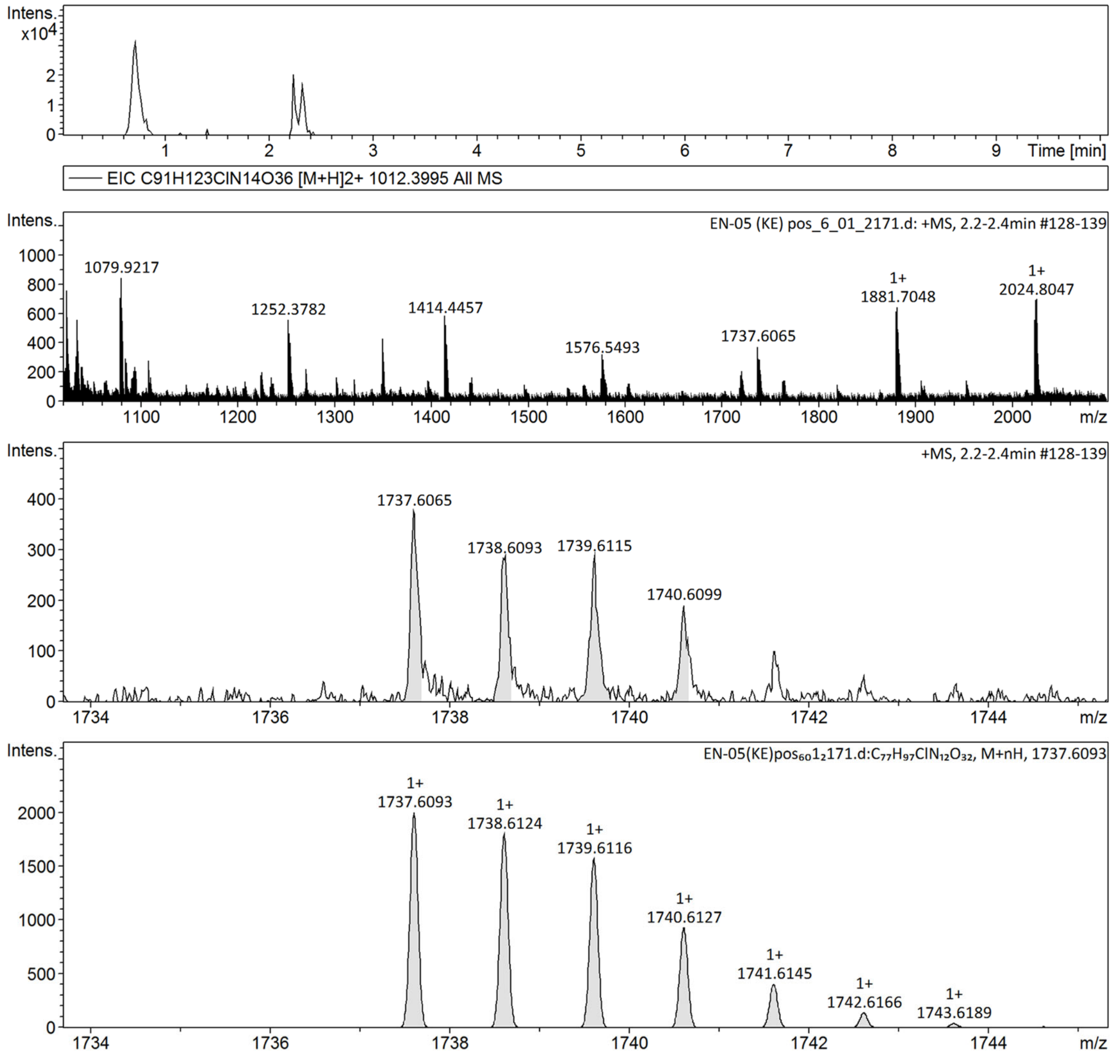

Figure S44. HRMS spectrum of kanamycinyl A 1-amide of eremomycin (**7**)

Fragmentation ion $[M+H]^+$:

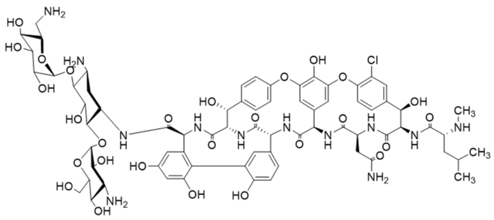

Display Report

Analysis Info

Analysis Name	D:\Data\EN-05 (KE) pos_6_01_2171.d	Acquisition Date	10/22/2018 4:30:30 PM
Method	la-2.2-energy.m	Operator	BDAL@DE
Sample Name	EN-05 (KE) pos	Instrument	compact 8255754.20088
Comment			

Acquisition Parameter

Source Type	ESI	Ion Polarity	Positive	Set Nebulizer	0.4 Bar
Focus	Active	Set Capillary	4500 V	Set Dry Heater	180 °C
Scan Begin	50 m/z	Set End Plate Offset	-500 V	Set Dry Gas	6.0 l/min
Scan End	3000 m/z	Set Charging Voltage	2000 V	Set Divert Valve	Source
		Set Corona	0 nA	Set APCI Heater	0 °C

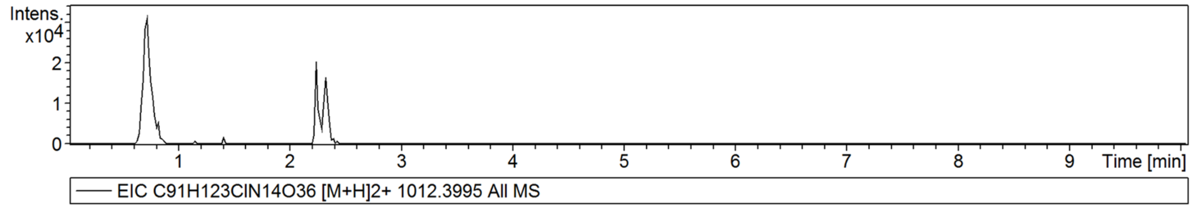

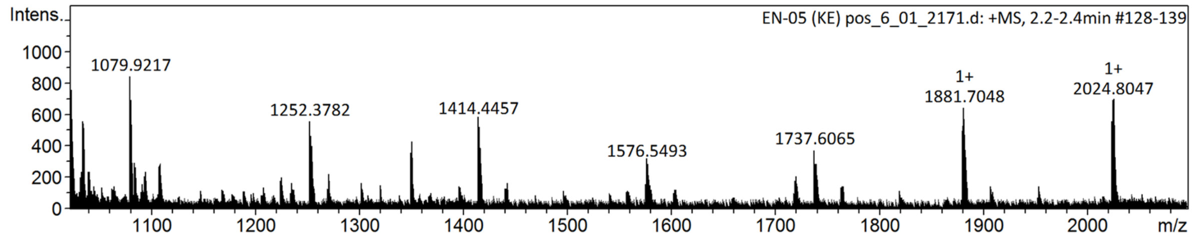

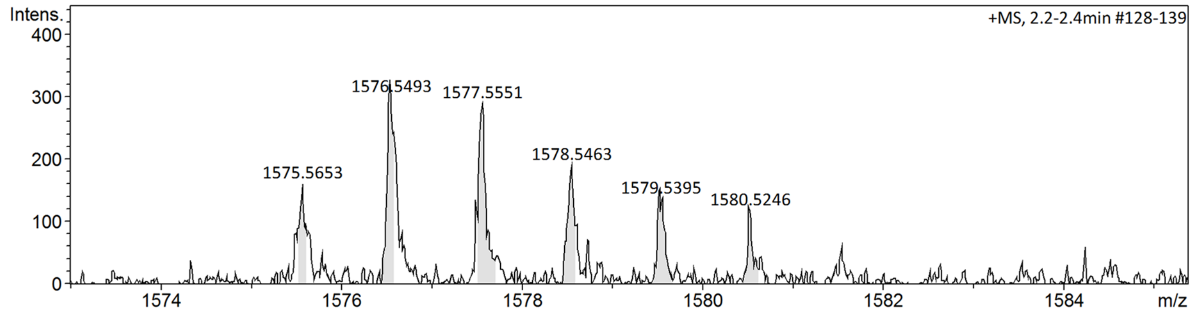

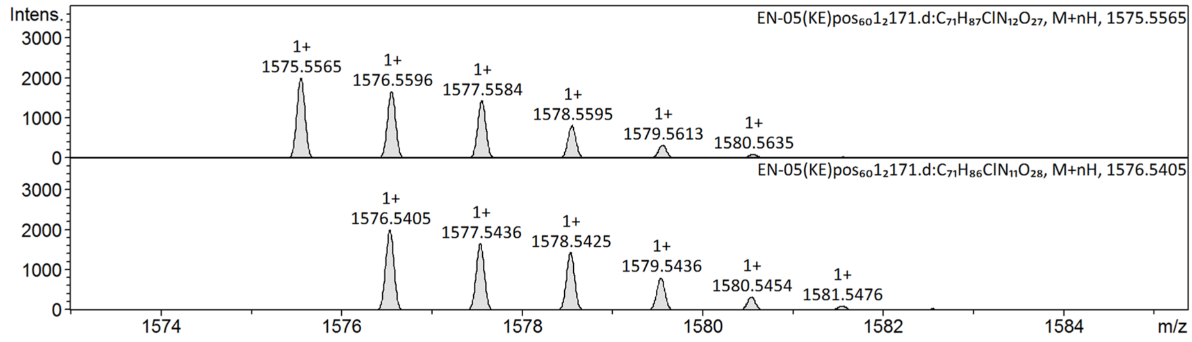

Figure S45. HRMS spectrum of kanamycinyl A 1-amide of eremomycin (**7**)

Fragmentation ion $[M+H]^+$:

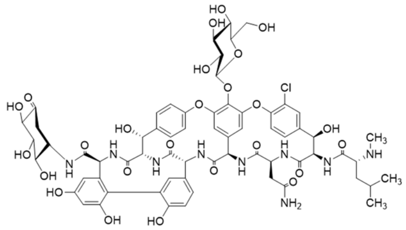

Display Report

Analysis Info

Analysis Name	D:\Data\EN-05 (KE) pos_6_01_2171.d	Acquisition Date	10/22/2018 4:30:30 PM
Method	la-2.2-energy.m	Operator	BDAL@DE
Sample Name	EN-05 (KE) pos	Instrument	compact 8255754.20088
Comment			

Acquisition Parameter

Source Type	ESI	Ion Polarity	Positive	Set Nebulizer	0.4 Bar
Focus	Active	Set Capillary	4500 V	Set Dry Heater	180 °C
Scan Begin	50 m/z	Set End Plate Offset	-500 V	Set Dry Gas	6.0 l/min
Scan End	3000 m/z	Set Charging Voltage	2000 V	Set Divert Valve	Source
		Set Corona	0 nA	Set APCI Heater	0 °C

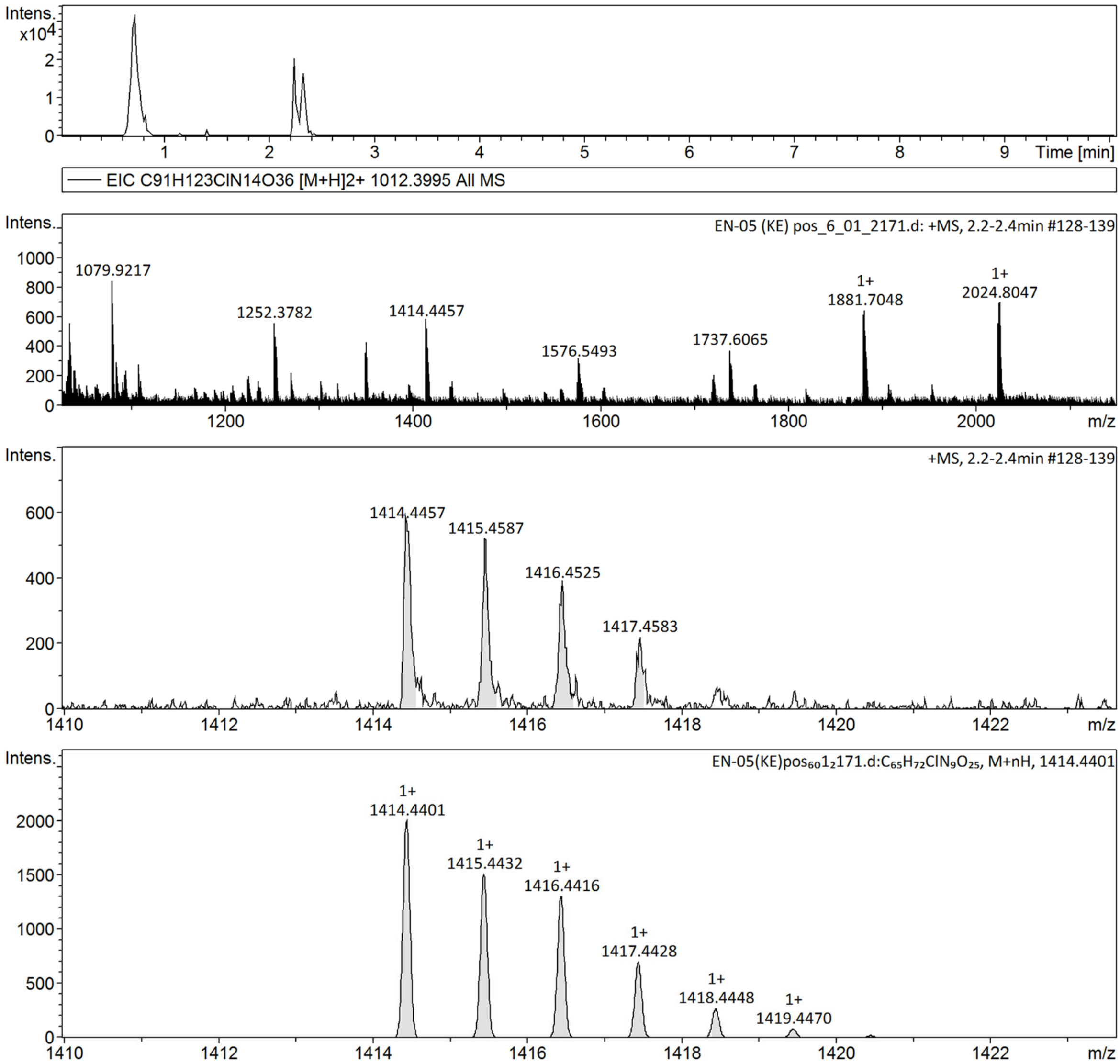

Figure S46. HRMS spectrum of kanamycinyl A 1-amide of eremomycin (7)

Fragmentation ion $[M+H]^+$:

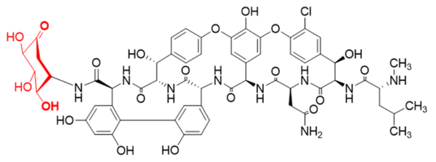

Display Report

Analysis Info

Analysis Name	D:\Data\EN-05 (KE) pos_6_01_2171.d	Acquisition Date	10/22/2018 4:30:30 PM
Method	la-2.2-energy.m	Operator	BDAL@DE
Sample Name	EN-05 (KE) pos	Instrument	compact 8255754.20088
Comment			

Acquisition Parameter

Source Type	ESI	Ion Polarity	Positive	Set Nebulizer	0.4 Bar
Focus	Active	Set Capillary	4500 V	Set Dry Heater	180 °C
Scan Begin	50 m/z	Set End Plate Offset	-500 V	Set Dry Gas	6.0 l/min
Scan End	3000 m/z	Set Charging Voltage	2000 V	Set Divert Valve	Source
		Set Corona	0 nA	Set APCI Heater	0 °C

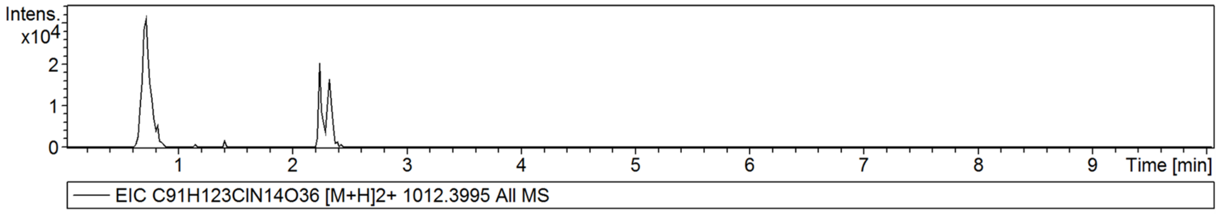

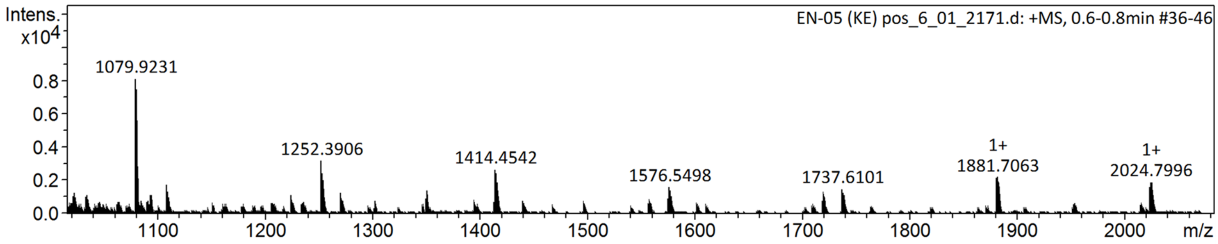

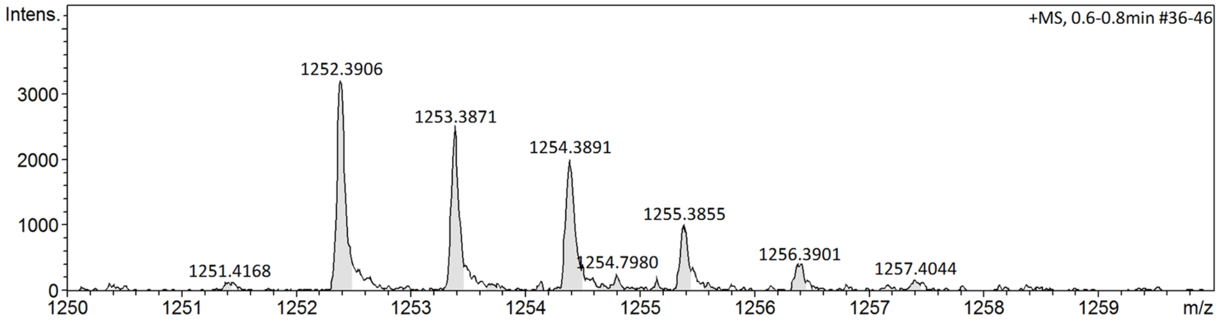

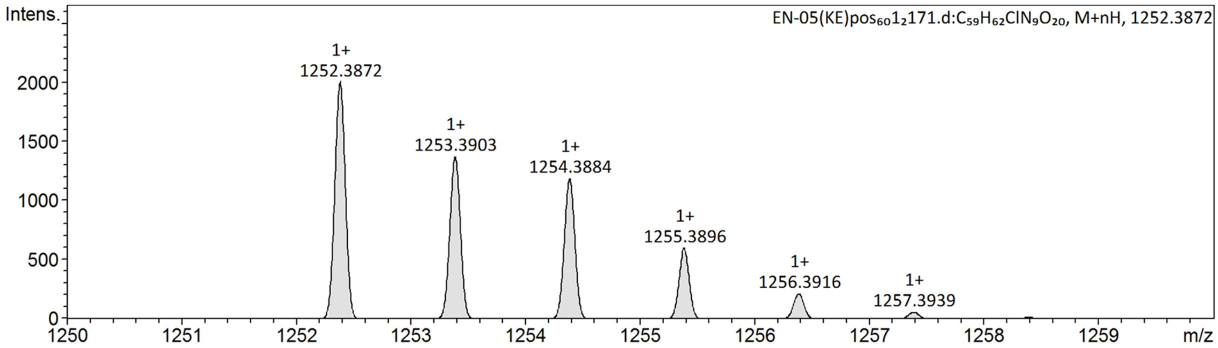

Figure S47. HRMS spectrum of kanamycinyl A 1-amide of eremomycin (**7**)

Fragmentation ion $[M+H]^+$:

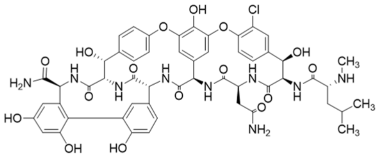

Display Report

Analysis Info

Analysis Name	D:\Data\EN-05 (KE) pos_6_01_2171.d	Acquisition Date	10/22/2018 4:30:30 PM
Method	la-2.2-energy.m	Operator	BDAL@DE
Sample Name	EN-05 (KE) pos	Instrument	compact 8255754.20088
Comment			

Acquisition Parameter

Source Type	ESI	Ion Polarity	Positive	Set Nebulizer	0.4 Bar
Focus	Active	Set Capillary	4500 V	Set Dry Heater	180 °C
Scan Begin	50 m/z	Set End Plate Offset	-500 V	Set Dry Gas	6.0 l/min
Scan End	3000 m/z	Set Charging Voltage	2000 V	Set Divert Valve	Source
		Set Corona	0 nA	Set APCI Heater	0 °C

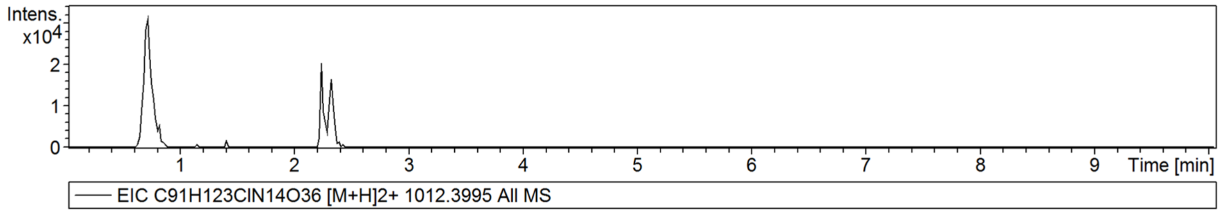

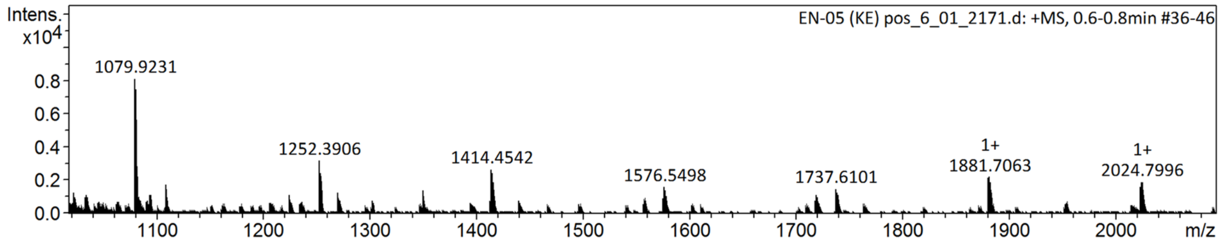

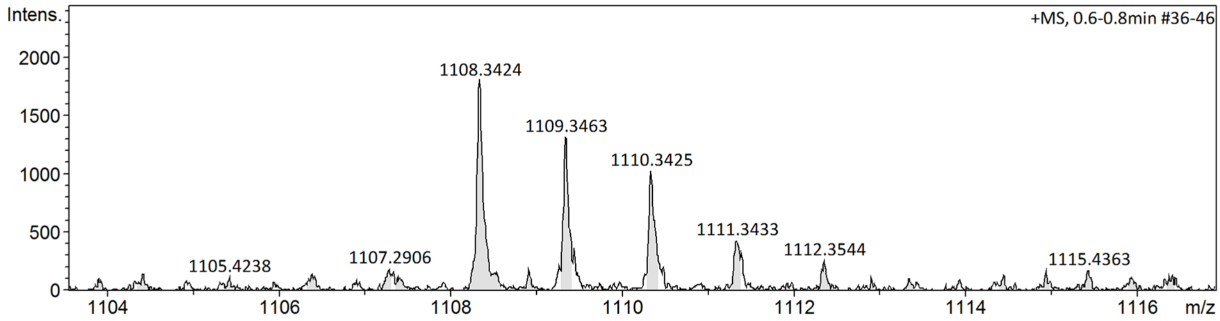

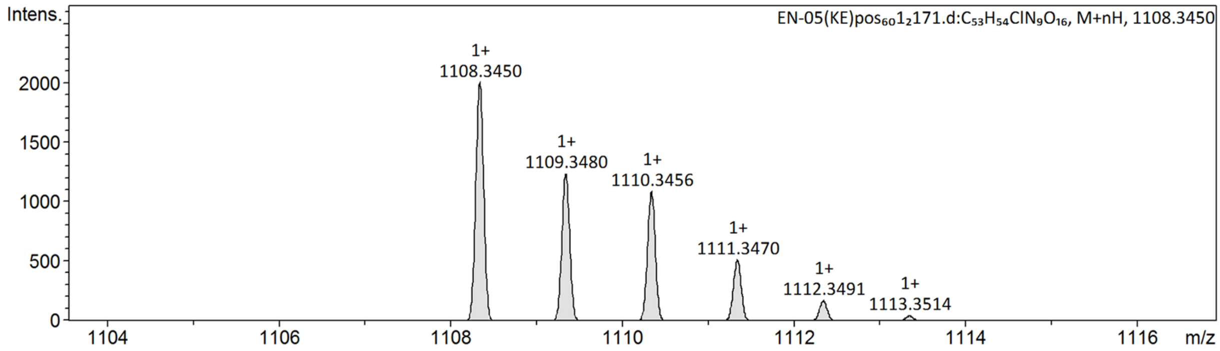

Figure S48. HRMS spectrum of kanamycinyl A 1-amide of eremomycin (**7**)

Molecular ion $[M+H]^+$ (m/z, z=2):

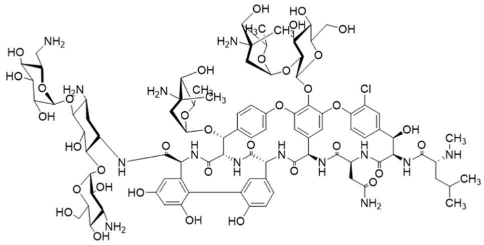

Display Report

Analysis Info

Analysis Name	D:\Data\EN-05 (KE) pos_6_01_2171.d	Acquisition Date	10/22/2018 4:30:30 PM
Method	la-2.2-energy.m	Operator	BDAL@DE
Sample Name	EN-05 (KE) pos	Instrument	compact 8255754.20088
Comment			

Acquisition Parameter

Source Type	ESI	Ion Polarity	Positive	Set Nebulizer	0.4 Bar
Focus	Active	Set Capillary	4500 V	Set Dry Heater	180 °C
Scan Begin	50 m/z	Set End Plate Offset	-500 V	Set Dry Gas	6.0 l/min
Scan End	3000 m/z	Set Charging Voltage	2000 V	Set Divert Valve	Source
		Set Corona	0 nA	Set APCI Heater	0 °C

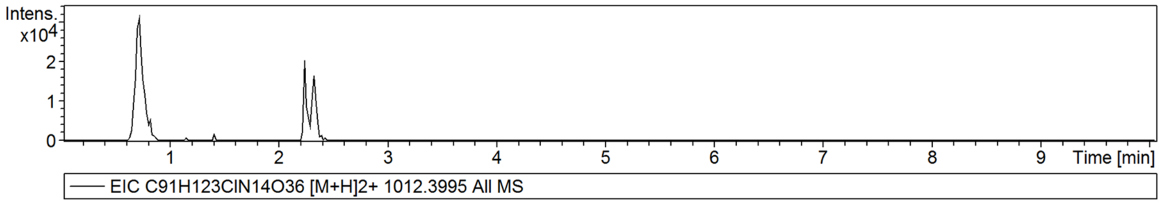

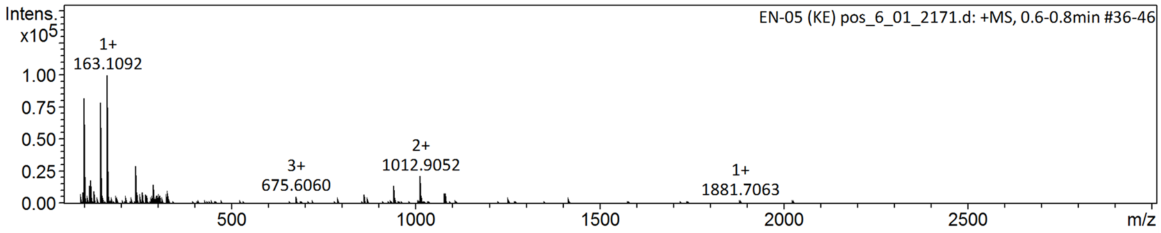

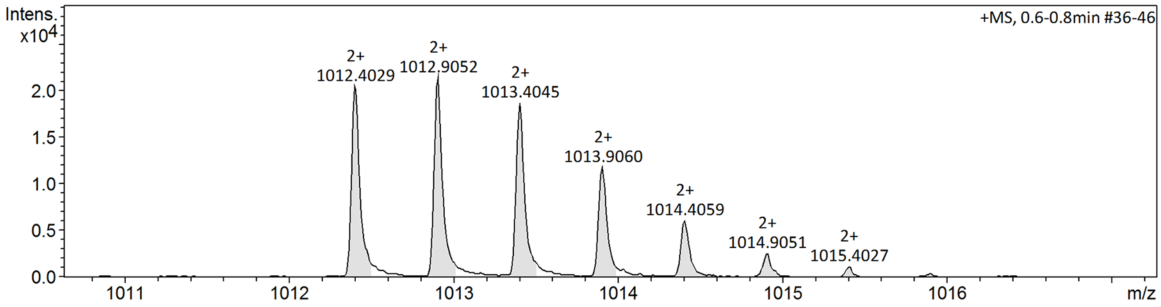

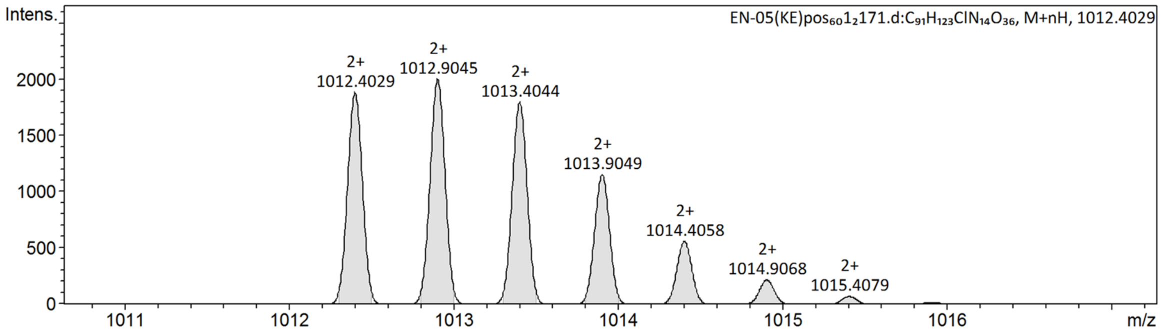

Figure S49. HRMS spectrum of kanamycinyl A 1-amide of eremomycin (**7**)

Molecular ion [M+H]$^+$ (*m*/*z*, *z*=3):

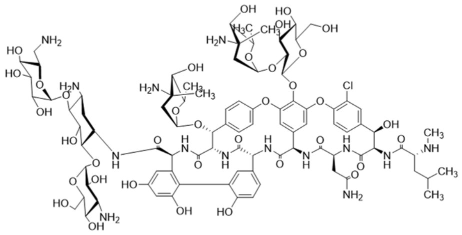

Display Report

Analysis Info

Analysis Name	D:\Data\EN-05 (KE) pos_6_01_2171.d	Acquisition Date	10/22/2018 4:30:30 PM
Method	la-2.2-energy.m	Operator	BDAL@DE
Sample Name	EN-05 (KE) pos	Instrument	compact 8255754.20088
Comment			

Acquisition Parameter

Source Type	ESI	Ion Polarity	Positive	Set Nebulizer	0.4 Bar
Focus	Active	Set Capillary	4500 V	Set Dry Heater	180 °C
Scan Begin	50 m/z	Set End Plate Offset	-500 V	Set Dry Gas	6.0 l/min
Scan End	3000 m/z	Set Charging Voltage	2000 V	Set Divert Valve	Source
		Set Corona	0 nA	Set APCI Heater	0 °C

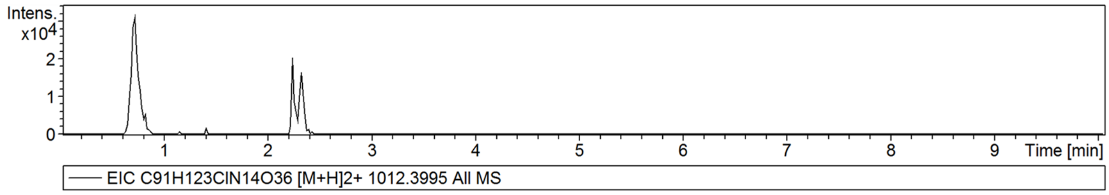

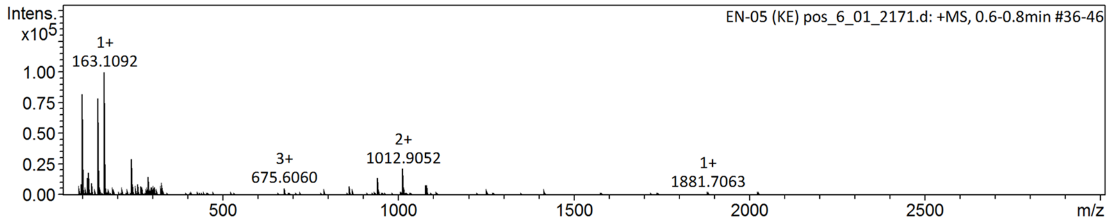

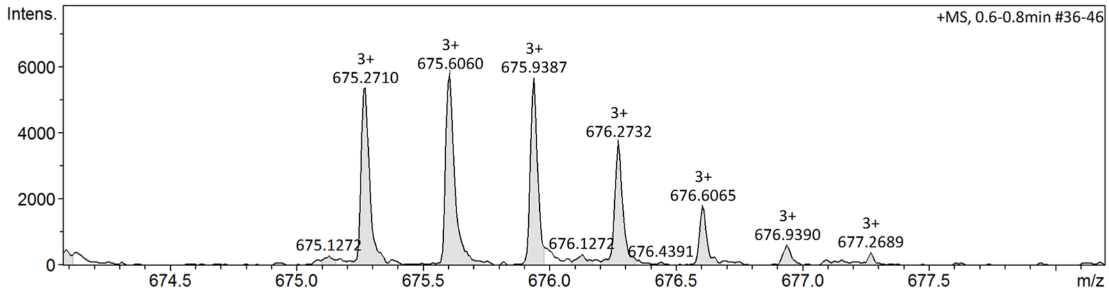

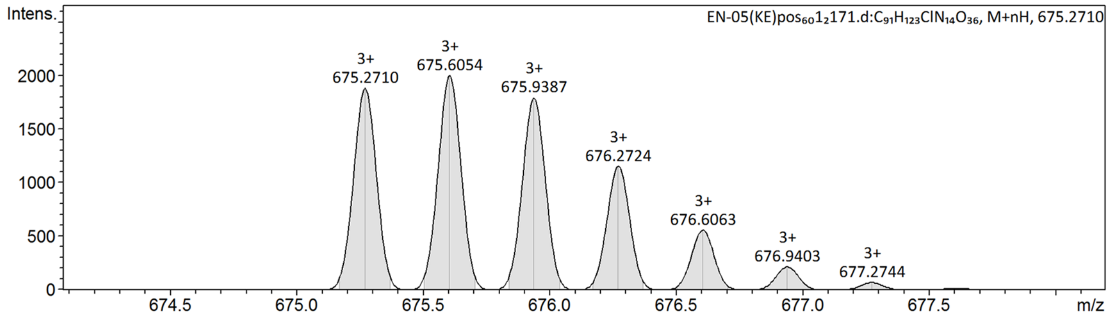

Figure S50. HRMS spectrum of kanamycinyl A 1-amide of eremomycin (**7**)

Fragmentation ions [M+H]$^+$:

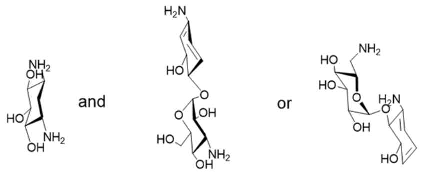

Display Report

Analysis Info

Analysis Name	D:\Data\EN-05 (KE) pos_6_01_2171.d	Acquisition Date	10/22/2018 4:30:30 PM
Method	la-2.2-energy.m	Operator	BDAL@DE
Sample Name	EN-05 (KE) pos	Instrument	compact 8255754.20088
Comment			

Acquisition Parameter

Source Type	ESI	Ion Polarity	Positive	Set Nebulizer	0.4 Bar
Focus	Active	Set Capillary	4500 V	Set Dry Heater	180 °C
Scan Begin	50 m/z	Set End Plate Offset	-500 V	Set Dry Gas	6.0 l/min
Scan End	3000 m/z	Set Charging Voltage	2000 V	Set Divert Valve	Source
		Set Corona	0 nA	Set APCI Heater	0 °C

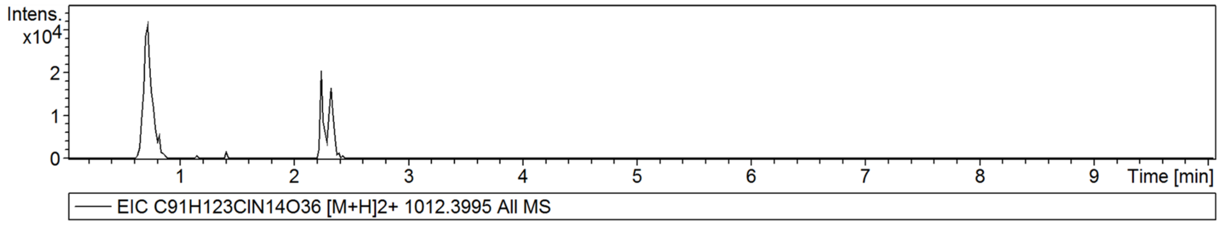

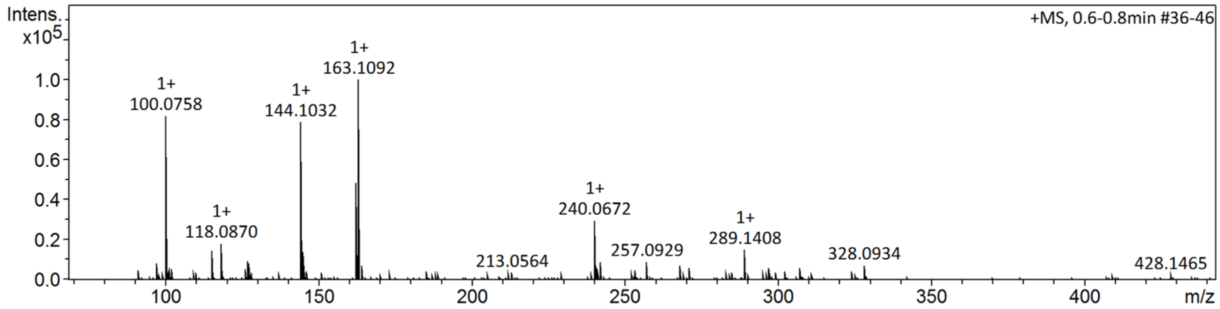

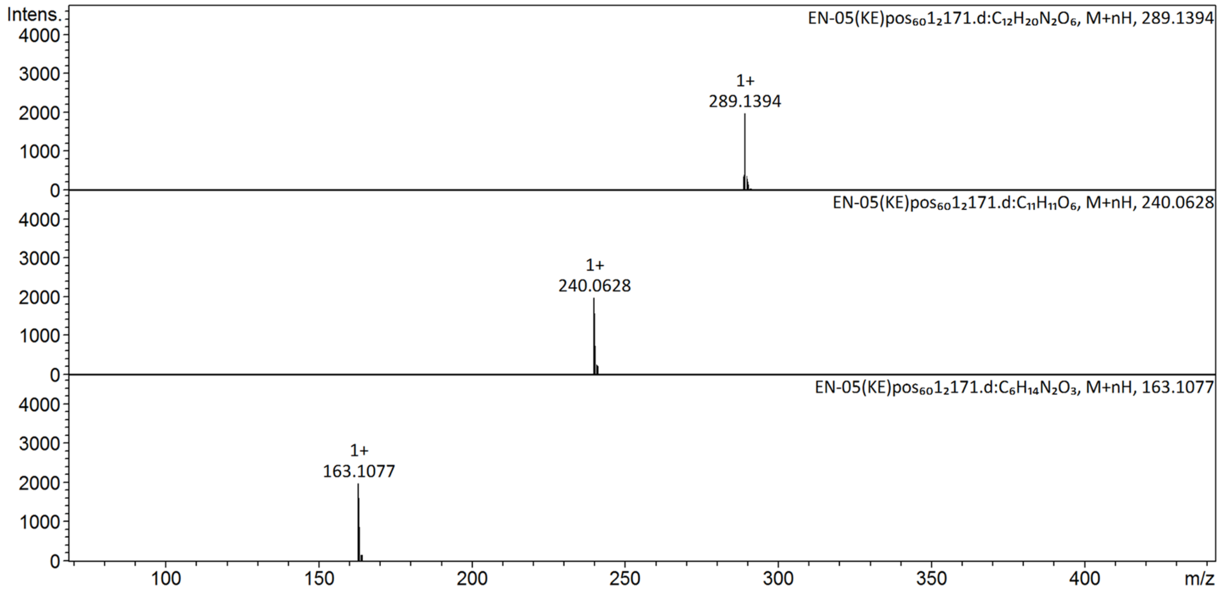

Figure S51. ^{1}H NMR spectrum of vancomycin (**1**)

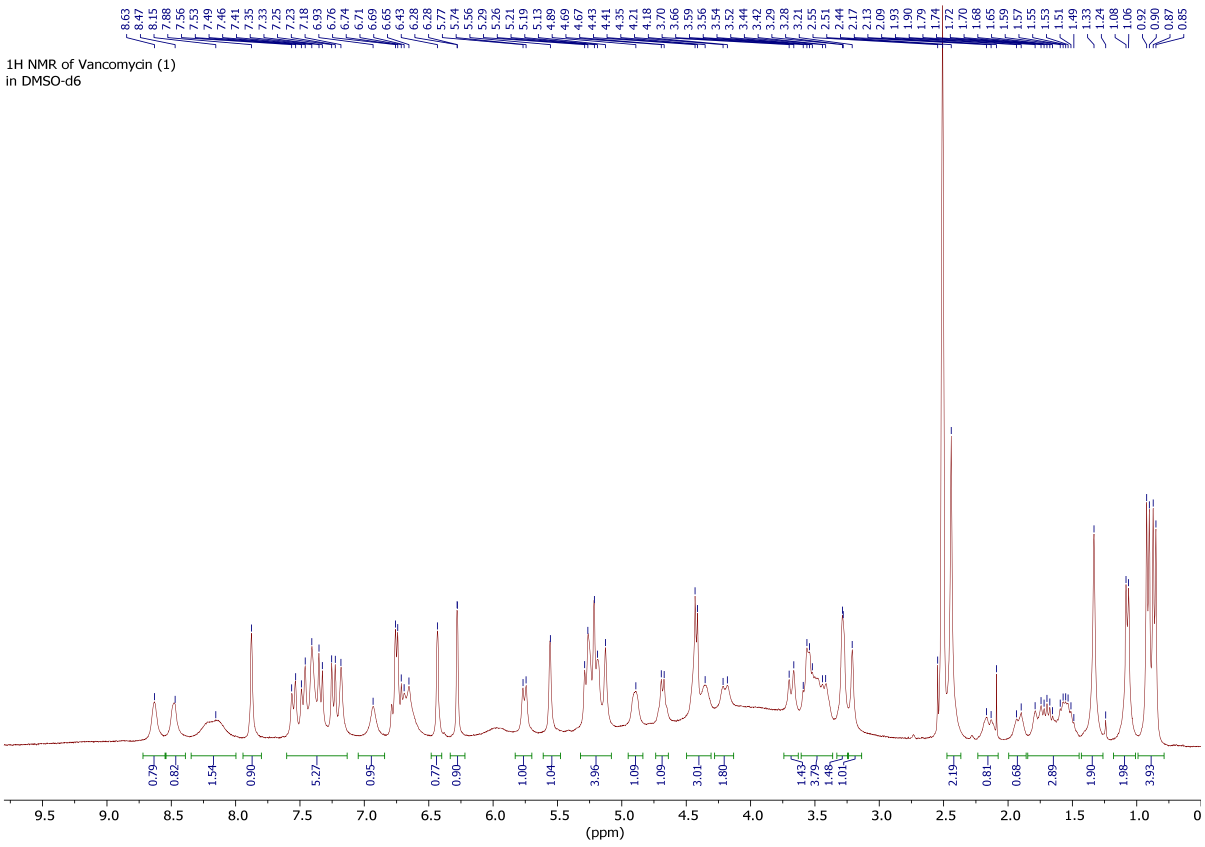

Figure S52. ^{13}C NMR spectrum of vancomycin (**1**)

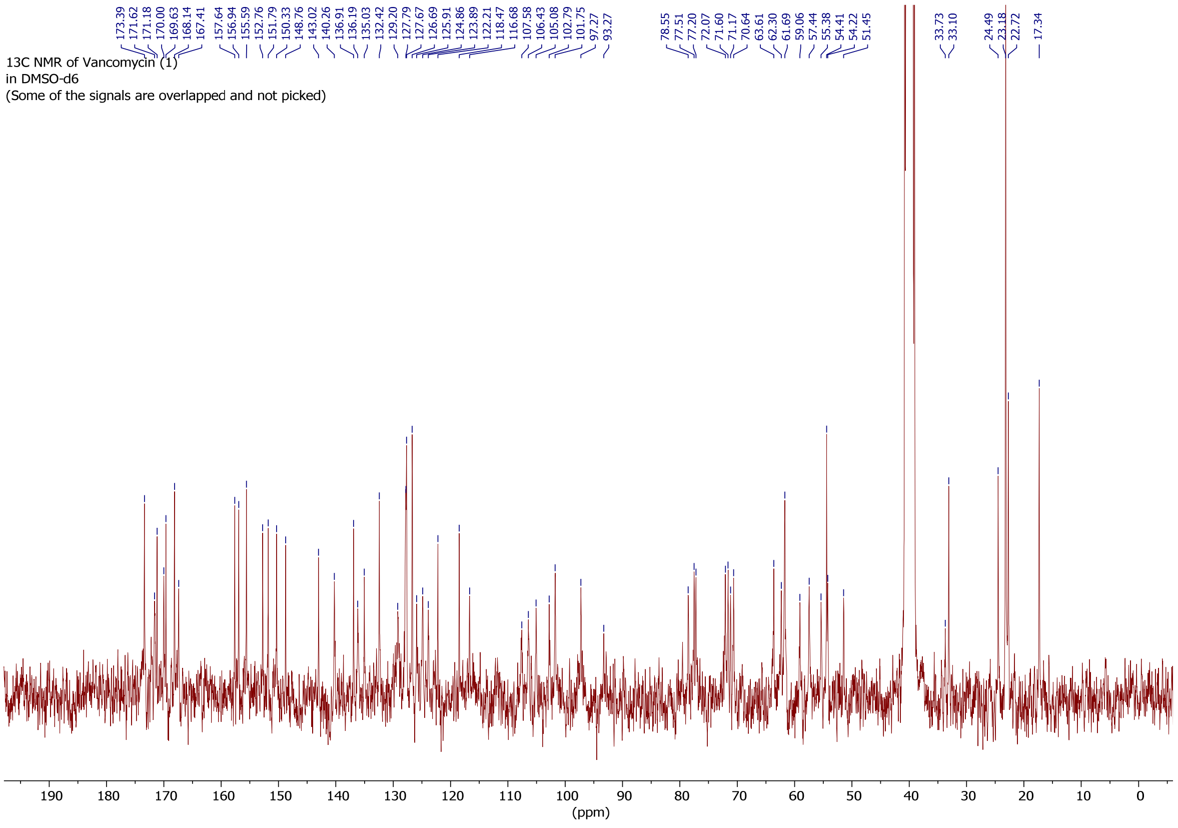

Figure S53. HSQC NMR spectrum of vancomycin (**1**)

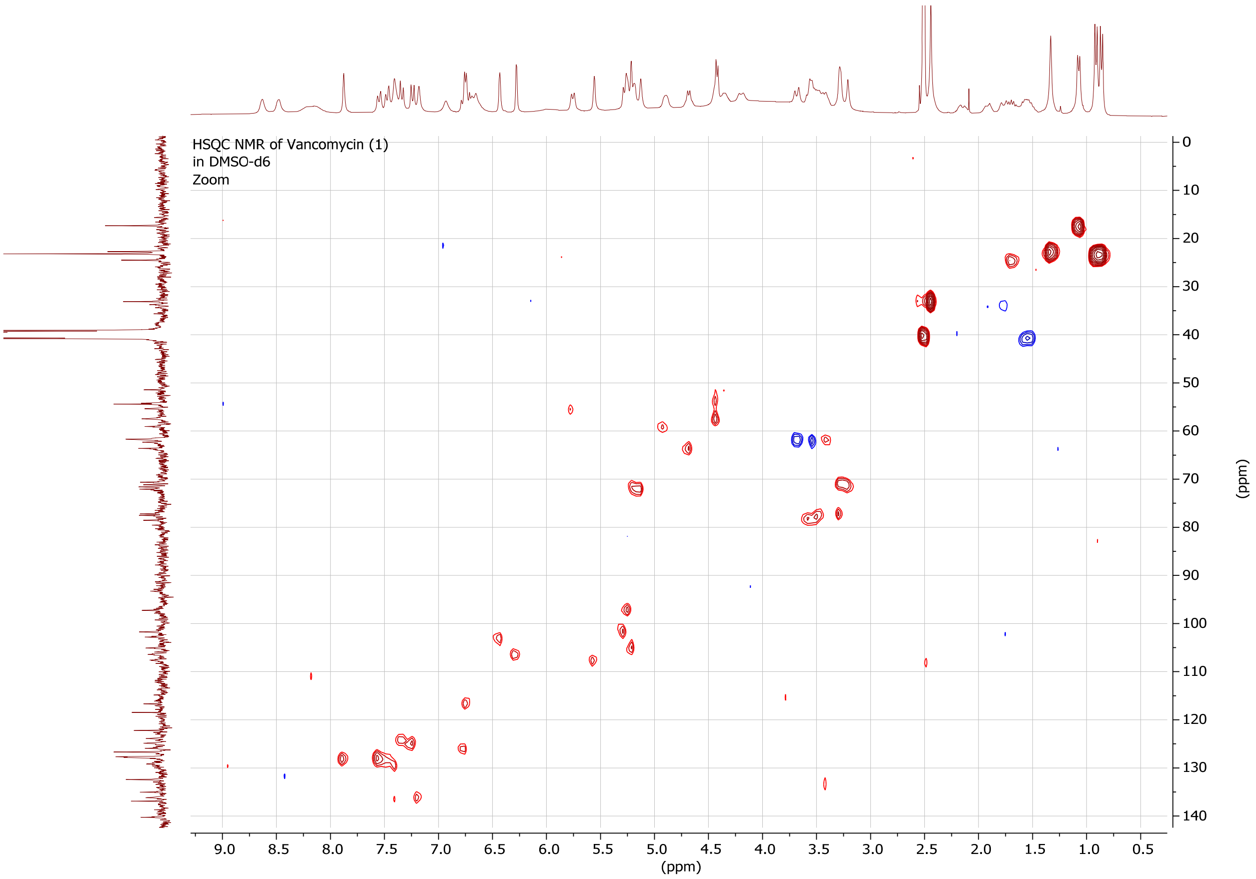

Supplement: Supplementary file 1 [file antibiotics-12-00894-s001.zip › antibiotics-2364796-supplementary.pdf]
